# Supplementary material for: Affinity‐Based Interactome Mapping of Inositol Pyrophosphates Reveals 4/6‐PP‐InsP5‐Binding Proteins in Plants
Source: Adv Sci (Weinh). 2026 Mar 2;13(26):e24290. doi: 10.1002/advs.202524290 (PMC13159116; doi:10.1002/advs.202524290)

Supporting Information

Affinity-Based Interactome Mapping of Inositol Pyrophosphates Reveals 4/6-PP-InsP$_5$-Binding Proteins in Plants

Kevin Ritter[1], Verena Gaugler[2], Sara Christina Stolze[3], Riya Ghosh[4], Akhila Jayamon[4], Ranjana Yadav[4], Felix Wollensack[1], Debabrata Laha[4], Hirofumi Nakagami[3], Gabriel Schaaf[2], Henning Jacob Jessen[1*]

[1] Faculty of Chemistry and Pharmacy, Institute of Organic Chemistry, and CIBSS-Centre for Integrative Biological Signaling Studies, Albert-Ludwigs University Freiburg, 79104 Freiburg, Germany

[2] Department of Plant Nutrition, Institute of Crop Science and Resource Conservation, Rheinische Friedrich-Wilhelms-Universität Bonn, 53115 Bonn, Germany

[3] Protein Mass Spectrometry, Max Planck Institute for Plant Breeding Research, 50829 Cologne, Germany

[4] Department of Biochemistry, Division of Biological Sciences, Indian Institute of Science (IISc), Bengaluru 560012, India

*Email: henning.jessen@oc.uni-freiburg.de

Table of Contents

1	General Remarks	2
2	Chemical Synthesis	4
3	Affinity Enrichment Experiments	19
4	Proteomics	21
5	Biophysical Validation of AtFHA2–Ligand Interactions	24
6	References	27
7	Gene Ontology Term Enrichment Analysis by g:Profiler	28
8	NMR – Spectra	36
9	Mass Spectra, CE Electropherograms & HPLC	70

1 General Remarks

Reactions were carried out using oven-dried glassware under an atmosphere of dry Argon and magnetically stirred, unless noted otherwise. Air- and moisture-sensitive liquids and solutions were transferred via syringe or stainless steel canula.

Reagents were purchased from commercial suppliers (Acros, Sigma-Aldrich, Fluka, TCI, BLDpharm, ChemPur, Alfa Aesar, VWR) and used without further purification, unless noted otherwise.

Solvents were obtained in analytical grade and used as received for extractions, precipitation and solid washings.

Dry solvents for reactions were purchased in a dry form from commercial suppliers (Sigma-Aldrich, Acros, Thermo Scientific) and stored over molecular sieves as well as under the atmosphere of dry Argon.

Deuterated solvents for NMR and reactions were obtained from commercial suppliers (Eurisotope and Deutero) in the indicated purity grade and used as received for NMR spectroscopy.

Thin layer chromatography (TLC) was performed with Merck silica gel 60 F_{254} . Compounds were visually analysed by UV light (λ = 254 and 365 nm) or stained. Staining solutions: $KMnO_4$ stain (1.5 g $KMnO_4$, 10 g Na_2CO_3, 1.25 mL 10% aq. NaOH, 200 mL H_2O), phosphomolybdic acid stain (PMA, 3-4 g $H_3PMo_{12}O_{40}$ in 200 mL EtOH).

Silica column chromatography was carried out using silica gel 60 (0.04 – 0.063 mm, 230 – 400 mesh) from Macherery-Nagel as stationary phase and with non-dry solvents.

Preparative RP-MPLC was performed using a PuriFlash 5.125 by Advion-Interchim. The stationary phase consists of C_{18} reversed-phase (15 µm or 30 µm) silica, prepacked in different column sizes and supplied by Advion-Interchim.

Lyophilizations were done with Christ Freeze Dryer Alpha 1-4 LDplus and Christ Freeze Dryer Alpha 1-2 LDplus.

^{1}H-NMR spectra were recorded on a Bruker 400 MHz (with Prodigy CryoProbe) spectrometer in the indicated deuterated solvent. Data are reported as follows: chemical shift (δ, ppm), multiplicity (s, singlet; d, doublet; t, triplet; q, quartet; m, multiplet; br. s, broad singlet), coupling constant(s) (J, Hz), integration. All signals were referenced to the internal solvent signal as standard (D_2O: δ = 4.79 ppm; MeCN-d_3: δ = 1.94 ppm, $CDCl_3$: δ = 7.26 ppm).

^{13}C{^{1}H}-NMR spectra were recorded with ^{1}H-broadband decoupling on a Bruker 101 MHz (with Prodigy CryoProbe) spectrometers at 298K in the indicated deuterated solvent. All signals were referenced to an internal standard.

^{31}P{^{1}H}-NMR spectra and **^{31}P-NMR** spectra were recorded with ^{1}H-broadband decoupling or ^{1}H-coupling on a 162 MHz spectrometer (with Prodigy CryoProbe) in the indicated deuterated solvent. All signals were referenced to an internal standard (PPP).

Mass spectra were recorded by C. Warth (Mass spectrometry service of the University of Freiburg) on a Thermo LCQ Advantage [spray voltage: 2.5 – 4.0 kV, spray current: 5 μA, ion transfer tube: 250 (150) °C, evaporation temperature: 50 – 400°C].

2 Chemical Synthesis

2.1 Synthesis of Phosphoramidites & Other Reagents

2.1.1 Synthesis of Bn-Phosphoramidite **S1**

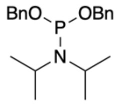

S1

$C_{20}H_{28}NO_2P$
MW: 345,42

The compound was synthesized according to Hofer et al. Analytical data were in accordance with literature.[1]

2.1.2 Synthesis of PCP-Phosphoramidite **S2**

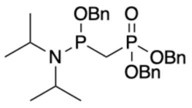

S2

$C_{28}H_{37}NO_4P_2$
MW: 513,5545

The compound was synthesized according to Hostachy et al. Analytical data were in accordance with literature.[2]

2.1.3 Synthesis of PEG-Linker Alcohol **S3**

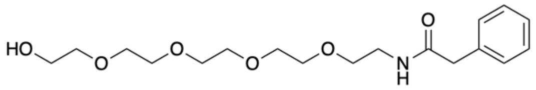

S3

$C_{18}H_{29}NO_6$
MW: 355,4310

The compound was synthesized according to Wu et al. Analytical data were in accordance with literature.[3]

2.1.4 Synthesis of Fm-Phosphordiamidite **S4**

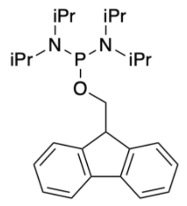

S4

$C_{26}H_{39}N_2OP$
MW: 426,5848

The compound was synthesized according to Ma et al. Analytical data were in accordance with literature.[4]

2.1.5 Synthesis of PEG-Linker-Phosphates **S5**

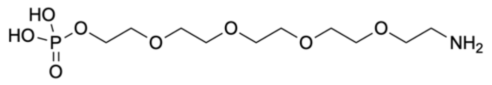

S5

$C_{10}H_{24}NO_8P$
MW: 317,2748

The compound was synthesized according to Wu et al. Analytical data were in accordance with literature.[3]

2.1.6 Synthesis of 4-PP-InsP$_5$ (**4**) & 6-PP-InsP$_5$ (**5**)

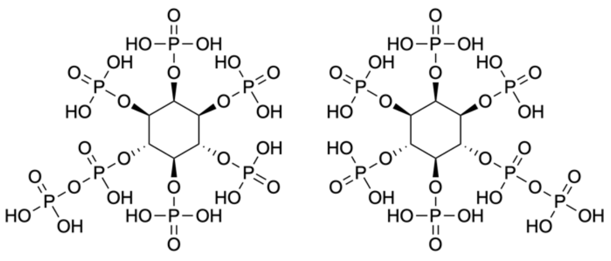

4 **5**

$C_6H_{19}O_{27}P_7$
MW: 740,0073

The compounds were synthesized according to Qiu et al. Analytical data were in accordance with the literature.[5]

2.2 Synthesis of Amino-PEG-4/6-PCP-InsP$_5$

2.2.1 Synthesis of TBS-protected Ortho Ester **S6**

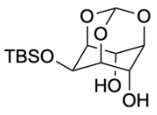

S6

$C_{13}H_{24}O_6Si$
MW: 304,4140

The compound was synthesized according to Baudin et al. Analytical data were in accordance with literature.[6]

2.2.2 Synthesis of Diallyl-Protected Ortho Ester **8**

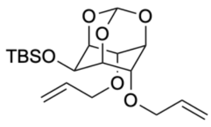

8

$C_{19}H_{32}O_6Si$
MW: 384,5440

TBS-protected ortho ester **S6** (1.00 g, 3.29 mmol) was dissolved in DMF (25 mL) and cooled to 0°C. Sodium hydride (342 mg, 8.54 mmol, 2.6 eq) was added portion-wise, and the mixture was stirred for 20 min at 0°C. Allyl bromide (738 µL, 1.03 g, 8.54 mmol, 2.6 eq) was added dropwise, along with a catalytic amount of sodium iodide (~50 mg). The reaction mixture was stirred overnight at room temperature, then diluted with EtOAc (200 mL). The solution was washed with water (100 mL) and brine (3 × 100 mL), and the layers were separated. The organic layer was dried over $MgSO_4$, and the solvent was removed under reduced pressure. The crude product was purified by silica column chromatography (cyclohexane/Et_2O 5:1), and the product **8** (1.25 g, 3.24 mmol, 99%) was obtained as a colorless oil.

^{1}H-NMR (400 MHz, $CDCl_3$): δ = 5.98 – 5.82 (m, 2H), 5.57 – 5.52 (m, 1H), 5.31 (dq, *J* = 17.2, 1.6 Hz, 2H), 5.21 (dp, *J* = 10.5, 1.5 Hz, 2H), 4.40 (tt, *J* = 3.4, 1.6 Hz, 1H), 4.34 – 4.29 (m, 1H), 4.29 – 4.24 (m, 2H), 4.17 – 4.10 (m, 4H), 4.06 (ddt, *J* = 12.9, 5.4, 1.5 Hz, 2H), 0.97 (s, 9H), 0.17 (s, 6H) ppm.
^{13}C-NMR (101 MHz, $CDCl_3$): δ = 134.17, 117.02, 103.18, 74.08, 73.36, 70.45, 68.16, 61.71, 26.00, 18.46, -4.65 ppm.
HRMS (APCI) $[M+H]^+$ calculated for $C_{19}H_{33}O_6Si$: 385.2041, found 385.2043.

2.2.3 Synthesis of Diallyl-protected Inositol **9**

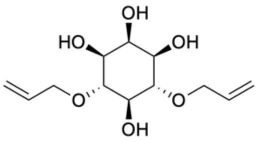

9

$C_{12}H_{20}O_6$
MW: 260,2860

Diallyl-protected ortho ester **8** (400 mg, 1.04 mmol) was dissolved in THF (10 mL), and aqueous HCl (1M, 10 mL) was added. The reaction mixture was stirred overnight at 40°C. After completion of the reaction cyclohexane (50 mL) and water (50 mL) were added, and the layers were separated. The organic layer was discarded, and the aqueous phase was concentrated under reduced pressure. The crude product was purified by silica column chromatography (CH_2Cl_2/MeOH 90:10), and the product **9** (197 mg, 0.757 mmol, 73%) was obtained as a colorless solid.

^{1}H-NMR (400 MHz, CD_3CN): δ = 5.98 (ddt, *J* = 17.4, 10.5, 5.7 Hz, 2H), 5.30 – 5.20 (m, 2H), 5.11 (ddt, *J* = 10.5, 2.1, 1.3 Hz, 2H), 4.33 – 4.19 (m, 4H), 3.85 (s, 1H), 3.40 – 3.22 (m, 6H), 3.19 (d, *J* = 3.4 Hz, 1H), 3.07 (d, *J* = 5.2 Hz, 2H) ppm.

^{13}C-NMR (101 MHz, CD_3CN): δ = 137.24, 116.44, 82.44, 75.43, 74.37, 73.71, 72.54 ppm.

HRMS (APCI) $[M-H]^-$ calculated for $C_{12}H_{19}O_6$: 259.1187, found 259.1182.

2.2.4 Synthesis of Protected Inositol Tetraphosphate **10**

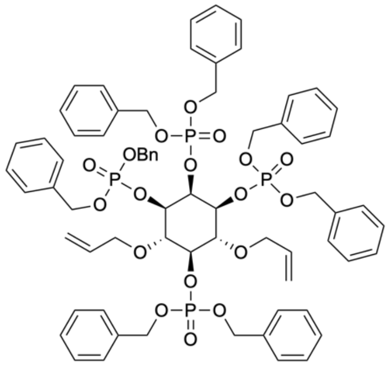

10

$C_{68}H_{72}O_{18}P_4$
MW: 1301,2010

Diallyl-protected Inositol **9** (179 mg, 688 µmol) was coevaporated twice with MeCN (3 mL) and dissolved in DMF (3 mL). Bn-phosphoramidite **S1** (1.85 g, 4.81 mmol, 7.0 eq) and DCI (948 mg, 4.81 mmol, 7.0 eq) were added, and the reaction mixture was stirred for 2 h. Reaction progress was monitored by ^{31}P-NMR. After the phosphitylation was completed, oxidation was done by *m*CPBA (70%, 1.19 g, 4.81 mmol, 7.0 eq) at 0°C, and the reaction mixture was stirred for 10 min at

room temperature. EtOAc (100 ml) and water (100 ml) were added, and the layers were separated. The organic layer was washed with water (100 ml) and brine (3 × 100 mL) and dried over $MgSO_4$. Celite was added, and the solvent was removed under reduced pressure to prepare the crude product as dry load for purification by automated reversed-phase MPLC (Interchim C_{18}-HP-Column, H_2O/MeCN, gradient: 20 - 100% MeCN). The product **10** (722 mg, 555 µmol, 81%) was obtained as a colorless oil.

^{1}H-NMR (400 MHz, $CDCl_3$): δ = 7.28 – 7.12 (m, 40H), 5.74 (ddt, *J* = 17.3, 10.4, 5.7 Hz, 2H), 5.27 (dt, *J* = 8.5, 2.4 Hz, 1H), 5.11 – 4.92 (m, 18H), 4.92 – 4.83 (m, 2H), 4.28 – 4.13 (m, 3H), 4.08 (dt, *J* = 5.8, 1.4 Hz, 4H), 3.71 (t, *J* = 9.6 Hz, 2H) ppm.
^{31}P-NMR (162 MHz, $CDCl_3$): δ = -1.38 (s, 2P), -1.59 (s, 1P), -1.93 (s, 1P) ppm.
^{13}C-NMR (101 MHz, $CDCl_3$): δ = 136.12 – 135.57 (m)*, 134.59, 128.67 – 127.51 (m)*, 117.16, 79.37 (d, *J* = 7.3 Hz), 77.21 (d, *J* = 5.0 Hz), 76.89 (dd, *J* = 5.6, 3.4 Hz), 75.34 (d, *J* = 7.0 Hz), 74.02, 70.57 – 68.37 (m)* ppm.
**Due to significant signal overlap, not all expected carbon resonances could be identified. Overlapping signals are listed as multiplets (m) and marked with an asterisk. Missing inositol signals are located below the $CDCl_3$ signal and were observed in HSQC.*
HRMS (ESI) $[M+Na]^+$ calculated for $C_{68}H_{72}O_{18}P_4Na$: 1323.3561, found 1323.3570.

2.2.5 Synthesis of Protected Inositol Tetraphosphate 11

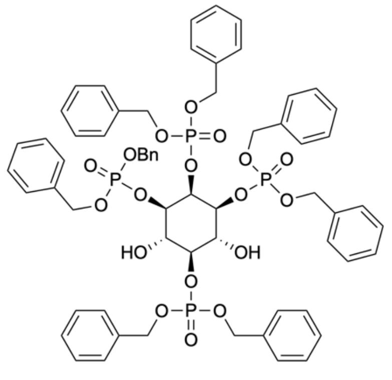

11

$C_{62}H_{64}O_{18}P_4$
MW: 1221,0710

Protected Inositol Tetraphosphate **10** (250 mg, 192 µmol) was dissolved in MeOH (10 mL) and $PdCl_2$ (75.7 mg, 384 mmol, 2.0 eq) was added. The reaction was stirred for 2h at room temperature. The reaction progress was monitored by ^{31}P-NMR. After completion of the reaction the mixture was diluted with EtOAc (100 mL) and washed with a saturated aqueous $NaHCO_3$-solution (2 × 100 mL) and brine (100 mL). The organic phase was dried over $MgSO_4$ and the solvent

was removed under reduced pressure. The product **11** (189 mg, 169 µmol, 88%) was obtained as a colorless solid was used without further purification.

^{1}H-NMR (400 MHz, $CDCl_3$): δ = 7.28 – 7.08 (m, 40H), 5.11 (dt, *J* = 8.9, 2.5 Hz, 1H), 5.07 – 4.89 (m, 16H), 4.39 – 4.32 (m, 4H), 4.25 (td, *J* = 9.0, 7.9 Hz, 1H), 3.91 (td, *J* = 9.4, 4.3 Hz, 2H) ppm.
^{31}P-NMR (162 MHz, $CDCl_3$): δ = -0.32 (s, 1P), -0.77 (s, 2P), -2.01 (s, 1P) ppm.
^{13}C-NMR (101 MHz, $CDCl_3$): δ = 135.99 – 135.36 (m)*, 128.75 – 127.60 (m)*, 81.62 (d, *J* = 5.9 Hz), 77.30 (d, *J* = 4.7 Hz), 75.94 (d, *J* = 4.6 Hz), 70.70 (t, *J* = 3.4 Hz), 70.20 – 69.22 (m)* ppm.
**Due to significant signal overlap, not all expected carbon resonances could be identified. Overlapping signals are listed as multiplets (m) and marked with an asterisk. Missing inositol signals are located below the $CDCl_3$ signal and were observed in HSQC.*
HRMS (ESI) $[M+NH_4]^+$ calculated for $C_{62}H_{68}NO_{18}P_4$: 1238.3381, found 1238.3385.

2.2.6 Synthesis of Protected Inositol Pentaphosphate **12**

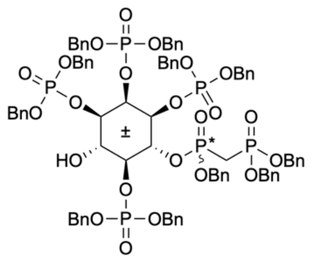

12

$C_{84}H_{86}O_{23}P_6$
MW: 1649,4316

Protected inositol tetraphosphate **11** (271 mg, 222 µmol) was coevaporated twice with MeCN (3 mL) and dissolved in CH_2Cl_2 (3 mL). PCP-phosphoramidite **S2** (137 mg, 266 mmol, 1.2 eq) and DCI (52.4 mg, 444 µmol, 2.0 eq) were added, and the reaction mixture was stirred for 1.5 h. Reaction progress was monitored by ^{31}P-NMR. After the phosphitylation was completed, oxidation was done by *m*CPBA (70%, 109 mg, 444 mmol, 2.0 eq) at 0°C, and the reaction mixture was stirred for 10 min at room temperature. Celite was added, and the solvent was removed under reduced pressure to prepare the crude product as dry load for purification by automated reversed-phase MPLC (Interchim C_{18}-HP-Column, H_2O/MeCN, gradient: 20 - 100% MeCN). The product **12** (186 mg, 113 µmol, 51%) was obtained as a colorless oil.

^{1}H-NMR (400 MHz, $CDCl_3$): δ = 7.29 – 7.02 (m, 55H), 5.40 – 5.26 (m, 1H), 5.23 – 4.68 (m, 24H), 4.19 (qt, *J* = 9.3, 2.2 Hz, 1H), 4.10 – 4.00 (m, 1H), 3.98 – 3.84 (m, 2H), 2.89 – 2.45 (m, 2H) ppm.
^{31}P-NMR (162 MHz, $CDCl_3$): δ = 21.11 (d, 2P), 20.52 – 19.40 (m, 2P), 0.66 and 0.41 (s, 1P), -1.06 and -1.23 (s, 1P), -1.34 and -1.36 (s, 1P), -2.25 (s, 1P) ppm.

The compound was obtained as a mixture of diastereomers arising from the formation of a stereogenic phosphorus center during phosphorylation. Duplication of some signals is observed in both the ^{1}H and ^{31}P NMR spectra, with summed integrals remaining consistent with the expected structure.

^{13}C-NMR (101 MHz, $CDCl_3$): δ = 136.41 – 134.62 (m)*, 129.44 – 127.07 (m)*, 80.52 – 79.92 (m), 76.54 – 76.15 (m), 75.57 – 74.93 (m), 73.93 – 73.59 (m), 73.37 (d, *J* = 22.2 Hz), 70.97 – 69.22 (m)*, 68.43 – 67.33 (m)*, 27.82 – 24.76 (m) ppm.

**Due to significant signal overlap, not all expected carbon resonances could be identified. Overlapping signals are listed as multiplets (m) and marked with an asterisk. Missing inositol signal is located below the benzyl signals and was observed in HSQC.*

HRMS (ESI) $[M+Na]^+$ calculated for $C_{84}H_{86}O_{23}NaP_6$: 1671.3878, found 1671.3861.

2.2.7 Synthesis of Protected Inositol Phosphate **13**

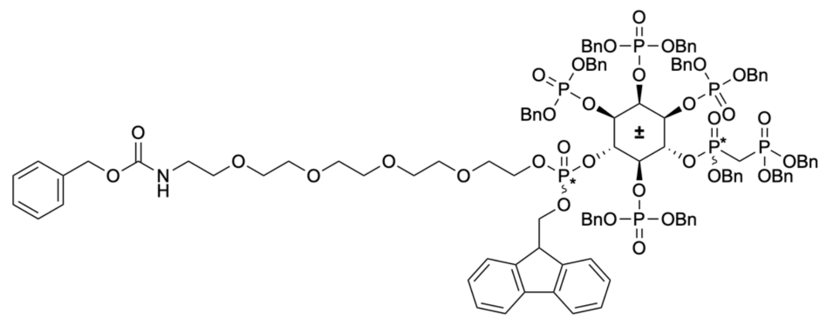

13

$C_{116}H_{124}NO_{32}P_7$
MW: 2261,0593

Fm-Phosphordiamidite **S4** (300 mg, 703 µmol, 6.2 eq) and PEG-Linker alcohol **S3** (261 mg, 703 µmol, 6.2 eq) were dissolved in MeCN (2 ml) and coevaporated twice with MeCN (2 ml). The mixture was dissolved in CH_2Cl_2 (1.3 mL) and a solution of ETT in CH_2Cl_2 (2.6 mL, 91.5 mg, 703 µmol, 6.2 eq) was added dropwise at 0°C. The reaction mixture was stirred for 30 min at room temperature. Reaction progress was monitored by ^{31}P-NMR. The resulting phosphoramidite was directly used without purification to phosphorylate protected inositol pentaphosphate **12.**

Protected inositol pentaphosphate **12** (186 mg, 113 µmol) was coevaporated twice with MeCN (2 ml) and dissolved in CH_2Cl_2 (3 ml). The solution was added dropwise to the phosphoramidite solution, and a solution of ETT in MeCN (1 M, 338 µl, 44.0 mg, 338 µmol, 3.0 eq) was added dropwise at 0°C. Reaction progress was monitored by ^{31}P-NMR. After the phosphitylation was completed, oxidation was done by *m*CPBA (70%, 83.4 mg, 338 µmol, 3.0 eq) at 0°C, and the reaction mixture was stirred for 10 min at room temperature. Celite was added, and the solvent was removed under reduced pressure to prepare the crude product as dry load for purification by

automated reversed-phase MPLC (Interchim C_{18}-HP-Column, H_2O/MeCN, gradient: 20 - 100% MeCN). The product **13** (179 mg, 79.2 µmol, 70%) was obtained as a colorless oil.

The compound was isolated as a mixture of four diastereomers in an undetermined ratio. Since the configuration at the stereocenters is removed in a subsequent deprotection step, the diastereomeric composition is not relevant for the final compound. Due to the overlap of signals arising from the diastereomeric mixture, the expected splitting patterns in the ^1H, 13C, and ^{31}P NMR spectra were not fully resolved. Nevertheless, integration confirmed that the relative intensities of the overlapping ^1H and ^{31}P signals are consistent with the expected structure. The ^{13}C NMR spectrum is included for completeness but no peak assignments are provided due to significant signal overlap from the PEG and benzyl protecting groups as well as from the diastereomeric mixture.

^{1}H-NMR (400 MHz, $CDCl_3$): δ = 7.77 – 7.02 (m, 68H), 5.70 – 5.48 (m, 1H), 5.36 – 4.79 (m, 27H), 4.59 – 4.34 (m, 1H), 4.34 – 3.86 (m, 7H), 3.62 – 3.01 (m, 18H), 3.00 – 2.60 (m, 2H) ppm.

^{31}P-NMR (162 MHz, $CDCl_3$): δ = 22.28 – 21.42 (1P, m), 20.63 – 19.95 (1P, m), -0.23 – -3.03 (5P, m) ppm.

HRMS (ESI) $[M+H]^+$ calculated for $C_{116}H_{125}O_{32}NP_7$: 2260.6343, found 2260.6401.

2.2.8 Synthesis of Amino-PEG-4/6-PCP-InsP$_5$ (**14**)

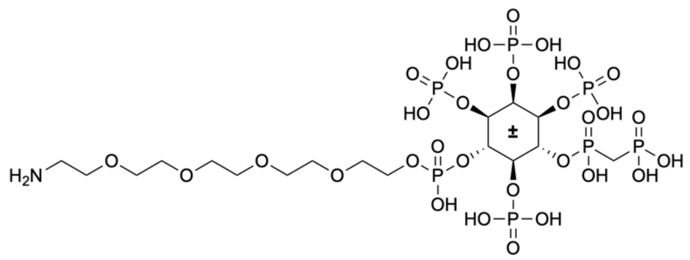

14

$C_{17}H_{42}NO_{30}P_7$
MW: 957,3163

Protected inositol phosphate **13** (20.0 mg, 8.85 µmol) was dissolved in a mixture of *t*BuOH and H_2O (3.6 mL, 40:7). $NaHCO_3$ (28.2 mg, 26.5 µmol, 13.0 eq) and Pd/C (10% on activated charcoal, 28.2 mg, 26.5 µmol, 3.0 eq) were added and the reaction was hydrogenated for 21h at 30 bar (autoclave). The reaction was interrupted and water (4 mL) was added to the mixture to ensure dissolution. The reaction was further hydrogenated for an additional 3h at 30 bar (autoclave). The catalyst was removed by filtration through a syringe filter. The filter was rinsed with water (2 mL). The solvent was removed by lyophilization and the product **14** (8.00 mg, 7.20 µmol, 81%) was obtained as a colorless solid without any further purification.

NMR samples contained diisopropylamine to improve resolution. Due to the low amount of compound, a direct ^{13}C-NMR measurement was not practical; therefore, ^{13}C-NMR resonances were assigned via the edHSQC spectrum. Two inositol protons overlapped with the water peak, as confirmed by edHSQC.

^{1}H-NMR (400 MHz, $CDCl_3$): δ = 4.58 – 4.47 (m, 3H), 4.39 (d, *J* = 12.0 Hz, 1H), 4.25 – 4.15 (m, 2H), 3.83 (t, *J* = 4.5 Hz, 2H), 3.81 – 3.71 (m, 12H), 3.66 (t, *J* = 5.3 Hz, 2H), 2.94 (t, *J* = 5.3 Hz, 2H), 2.24 – 2.11 (m, 2H) ppm.

^{31}P-NMR (162 MHz, $CDCl_3$): δ = 22.04 (d, *J* = 5.9 Hz, 1P), 13.27 (d, *J* = 6.0 Hz, 1P), 5.20 (s, 1P), 4.43 (s, 1P), 4.25 (s, 1P), 3.85 (s, 1P), -0.94 (s, 1P) ppm.

^{13}C-NMR (101 MHz, $CDCl_3$): δ = 74.76, 73.79, 73.47, 70.89, 70.57, 70.57, 69.60, 69.60, 65.73, 65.41, 39.62, 30.60, 29.31, 28.02 ppm.

HRMS (ESI) $[M-2H]^{2-}$ calculated for $C_{17}H_{40}NO_{30}P_7$: 477.4904, found 477.4892.

2.3 Synthesis of the Photoaffinity Linker

2.3.1 Synthesis of Aminohexanoic methyl ester (**S7**)

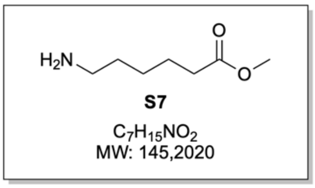

The compound was synthesized according to Tantisuwanno et al. Analytical data were in accordance with literature.[7]

2.3.2 Synthesis of Protected Lysine Derivative **16**

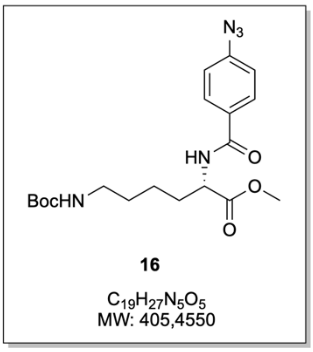

Boc-Lys-OMe · HCl (800 mg, 3.07 mmol) and 4-azidobenzoic acid (551 mg, 3.38 mmol, 1.1 eq) were dissolved in CH_2Cl_2 (25 ml). HOBt (498 mg, 3.69 mmol, 1.2 eq), EDCI · HCl (707 mg, 3.69 mmol, 1.2 eq) and triethyl amine (1.49 ml, 1.09 g, 10.8 mmol, 3.5 eq) were added, and the reaction mixture was stirred overnight at room temperature. The reaction mixture was dilluted with CH_2Cl_2 (25 ml), and washed with $KHSO_4$-solution (1 M, 2 × 50 mL) and saturated $NaHCO_3$-

solution (3 × 50 mL). The mixture was dried over $MgSO_4$ and the solvent was removed under reduced pressure. The crude product was purified by silica column chromatography (cyclohexane/EtOAc 1:1), and the product **16** (1.18 g, 2.90 mmol, 94%) was obtained as a beige solid.

^{1}H-NMR (400 MHz, $CDCl_3$): δ = 7.80 – 7.70 (m, 2H), 7.06 – 6.95 (m, 2H), 6.68 (d, *J* = 7.6 Hz, 1H), 4.71 (td, *J* = 7.6, 5.0 Hz, 1H), 4.53 (s, 1H), 3.71 (s, 3H), 3.04 (q, *J* = 6.5 Hz, 2H), 1.96 – 1.84 (m, 1H), 1.80 – 1.69 (m, 1H), 1.53 – 1.25 (m, 13H) ppm.
^{13}C-NMR (101 MHz, $CDCl_3$): δ = 173.07, 166.16, 156.13, 143.63, 130.34, 128.98, 119.02, 79.14, 52.53, 52.50, 40.02, 32.18, 29.69, 28.39, 22.50 ppm.
HRMS (ESI) $[M+Na]^+$ calculated for $C_{19}H_{27}N_5NaO_5$: 428.1904, found 428.1899.

2.3.3 Synthesis of Protected Peptide Derivative 17

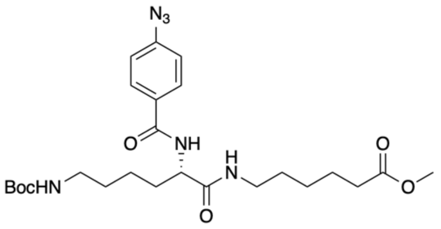

17

$C_{25}H_{38}N_6O_6$
MW: 518,6150

Protected lysine derivative **16** (400 mg, 987 mmol) was dissolved in MeOH (16 mL), and an aqueous solution of sodium hydroxide (1 M, 1.97 mL, 79.0 mg, 1.97 mmol, 2.0 eq) was added at 0°C. The reaction mixture was stirred for 30 min at 0°C. When the reaction was completed, $CHCl_3$ (100 mL) and water (50 mL) were added, and the pH was adjusted to 2-3 (HCl, 1 M). The layers were separated, and the aqueous layer was extracted with $CHCl_3$ (5 × 50 mL). The combined organic layers were dried over $MgSO_4$, and the solvent was removed under reduced pressure. The crude product was directly subjected to the following peptide coupling without further purification.
The crude product was dissolved in CH_2Cl_2 (8 mL) and aminohexanoic methyl ester (**S7**) (158 mg, 1.08 mmol, 1.1 eq), HOBt (160 mg, 1.18 mmol, 1.2 eq), EDCl · HCl (227 mg, 1.18 mmol, 1.2 eq) and triethylamine (478 µL, 349 mg, 3.45 mmol, 3.5 eq) were added. The reaction mixture was stirred overnight at room temperature. After completion of the reaction, the solvent was removed under reduced pressure. The residue was dissolved in EtOAc (50 mL), and the mixture was washed with an aqueous $KHSO_4$-solution (1 M, 2 × 50 mL), saturated aqueous $NaHCO_3$-solution (3 × 50 mL) and brine (50 mL). The mixture was dried over Na_2SO_4, and the solvent was removed under reduced pressure. The crude product was purified by silica column chromatography

(cyclohexane/EtOAc 1:2 to 1:4), and the product **17** (342 mg, 659 µmol, 67%) was obtained as a beige solid.

^{1}H-NMR (400 MHz, $CDCl_3$): δ = 7.81 – 7.70 (m, 2H), 7.05 (d, *J* = 7.8 Hz, 1H), 7.02 – 6.93 (m, 2H), 6.65 (q, *J* = 6.2 Hz, 1H), 4.67 (t, *J* = 6.0 Hz, 1H), 4.55 (td, *J* = 7.8, 5.8 Hz, 1H), 3.59 (s, 3H), 3.27 – 3.09 (m, 2H), 3.03 (dhept, *J* = 13.5, 6.8 Hz, 2H), 2.27 – 2.16 (m, 2H), 1.88 (dtd, *J* = 13.5, 7.7, 5.7 Hz, 1H), 1.80 – 1.64 (m, 1H), 1.61 – 1.16 (m, 19H) ppm.
^{13}C-NMR (101 MHz, $CDCl_3$): δ = 174.01, 171.65, 166.41, 156.17, 143.65, 130.26, 128.99, 118.98, 79.10, 53.47, 51.52, 40.04, 39.34, 33.79, 32.38, 29.66, 29.04, 28.41, 26.30, 24.38, 22.73 ppm.
HRMS (ESI) $[M\text{-}H]^-$ calculated for $C_{25}H_{37}N_6O_6$: 517.2780, found 517.2776.

2.3.4 Synthesis of Desthiobiotin Derivative **18**

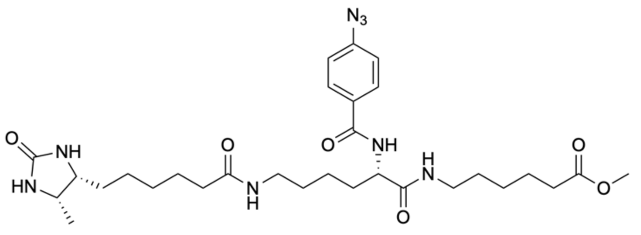

18
$C_{30}H_{46}N_8O_6$
MW: 614,7480

Protected peptide derivative **17** (227 mg, 438 µmol) was dissolved in CH_2Cl_2 (22 mL), and trifluoroacetic acid (11 mL) was added. The reaction mixture was stirred for 90 min at room temperature. When the reaction was completed, toluene (15 mL) was added. The solvent was removed under reduced pressure and the residue was coevaporated with toluene (10 mL). The crude product was used directly in the subsequent peptide coupling without further purification. The crude product was dissolved in DMF (11 mL) and desthiobiotin (103 mg, 481 µmol, 1.1 eq), HOBt (88.7 mg, 657 µmol, 1.5 eq), EDCI · HCl (126 mg, 657 µmol, 1.5 eq) and triethylamine (182 µL, 133 mg, 1.31 µmol, 3.0 eq) were added. The reaction mixture was stirred overnight at room temperature. After completion of the reaction, the reaction mixture was diluted with CH_2Cl_2 (6 mL) and washed with an aqueous $KHSO_4$ solution (1M, 2 × 10 mL), saturated aqueous $NaHCO_3$ solution (3 × 10 mL) and brine (2 × 10 mL). The organic layer was dried over Na_2SO_4, and the solvent was removed under reduced pressure. The crude product was purified by silica column chromatography (CH_2Cl_2/MeOH 95:5 to 9:1), and the product **18** (182 mg, 296 µmol, 68%) was obtained as a beige solid.

^{1}H-NMR (400 MHz, $CDCl_3$): δ = 7.94 – 7.86 (m, 2H), 7.73 (d, *J* = 8.1 Hz, 1H), 7.26 (t, *J* = 5.7 Hz, 1H), 7.12 – 7.04 (m, 2H), 6.48 (t, *J* = 5.4 Hz, 1H), 6.02 (s, 1H), 4.79 – 4.66 (m, 2H), 3.90 – 3.74 (m, 2H),

3.68 (s, 3H), 3.35 – 3.17 (m, 4H), 2.31 (t, *J* = 7.4 Hz, 2H), 2.28 – 2.11 (m, 2H), 1.96 – 1.71 (m, 3H), 1.70 – 1.27 (m, 17H), 1.13 (d, *J* = 6.1 Hz, 3H) ppm.

^{13}C-NMR (101 MHz, $CDCl_3$): δ = 174.14, 173.31, 172.24, 166.87, 163.59, 143.67, 130.09, 129.20, 118.96, 55.10, 53.04, 51.53*, 39.30, 38.44, 35.57, 33.88, 31.61, 29.24, 28.97, 28.13, 27.35, 26.34, 25.25, 24.74, 24.49, 22.52, 15.82 ppm.

** The HSQC spectrum reveals that this signal corresponds to two overlapping ^{13}C resonances.*

HRMS (ESI) $[M+Na]^+$ calculated for $C_{30}H_{46}N_8NaO_6$: 637.3433, found 637.3440.

2.3.5 Synthesis of Sulfo-NHS-Ester 19

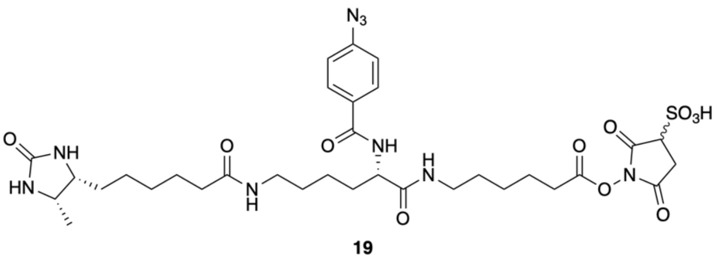

Desthiobiotin derivative **18** (50.0 mg, 81.3 µmol) was dissolved in MeOH (1.6 mL) and an aqueous solution of NaOH (1 M, 5 mL) was added at 0°C. The reaction mixture was stirred for 90 min at 0°C. When Reaction was completed $CHCl_3$ (30 mL) and water (30 mL) were added, and the pH was adjusted to 2-3 (HCl, 1 M). The layers were separated, and the aqueous layer was extracted with $CHCl_3$ (5 × 30 mL). The combined organic layers were dried over $MgSO_4$, and the solvent was removed under reduced pressure. The crude product was used directly in the subsequent reaction without further purification.

The crude product (35.0 mg, 58.3 µmol) was dissolved in DMF (1.6 mL), and *N*-hydroxysulfosuccinimide sodium salt (12.7 mg, 58.3 µmol, 1.0 eq) and DCC (36.1 mg, 175 µmol, 3.0 eq) were added. The reaction mixture was stirred for 48 h at room temperature. Reaction progress was monitored by HPLC. After completion of the reaction the solution was cooled to 4°C and the resulting solid was filtered off. A mixture of EtOAc and cyclohexane (1:1, 30 mL) was added to precipitate the product **19** (49.0 mg, 61.3 µmol, 75%) as a colorless solid.

^{1}H-NMR (400 MHz, DMSO-d_6): δ = 8.35 (d, *J* = 7.9 Hz, 1H), 8.02 – 7.85 (m, 3H), 7.77 – 7.64 (m, 1H), 7.29 – 7.14 (m, 2H), 6.27 (s, 1H), 6.10 (s, 1H), 4.39 – 4.29 (m, 1H), 3.69 (dd, *J* = 8.8, 2.4 Hz, 1H), 3.64 – 3.55 (m, 1H), 3.46 (td, *J* = 8.0, 5.7 Hz, 1H), 3.12 – 2.92 (m, 4H), 2.92 – 2.80 (m, 1H), 2.75 – 2.58 (m, 1H), 2.17 (t, *J* = 7.4 Hz, 1H), 2.01 (t, *J* = 7.4 Hz, 2H), 1.75 – 1.58 (m, 4H), 1.57 – 1.12 (m, 18H), 0.95 (d, *J* = 6.4 Hz, 3H).

^{13}**C-NMR** (101 MHz, DMSO-d_6): δ = 174.42, 171.88, 171.58, 171.33, 168.79, 167.99, 165.33, 165.30, 162.77, 142.27, 130.74, 129.46, 118.71, 56.27, 56.18, 54.94, 53.46, 50.19, 38.33, 38.15, 35.35, 33.65, 31.36, 30.92, 30.72, 30.11, 29.47, 28.87, 28.75, 28.71, 28.46, 25.87, 25.52, 25.30, 25.19, 24.17, 23.87, 15.46 ppm.

Additional ^{13}C NMR signals arise from the formation of diastereomeric sulfo-NHS esters. The use of racemic sulfo-NHS introduces a stereocenter, resulting in two species with distinct chemical environments.

HRMS (ESI) [M-H]$^-$ calculated for $C_{33}H_{46}N_9O_{11}S$: 776.3043, found 776.3048.

2.4 Synthesis of the Photoaffinity Reagents

2.4.1 Synthesis of Photoaffinity Reagent **20** (Custom Linker)

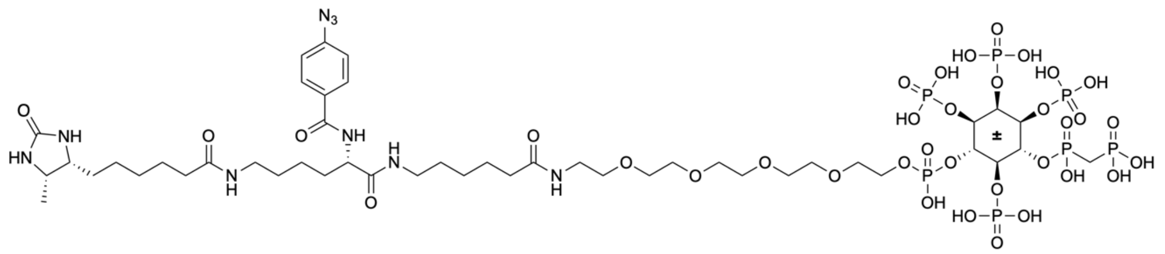

20

$C_{46}H_{84}N_9O_{35}P_7$
MW: 1540,0223

Amino-PEG-4/6-PCP-InsP$_5$ (**14**) (5.00 mg, 4.50 µmol) was dissolved in a $NaHCO_3$ buffer (0.2 M, 500 µL). A solution of Sulfo-NHS-Ester **19** (10 mg/mL, 1.0 mL, 10.0 mg, 12.5 µmol, 2.8 eq) was added and the reaction mixture was stirred for 2h at room temperature. The mixture was directly subjected to purification by automated reversed-phase MPLC (Interchim C_{18}-HP-Column, H_2O/MeCN, gradient: 20 - 90% MeCN, 10% TEAA buffer). The product **20** was obtained as a colorless oil (7.00 mg, 3.11 µmol, 69%) in form of the triethylammonium salt.

Due to extensive signal overlap with solvent (MeOD-d_4, H_2O), triethylammonium counterions, and the PEG linker region, only selected resonances could be confidently assigned. Characteristic signals, such as aromatic protons, the desthiobiotin methyl group, and triethylammonium peaks, were observed as expected. Resonances associated with the PEG linker and the inositol core overlap with solvent and counterion signals and could not be individually resolved. Full spectra are provided. Compound identity was confirmed by ^{31}P NMR, HRMS, and analytical HPLC. The inositol ^{13}C signals are only visible in HSQC spectra, as ^{31}P – ^{13}C coupling causes broadening in direct ^{13}C NMR. ^{13}C signals marked with m indicate overlapping resonances.

^{1}**H-NMR** (400 MHz, MeOD-d_4): δ = 7.98 – 7.90 (m, 1H), 7.21 – 7.13 (m, 1H), 5.34 (ddd, *J* = 5.6, 4.4, 1.1 Hz, 0H), 5.16 (d, *J* = 10.2 Hz, 0H), 4.71 – 4.62 (m, 0H), 4.57 (q, *J* = 9.6 Hz, 0H), 4.50 – 4.41 (m, 0H), 4.35 – 4.09 (m, 2H), 3.87 – 3.75 (m, 0H), 3.75 – 3.46 (m, 8H), 2.53 – 2.10 (m, 5H), 2.09 – 2.00 (m, 1H), 1.10 (d, *J* = 6.5 Hz, 2H) ppm.

^{31}P-NMR (162 MHz, MeOD-d_4): δ = 19.81 (d, 1P, J = 11.2 Hz), 13.99 (d, 1P, J = 11.3 Hz), 0.87 (s, 2P), 0.76 (s, 1P) , 0.36 (s, 1P), 0.06 (s, 1P) ppm.

^{13}C-NMR (101 MHz, MeOD-d_4): δ = 177.90, 174.86, 174.75, 173.01, 167.62, 164.77, 143.65, 130.38, 129.19, 118.59, 70.22 – 68.90 (m), 55.98, 54.13, 51.29, 38.93, 38.74, 38.58, 35.61, 35.51, 35.13, 34.80, 31.65, 31.48, 29.66 – 28.48 (m), 26.72, 26.14, 25.72, 25.56 – 25.41 (m), 25.24, 24.60, 23.10, 22.32, 14.24, 13.02, 7.85 ppm.

HRMS (ESI) $[M-2H]^{2-}$ calculated for $C_{46}H_{82}N_9O_{35}P_7$: 768.6544, found 768.6544.

2.4.2 Synthesis of Photoaffinity Reagent **21** (Commercial Linker)

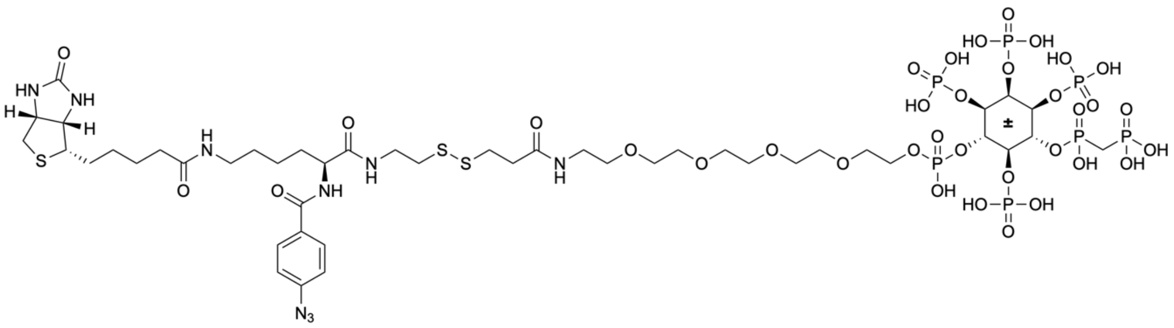

21

$C_{45}H_{80}N_9O_{35}P_7S_3$
MW: 1620,1593

Amino-PEG-4/6-PCP-InsP$_5$ (**14**) (4.00 mg, 3.60 µmol) was dissolved in a $NaHCO_3$ buffer (0.4 M, 400 µL). A solution of commercial Sulfo-SBED linker (10 mg/mL, 500 µL, 5.00 mg, 5.68 µmol, 1.6 eq) was added and the reaction mixture was stirred for 30 min at room temperature. The mixture was directly subjected to purification by automated reversed-phase MPLC (Interchim C$_{18}$-HP-Column, H_2O/MeCN, gradient: 20 - 90% MeCN, 10% TEAA buffer). The product **21** was obtained as a colorless oil (5.00 mg, 2.58 µmol, 72%) in form of the triethylammonium salt.

Due to extensive signal overlap with solvent (MeOD-d$_4$, H_2O), triethylammonium counterions, and the PEG linker region, only selected resonances could be confidently assigned. Characteristic signals, such as aromatic protons and triethylammonium peaks, were observed as expected. Resonances associated with the PEG linker and the inositol core overlap with solvent and counterion signals and could not be individually resolved. Full spectra are provided. Compound identity was confirmed by ^{31}P NMR, HRMS, and analytical HPLC. The inositol ^{13}C signals are only visible in HSQC spectra, as ^{31}P – ^{13}C coupling causes broadening in direct ^{13}C NMR. ^{13}C signals marked with m indicate overlapping resonances.

^{1}H-NMR (400 MHz, MeOD-d_4): δ = 8.00 – 7.90 (m, 1H), 7.20 – 7.14 (m, 1H), 5.19 (d, J = 11.0 Hz, 1H), 4.72 – 4.56 (m, 1H), 4.53 – 4.44 (m, 1H), 4.39 – 4.18 (m, 3H), 3.77 – 3.42 (m, 9H), 3.31 (dt, J = 3.3, 1.6 Hz, 19H), 3.00 – 2.83 (m, 2H), 2.78 – 2.67 (m, 1H), 2.67 – 2.60 (m, 1H), 2.45 – 2.30 (m, 1H), 2.18 (t, J = 7.2 Hz, 1H), 1.99 – 1.78 (m, 1H), 1.78 – 1.36 (m, 4H) ppm.

^{31}**P-NMR** (162 MHz, MeOD-*d₄*): δ = 20.18 (s, 1P), 13.37 (s, 1P), 1.13 (s, 1P), 0.94 (s, 1P), 0.59 (s, 1P), 0.05 (s, 1P), -0.40 (s, 1P) ppm.

^{13}**C-NMR** (101 MHz, MeOD-d_4): δ = 174.65, 173.33, 172.41, 167.71, 164.71, 143.67, 130.35, 129.22, 118.60, 70.21 – 69.76 (m), 69.10, 61.96, 60.24, 55.85 – 55.30 (m), 54.20, 39.67, 39.08, 38.61, 38.23, 37.03, 35.42, 35.21, 33.87, 31.36, 28.70, 28.05, 25.51, 25.48, 23.12 ppm.

HRMS (ESI) $[M-2H]^{2-}$ calculated for $C_{45}H_{78}N_9O_{35}P_7S_3$: 808.5968, found 808.5968.

3 Affinity Enrichment Experiments

3.1 Buffer Compositions

Wash Buffer:

- 50 mM Tris pH 7.5
- 100 mM NaCl
- 10 % glycerol (v/v)
- 1 mM $MgCl_2$

Extraction Buffer:

- 50 mM Tris pH 7.5
- 100 mM NaCl
- 10 % Glycerol
- 1 mM $MgCl_2$
- 1 % Triton X-100 (v/v)
- 5 mM DTT (only Affi-Gel and photoaffinity reagent custom)
- 10 µL/mL protease inhibitor cocktail (Sigma #P9599)
- 20 µL/mL phosphatase inhibitor cocktail (Sigma #5726)
- 20 µL/mL phosphatase inhibitor cocktail (Sigma #P0044)

3.2 Affi-Gel Based Affinity Enrichment Experiments

Plants were grown as described by Schneider et al.[8] Root and shoot tissues (~6 g) were harvested and pulverized in liquid nitrogen. The powdered material was extracted with 8–10 mL of the extraction buffer (composition provided in chapter 3.1). The suspension was incubated for 30 minutes on ice. Lysates were clarified by three consecutive centrifugation steps (15 minutes at 20.000 g, 4°C), transferring the supernatant to a new tube after each step. The final lysate was kept on ice. Protein concentrations were quantified using the Pierce™ 660 nm Protein Assay Reagent (Thermo Scientific) according to the manufacturer's protocol. Samples were diluted 1:10 in milliQ water prior to analysis. Average protein concentrations were ~0.5 mg/mL for root lysates and 1–2 mg/mL for shoot lysates.

Affinity reagents were prepared by coupling either Amino-PEG-4/6-PCP-$InsP_5$ (**12**) or PEG-Linker-Phosphates **S5** to Affi-Gel beads as described by Furkert et al.[9] and stored at 4°C until use. For pull-down experiments, Affi-Gel beads (200 µL) were washed twice with 1 mL of wash buffer (centrifuged for 3 minutes at 2000 g, supernatant discarded). The beads were then incubated with

1 mL of plant lysate at 4°C for 1 hour on an overhead shaker. After incubation, the beads were washed three times with 1 mL of wash buffer (each wash: 10 minutes on an overhead shaker, centrifuged for 3 minutes at 2000 g, supernatant discarded). Following the final wash, the beads were incubated with 100 µL of wash buffer supplemented with 20 mM $InsP_6$ for 1 hour at 4°C on an overhead shaker. Beads and supernatant were then separated, frozen in liquid nitrogen, and stored at -80°C.

Experiments were conducted with both the 4-/6-PP-$InsP_5$ affinity reagent and the phosphate control reagent under identical conditions.

3.3 Photoaffinity Based Affinity Enrichment Experiments

Plants were grown as described by Schneider et al.[8] Root and shoot tissues (~6 g) were harvested and pulverized in liquid nitrogen. The powdered material was extracted with 8–10 mL of extraction buffer (composition provided in chapter 7.3.3.1) and incubated for 30 minutes on ice. Lysates were clarified by three consecutive centrifugation steps (15 minutes at 20,000 g, 4°C), transferring the supernatant to a new tube after each step. The final lysate was kept on ice. Protein concentrations were quantified using the Pierce™ 660 nm Protein Assay Reagent (Thermo Scientific) according to the manufacturer's protocol. Samples were diluted 1:10 in milliQ water prior to analysis. Average protein concentrations were ~0.5 mg/mL for root lysates and 1–2 mg/mL for shoot lysates.

Photoaffinity experiments were conducted using two different reagents: Photoaffinity Reagent **18** (Custom Linker) and Photoaffinity Reagent **19** (Commercial Linker). For all experiments with Photoaffinity Reagent **19** (Commercial Linker), extraction buffers without DTT were used. Streptavidin-coated magnetic beads (90 µL) were washed twice with 600 µL of wash buffer (composition provided in Chapter 3.1) using a magnetic rack to remove the supernatant after each wash. For competition experiments, 700 µL of lysate were pre-incubated with 50 µL of competitor stock solution (10 mM, either 4-PP-$InsP_5$ or 6-PP-$InsP_5$) for 45 minutes at 4°C under rotation. For control experiments without competitor, 50 µL of water were added instead and incubated under identical conditions. Subsequently, 5 µL of the capture compound stock solution (400 µM) were added and the mixture incubated for 1 hour at 4°C under rotation. Crosslinking was performed by irradiating the mixture under a 365 nm UV lamp for 30 minutes at 4°C. The crosslinked mixture was then added to the prepared beads and incubated for 1 hour at 4°C under rotation. After binding, the beads were washed twice with 600 µL of wash buffer (each wash: magnetic rack, supernatant discarded). Following the final wash, the beads were frozen in liquid nitrogen and stored at -80°C until further processing.

4 Proteomics

4.1 Sample preparation

4.1.1 On-bead digest for Affi-Gel and Photoaffinity enrichments

Enriched proteins were submitted to an on-bead digestion using trypsin. In brief, dry beads were re-dissolved in 25 µL digestion buffer 1 (50 mM Tris, pH 7.5, 2M urea, 1mM DTT, 5 ng/µL trypsin) and incubated for 30 min at 30 °C in a Thermomixer with 400 rpm. Next, beads were pelleted and the supernatant was transferred to a fresh tube. Digestion buffer 2 (50 mM Tris, pH 7.5, 2M urea, 5 mM CAA) was added to the beads, after mixing the beads were pelleted, the supernatant was collected and combined with the previous one. The combined supernatants were then incubated overnight at 32 °C in a Thermomixer with 400 rpm; samples were protected from light during incubation. The digestion was stopped by adding 5 µL 20% TFA and desalted with C_{18} Empore disk membranes according to the StageTip protocol[10] and dried in a vacuum evaporator.

4.1.2 Sample preparation (in-solution digest for Affigel elutions)

The eluted protein mixtures were reduced with dithiothreitol, alkylated with chloroacetamide, and digested with trypsin (0.125 µg). These digested samples were desalted using with C_{18} Empore disk membranes according to the StageTip protocol[10] and dried in a vacuum evaporator.

4.2 LC-MS/MS data acquisition

Dried peptides were re-dissolved in 2% MeCN, 0.1% TFA (10 µL) for analysis. Streptavidin pulldown samples were analyzed using an EASY-nLC 1200 (Thermo Fisher) coupled to a Q Exactive Plus mass spectrometer (Thermo Fisher). Peptides were separated on 16 cm frit-less silica emitters (MSWil, 75 µm inner diameter), packed in-house with reversed-phase ReproSil-Pur C_{18} AQ 1.9 µm resin (Dr. Maisch). Peptides were loaded on the column and eluted for 115 min using a segmented linear gradient of 5% to 95% solvent B (0 min : 5%B; 0-5 min -> 5%B; 5-65 min -> 20%B; 65-90 min ->35%B; 90-100 min -> 55%; 100-105 min ->95%, 105-115 min ->95%) (solvent A 0% MeCN, 0.1% FA; solvent B 80% MeCN, 0.1%FA) at a flow rate of 300 nL/min. Mass spectra were acquired in data-dependent acquisition mode with a TOP15 method. MS spectra were acquired in the Orbitrap analyzer with a mass range of 300–1750 m/z at a resolution of 70,000 FWHM and a target value of 3×10^6 ions. Precursors were selected with an isolation window of 1.3 m/z. HCD fragmentation was performed at a normalized collision energy of 25. MS/MS spectra were acquired with a target value of 10^5 ions at a resolution of 17,500 FWHM, a maximum injection time (max.) of 55 ms and a fixed first mass of m/z 100. Peptides with a charge of +1, greater than

6, or with unassigned charge state were excluded from fragmentation for MS^2, dynamic exclusion for 30s prevented repeated selection of precursors.

Affi-Gel samples were analyzed using an Ultimate 3000 RSLC nano (Thermo Fisher) coupled to an Orbitrap Exploris 480 mass spectrometer equipped with a FAIMS Pro interface for Field asymmetric ion mobility separation (Thermo Fisher). Peptides were pre-concentrated on an Acclaim PepMap 100 pre-column (75 µM x 2 cm, C_{18}, 3 µM, 100 Å, Thermo Fisher) using the loading pump and buffer A** (water, 0.1% TFA) with a flow of 7 µl/min for 5 min. Peptides were separated on 16 cm frit-less silica emitters (MSWil, 75 µm inner diameter), packed in-house with reversed-phase ReproSil-Pur C18 AQ 1.9 µm resin (Dr. Maisch). Peptides were loaded on the column and eluted for 130 min using a segmented linear gradient of 5% to 95% solvent B (0 min : 5%B; 0-5 min -> 5%B; 5-65 min -> 20%B; 65-90 min ->35%B; 90-100 min -> 55%; 100-105 min ->95%, 105-115 min ->95%, 115-115.1 min -> 5%, 115.1-130 min ->5%) (solvent A 0% MeCN, 0.1% FA; solvent B 80% MeCN, 0.1%FA) at a flow rate of 300 nL/min. Mass spectra were acquired in data-dependent acquisition mode with a TOP_S method using a cycle time of 2 seconds. For Field asymmetric ion mobility separation (FAIMS) two compensation voltages (-45 and -60) were applied, the cycle time for each experiment was set to 1 second. MS spectra were acquired in the Orbitrap analyzer with a mass range of 320–1200 m/z at a resolution of 60,000 FWHM and a normalized AGC target of 300%. Precursors were filtered using the MIPS option (MIPS mode = peptide), the intensity threshold was set to 5000, Precursors were selected with an isolation window of 1.6 m/z. HCD fragmentation was performed at a normalized collision energy of 30%. MS/MS spectra were acquired with a target value of 75% ions at a resolution of 15,000 FWHM, at an automated injection time and a fixed first mass of m/z 100. Peptides with a charge of +1, greater than 6, or with unassigned charge state were excluded from fragmentation for MS^2, dynamic exclusion for 40s prevented repeated selection of precursors.

4.3 Data analysis

Raw data were processed using MaxQuant software (version 1.6.3.4, http://www.maxquant.org/)[11] with label-free quantification (LFQ) and iBAQ enabled.[12]

MS/MS spectra were searched by the Andromeda search engine against a combined database containing the sequences from *A. thaliana* (TAIR10_pep_20101214*) and sequences of 248 common contaminant proteins and decoy sequences. Trypsin specificity was required and a maximum of two missed cleavages allowed. Minimal peptide length was set to seven amino acids. Carbamidomethylation of cysteine residues was set as fixed, oxidation of methionine and protein N-terminal acetylation as variable modifications. The match between runs option was enabled. Peptide-spectrum-matches and proteins were retained if they were below a false discovery rate of 1% in both cases.

Statistical analysis of the MaxLFQ values was carried out using Perseus (version 1.5.8.5, http://www.maxquant.org/). Quantified proteins were filtered for reverse hits and hits "identified by site" and MaxLFQ values were log2 transformed. After grouping samples by condition only those proteins were retained for the subsequent analysis that had two valid values in one of the conditions. Two-sample t-tests were performed using a permutation-based FDR of 5%. Alternatively, quantified proteins were grouped by condition and only those hits were retained that had three valid values in one of the conditions. Missing values were imputed from a normal distribution (1.8 downshift, separately for each column). Volcano plots were generated in Perseus using an FDR of 5% and an S0=1. The Perseus output was exported and further processed using Excel.

* ftp://ftp.arabidopsis.org/home/tair/Proteins/TAIR10_protein_lists/

5 Biophysical Validation of AtFHA2–Ligand Interactions

5.1 Protein Purification

The coding sequence of AtFHA2 was cloned into an N-terminal His–MBP–tagged pET28b and His-tagged pET28b expression vector. *E. coli* RIL cells were transformed with the AtFHA2 constructs. The bacterial transformants were cultured at 37 °C overnight the secondary culture allowed to grow at 37 °C until the optical density at 600 nm (OD_{600}) reached ~0.8. Next, the culture was treated with 0.5 mM IPTG and allowed to grow for 18 h at 16C. For purification, cells were lysed in Tris–HCl buffer (500 mM NaCl, 50 mM Tris–HCl, pH 8.0, 5 % glycerol, 0.2 MM PMSF, 2 mM β-ME, and 0.1 % Tween-20). The lysates were centrifuged at 14 000 rpm for 45 min at 4 °C, and the supernatant was collected. The supernatant was then incubated with pre-equilibrated Ni–NTA beads (Qiagen, 30230) at 4 °C for 12 h. Bound protein was eluted using elution buffer (50 mM Tris-Cl pH 8.0, 500 mM NaCl and 250 mM Imidazole). For size-exclusion chromatography, the eluted fractions were injected into an FPLC system (AKTAPurifier) and passed through a HiLoad 16/600 Superdex 200 pg column. The eluted fractions were analyzed by SDS-PAGE and coomassie blue staining. The final protein fractions were pooled and concentrated to 0.4–0.5 mg mL^{-1}.

5.2 Circular dichroism (CD)

CD measurements were carried out using a Chirascan-plus CD spectrometer. Protein samples were prepared in 1× PBS (pH 7.4). AtFHA2–His–MBP protein was dialyzed and concentrated to 13 μM. Experiments were performed at room temperature. The UV CD spectra were recorded in the presence or absence of $InsP_6$ (500 μM and 1 mM), in the range of 190–320 nm using a 0.1 cm quartz cuvette.

5.3 Isothermal Titration Calorimetry

Isothermal titration calorimetry (ITC) experiments were performed using a MicroCal PEAQ-ITC instrument at 25 °C as described previously.[13] Purified His6-FHA2 recombinant protein (see 5.2) was dialyzed against buffer (50 mM Tris Cl pH 8.0 and 500 mM NaCl). All ligands were diluted in same buffer prior to all measurements. A typical titration consisted of 13-19 injections, the protein and ligand concentrations in the syringe and in the cell are provided in the respective figure legend. Control titrations of ligand and buffer were performed under similar conditions. The data were processed using the built-in software of MicroCal instruments (version 1.21).

5.4 Additional ITC Datasets and Control Experiments

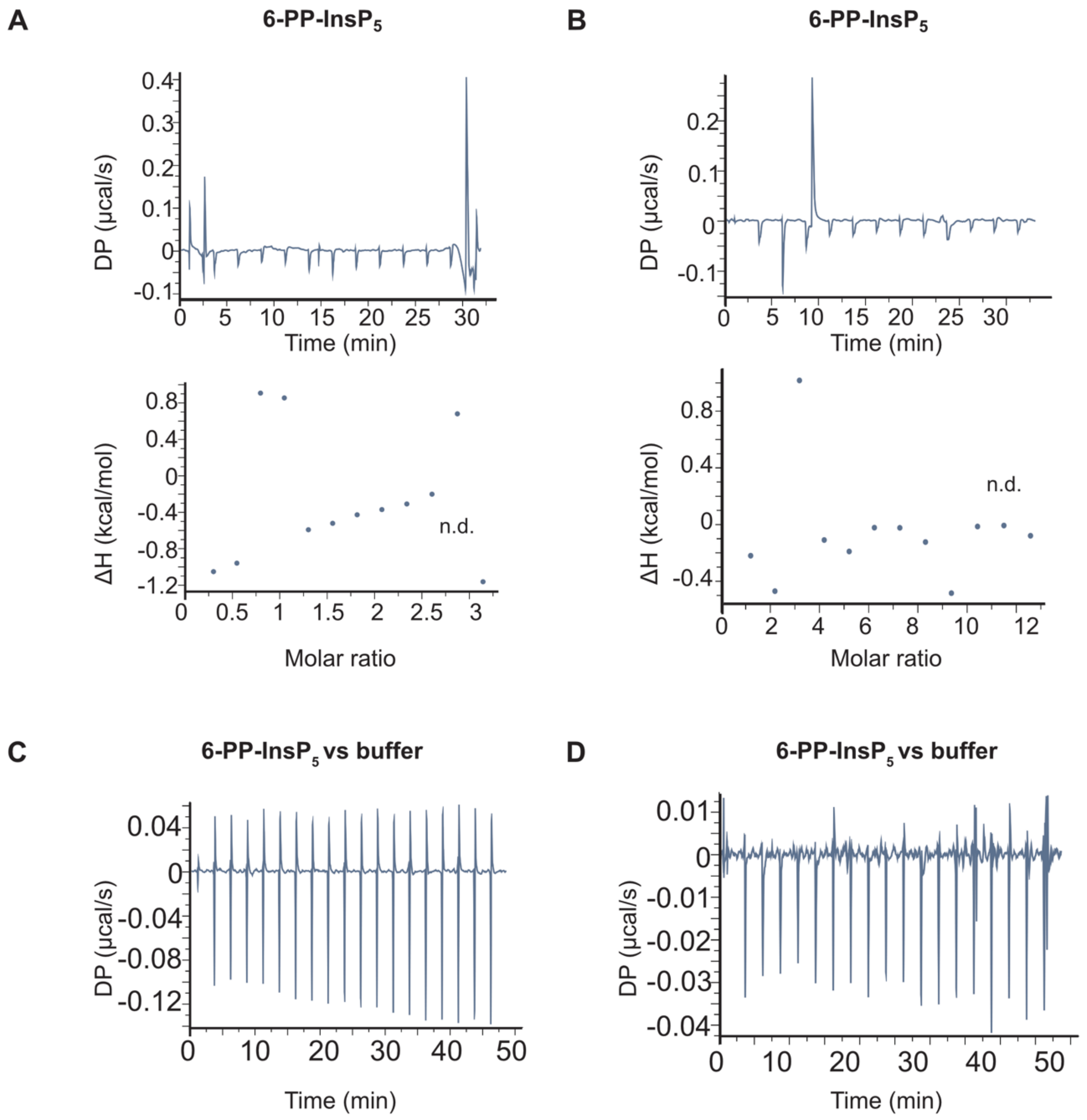

Figure S1: ITC experiments of AtFHA2 with different concentration of 6-PP-InsP$_5$.
ITC analysis of 6-PP-InsP$_5$ (250 µM; panel A and 1 mM; panel B) with recombinant AtFHA2 shown as buffer-subtracted raw heat traces (top) and integrated binding isotherm (bottom) (n.d., no detectable binding). Isothermal titration calorimetry (ITC) assays of (C) 6-PP-InsP$_5$ (250 µM and (D) 6-PP-InsP5 (1 mM) (in syringe) with buffer (in cell), respectively. Raw heats per injection are shown in the top panel and the bottom panel represents the integrated heats of each injection. Note: The data shown represent the control runs corresponding to the ITC experiments of A and B.

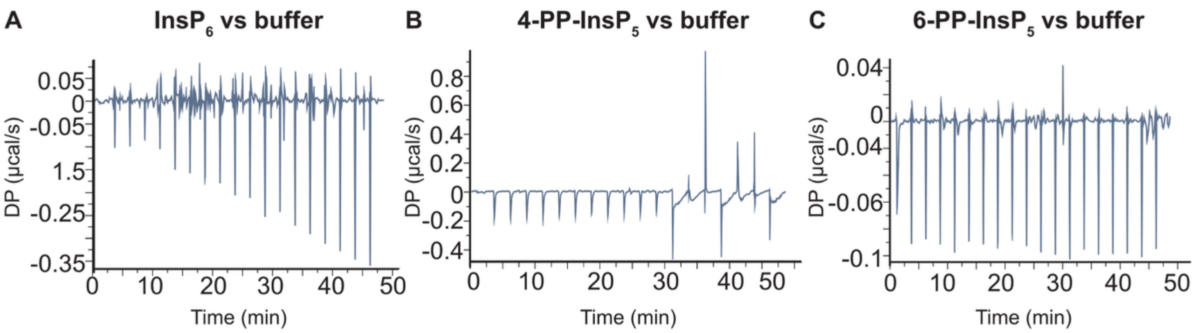

Figure S2: Isothermal titration calorimetry (ITC) assays of (A) $InsP_6$ (750 µM), (B) 4-PP-$InsP_5$ (1 mM) and (C) 6-PP-$InsP_5$ (500 µM) (in syringe) with buffer (in cell), respectively. Raw heats per injection are shown in the top panel and the bottom panel represents the integrated heats of each injection. Note: The data shown represent the control runs corresponding to the ITC experiments in Figure 7A–C.

6 References

[1] A. Hofer, G. S. Cremosnik, A. C. Müller, R. Giambruno, C. Trefzer, G. Superti-Furga, K. L. Bennett, H. J. Jessen, *Chem. Eur. J.*, **2015**, *21*, 10116–10122.

[2] S. Hostachy, T. Utesch, K. Franke, G. L. Dornan, D. Furkert, B. Türkaydin, V. Haucke, H. Sun, D. Fiedler, *Chem. Sci.*, **2021**, *12*, 10696–10702.

[3] M. Wu, L. S. Chong, D. H. Perlman, A. C. Resnick, D. Fiedler, *Proc. Natl. Acad. Sci. U.S.A.*, **2016**, *113*, E6757–E6765.

[4] J. Ma, J. Wehrle, D. Frank, L. Lorenzen, C. Popp, W. Driever, R. Grosse, H. J. Jessen, *Chem. Sci.*, **2024**, *15*, 6478–6487.

[5] D. Qiu, C. Gu, G. Liu, K. Ritter, V. B. Eisenbeis, T. Bittner, A. Gruzdev, L. Seidel, B. Bengsch, S. B. Shears, H. J. Jessen, *Chem. Sci.*, **2023**, *14*, 658–667.

[6] G. Baudin, B. I. Glänzer, K. S. Swaminathan, A. Vasella, *Helv. Chim. Acta*, **1988**, *71*, 1367–1378.

[7] C. Tantisuwanno, F. Dang, K. Bender, J. D. Spencer, M. E. Jennings, H. A. Barton, A. Joy, *Biomacromolecules*, **2021**, *22*, 2910–2920.

[8] R. Schneider, K. Lami, I. Prucker, S. C. Stolze, A. Strauß, J. M. Schmidt, S. M. Bartsch, K. Langenbach, E. Lange, K. Ritter, D. Furkert, N. Faiß, S. Kumar, M. Shamim Hasan, A. Makris, L. Krusenbaum, S. Wege, Y. Z. Belay, S. Kriescher, J. The, M. Harings, F. M. W. Grundler, M. K. Ried-Lasi, H. Schoof, P. Gaugler, M. Kamleitner, D. Fiedler, H. Nakagami, R. F. H. Giehl, T. Lahaye, S. Bhattacharjee, H. J. Jessen, V. Gaugler, G. Schaaf, *J. Integr. Plant Biol.*, **2025**, *67*, 3123–3151.

[9] D. Furkert, M. Nadler-Holly, D. Fiedler, *STAR Protocols*, **2021**, *2*, 100277.

[10] J. Rappsilber, Y. Ishihama, M. Mann, *Anal. Chem.*, **2003**, *75*, 663–670.

[11] J. Cox, M. Mann, *Nat. Biotechnol.*, **2008**, *26*, 1367–1372.

[12] S. Tyanova, T. Temu, P. Sinitcyn, A. Carlson, M. Y. Hein, T. Geiger, M. Mann, J. Cox, *Nat. Methods*, **2016**, *13*, 731–740.

[13] P. Rana, A. Edathil Kadangodan, P. Koley, R. Ghosh, N. J. Pullagurla, H. C. Naik, P. Mondal, S. Gayen, D. Laha, *Nat Chem Biol*, **2025**, DOI: 10.1038/s41589-025-01961-w.

7 Gene Ontology Term Enrichment Analysis by g:Profiler

Table 1: GO-Term Enrichment Results of the Combined Affi-Gel Root Dataset (g:Profiler Output).
The table summarizes the Affi-Gel root enrichment results, including both the on-bead and elution fractions.The results were obtained using g:Profiler.

Source	Term Name	Term ID	p_{adj}	$-log_{10}$ (p_{adj})	Term Size	Intersection Size
GO:MF	phospholipid binding	GO:0005543	0,00061188	3,213333878	135	7
GO:MF	phosphatidylinositol kinase activity	GO:0052742	0,002512644	2,599869077	30	4
GO:MF	1-phosphatidylinositol-4-phosphate 5-kinase activity	GO:0016308	0,003832301	2,416540376	11	3
GO:MF	inositol-1,4,5-trisphosphate 6-kinase activity	GO:0000823	0,005617333	2,250469797	2	2
GO:MF	inositol-1,4,5,6-tetrakisphosphate 3-kinase activity	GO:0000824	0,005617333	2,250469797	2	2
GO:MF	inositol-1,2,3,4,6-pentakisphosphate 5-kinase activity	GO:0102732	0,005617333	2,250469797	2	2
GO:MF	inositol-1,3,4,6-tetrakisphosphate 5-kinase activity	GO:0047326	0,005617333	2,250469797	2	2
GO:MF	lipid kinase activity	GO:0001727	0,009861894	2,006039655	42	4
GO:MF	lipid binding	GO:0008289	0,018509837	1,732597404	310	8
GO:MF	binding	GO:0005488	0,032995384	1,481546816	15005	82
GO:MF	5'-3' RNA exonuclease activity	GO:0004534	0,033513749	1,474776985	4	2
GO:BP	RNA processing	GO:0006396	7,63E-07	6,117268116	1086	22
GO:BP	mRNA metabolic process	GO:0016071	2,14704E-06	5,66815932	569	16
GO:BP	mRNA processing	GO:0006397	1,06837E-05	4,971278665	468	14
GO:BP	ribosome biogenesis	GO:0042254	0,000117291	3,930734377	254	10
GO:BP	nucleic acid metabolic process	GO:0090304	0,000213307	3,670994737	4239	40
GO:BP	RNA metabolic process	GO:0016070	0,000242699	3,614931285	3742	37
GO:BP	RNA biosynthetic process	GO:0032774	0,000710247	3,148590784	3396	34
GO:BP	nucleic acid biosynthetic process	GO:0141187	0,001183156	2,926958144	3471	34
GO:BP	rRNA processing	GO:0006364	0,001546271	2,810714302	194	8
GO:BP	rRNA metabolic process	GO:0016072	0,001869174	2,72835023	199	8
GO:BP	vesicle-mediated transport	GO:0016192	0,002278804	2,642293021	437	11
GO:BP	nucleobase-containing compound metabolic process	GO:0006139	0,002328473	2,632928784	4634	40
GO:BP	RNA splicing	GO:0008380	0,002456317	2,609715611	355	10
GO:BP	ribonucleoprotein complex biogenesis	GO:0022613	0,003141115	2,502916231	365	10
GO:BP	endocytosis	GO:0006897	0,003637191	2,439233844	57	5
GO:BP	nucleobase-containing compound biosynthetic process	GO:0034654	0,004957258	2,304758461	3696	34
GO:BP	clathrin-dependent endocytosis	GO:0072583	0,008195186	2,086441172	32	4
GO:BP	receptor-mediated endocytosis	GO:0006898	0,009294689	2,031765119	33	4
GO:BP	phosphatidylinositol metabolic process	GO:0046488	0,013204288	1,879284998	74	5
GO:BP	negative regulation of clathrin-dependent endocytosis	GO:1900186	0,014061189	1,85197795	2	2
GO:BP	negative regulation of receptor-mediated endocytosis	GO:0048261	0,014061189	1,85197795	2	2
GO:BP	negative regulation of endocytosis	GO:0045806	0,014061189	1,85197795	2	2
GO:BP	gene expression	GO:0010467	0,026040601	1,584348996	5292	41
GO:BP	import into cell	GO:0098657	0,027402432	1,562210895	86	5
GO:BP	mRNA splicing, via spliceosome	GO:0000398	0,040063977	1,397245945	223	7
GO:BP	regulation of receptor-mediated endocytosis	GO:0048259	0,042064323	1,376086094	3	2
GO:BP	regulation of clathrin-dependent endocytosis	GO:2000369	0,042064323	1,376086094	3	2
GO:CC	nucleus	GO:0005634	4,31E-10	9,36598358	5696	61
GO:CC	intracellular membrane-bounded organelle	GO:0043231	0,000561056	3,250993526	11808	79
GO:CC	membrane-bounded organelle	GO:0043227	0,00060015	3,221740532	11823	79
GO:CC	nuclear protein-containing complex	GO:0140513	0,002326996	2,63320443	651	13
GO:CC	intracellular anatomical structure	GO:0005622	0,004042179	2,39338444	13505	84
GO:CC	organelle lumen	GO:0043233	0,004122886	2,384798645	1245	18
GO:CC	intracellular organelle lumen	GO:0070013	0,004122886	2,384798645	1245	18
GO:CC	membrane-enclosed lumen	GO:0031974	0,004122886	2,384798645	1245	18
GO:CC	intracellular organelle	GO:0043229	0,004878838	2,311683608	12312	79
GO:CC	organelle	GO:0043226	0,005165761	2,286865705	12326	79
GO:CC	cell plate	GO:0009504	0,006486978	2,1879576	41	4
GO:CC	nuclear lumen	GO:0031981	0,006813004	2,16666136	1054	16
GO:CC	clathrin-coated vesicle	GO:0030136	0,039645122	1,401810244	65	4
KEGG	Inositol phosphate metabolism	KEGG:00562	1,64817E-05	4,782997511	76	7
KEGG	Ribosome biogenesis in eukaryotes	KEGG:03008	8,15701E-05	4,088469182	96	7
KEGG	Phosphatidylinositol signaling system	KEGG:04070	0,000246659	3,60790327	74	6
KEGG	Spliceosome	KEGG:03040	0,001518138	2,818688798	205	8
KEGG	Endocytosis	KEGG:04144	0,017318747	1,76148353	158	6

Table 2: GO-Term Enrichment Results of the Combined Affi-Gel Shoot Dataset (g:Profiler Output).
The table summarizes the Affi-Gel shoot enrichment results, including both the on-bead and elution fractions.The results were obtained using g:Profiler.

Source	*Term Name*	*Term ID*	p_{adj}	$-log_{10}(p_{adj})$	*Term Size*	*Intersection Size*
GO:MF	lipid binding	GO:0008289	1,13524E-05	4,944913233	310	11
GO:MF	phosphatidylinositol kinase activity	GO:0052742	3,52182E-05	4,453232499	30	5
GO:MF	lipid kinase activity	GO:0001727	0,00020265	3,693254243	42	5
GO:MF	binding	GO:0005488	0,000655415	3,183483876	15005	78
GO:MF	protein binding	GO:0005515	0,002910719	2,535999729	7655	50
GO:MF	1-phosphatidylinositol-4-phosphate 5-kinase activity	GO:0016308	0,003080756	2,511342695	11	3
GO:MF	phospholipid binding	GO:0005543	0,004928159	2,307315254	135	6
GO:MF	phosphatidylinositol binding	GO:0035091	0,005098986	2,292516198	80	5
GO:BP	phosphatidylinositol metabolic process	GO:0046488	1,28318E-05	4,8917124	74	7
GO:BP	vesicle-mediated transport	GO:0016192	0,000118903	3,924806722	437	12
GO:BP	phosphatidylinositol phosphate biosynthetic process	GO:0046854	0,00047896	3,31970079	18	4
GO:BP	glycerophospholipid metabolic process	GO:0006650	0,000810394	3,091303762	135	7
GO:BP	post-Golgi vesicle-mediated transport	GO:0006892	0,002672265	2,573120513	27	4
GO:BP	glycerolipid metabolic process	GO:0046486	0,004056063	2,391895323	172	7
GO:BP	RNA biosynthetic process	GO:0032774	0,008285983	2,081655941	3396	30
GO:BP	nucleic acid biosynthetic process	GO:0141187	0,012807843	1,892524013	3471	30
GO:BP	phosphatidylinositol biosynthetic process	GO:0006661	0,016278415	1,788387894	42	4
GO:BP	phospholipid metabolic process	GO:0006644	0,017259674	1,762967413	215	7
GO:BP	regulation of cellular process	GO:0050794	0,018150899	1,74110187	4692	36
GO:BP	nucleobase-containing compound biosynthetic process	GO:0034654	0,04337145	1,36279606	3696	30
GO:BP	regulation of biological process	GO:0050789	0,047079995	1,327163593	5102	37
GO:CC	clathrin-coated vesicle	GO:0030136	8,35013E-05	4,078306864	65	6
GO:CC	coated vesicle	GO:0030135	0,000995281	3,00205429	99	6
GO:CC	preribosome, large subunit precursor	GO:0030687	0,04305172	1,366009491	6	2
GO:CC	nucleus	GO:0005634	0,044952348	1,347247622	5696	43
KEGG	Endocytosis	KEGG:04144	1,82042E-06	5,73982948	158	9
KEGG	Inositol phosphate metabolism	KEGG:00562	2,53274E-06	5,596409471	76	7
KEGG	Phosphatidylinositol signaling system	KEGG:04070	0,001023764	2,989800318	74	5

Table 3: GO-Term Enrichment Results of the Combined Photoaffinity Root Dataset (g:Profiler Output).
The table summarizes the results from all photoaffinity shoot conditions, combining both the custom and commercial linker reagents as well as the different competition controls. The results were obtained using g:Profiler.

Source	*Term Name*	*Term ID*	p_{adj}	$-log_{10}(p_{adj})$	*Term Size*	*Intersection Size*
GO:MF	RNA binding	GO:0003723	2,02E-14	13,69512889	2517	114
GO:MF	transcription coregulator activity	GO:0003712	1,65E-11	10,78305308	108	20
GO:MF	protein-macromolecule adaptor activity	GO:0030674	1,70E-10	9,769365657	202	25
GO:MF	binding	GO:0005488	6,42E-09	8,192677769	15005	369
GO:MF	RNA helicase activity	GO:0003724	1,53E-08	7,81520633	92	16
GO:MF	ATP-dependent activity, acting on RNA	GO:0008186	1,53E-08	7,81520633	92	16
GO:MF	mRNA binding	GO:0003729	2,89E-08	7,538641065	1619	73
GO:MF	protein binding	GO:0005515	2,40021E-06	5,619749888	7655	214
GO:MF	helicase activity	GO:0004386	2,68991E-05	4,570262555	214	19
GO:MF	ATP-dependent activity	GO:0140657	4,59422E-05	4,337788245	858	42
GO:MF	organic cyclic compound binding	GO:0097159	4,75589E-05	4,322767878	8337	223
GO:MF	hydrolase activity, acting on acid anhydrides	GO:0016817	5,28932E-05	4,276600465	831	41
GO:MF	hydrolase activity, acting on acid anhydrides, in phosphorus-containing anhydrides	GO:0016818	0,000121754	3,914518533	826	40
GO:MF	ribonucleoside triphosphate phosphatase activity	GO:0017111	0,000239319	3,621023083	753	37
GO:MF	pyrophosphatase activity	GO:0016462	0,000244522	3,611682106	817	39
GO:MF	nucleic acid binding	GO:0003676	0,000464365	3,333140224	5097	147
GO:MF	ATP hydrolysis activity	GO:0016887	0,00107481	2,96866823	552	29
GO:MF	phosphatidylinositol kinase activity	GO:0052742	0,008322077	2,079768258	30	6
GO:MF	modified amino acid binding	GO:0072341	0,009708388	2,012852895	19	5
GO:MF	actin binding	GO:0003779	0,010637848	1,973146219	120	11
GO:MF	small molecule binding	GO:0036094	0,011458522	1,940871387	6758	177
GO:MF	ion binding	GO:0043167	0,013331164	1,875131916	6637	174
GO:MF	1-phosphatidylinositol-4-phosphate 5-kinase activity	GO:0016308	0,01547963	1,810239433	11	4
GO:MF	anion binding	GO:0043168	0,016576497	1,780507233	3218	96
GO:MF	carbohydrate derivative binding	GO:0097367	0,019448459	1,711114795	2856	87

GO:MF	phospholipase D activity	GO:0004630	0,022847593	1,641159541	12	4
GO:MF	N-acylphosphatidylethanolamine-specific phospholipase D activity	GO:0070290	0,022847593	1,641159541	12	4
GO:MF	nucleotide binding	GO:0000166	0,024196841	1,616241322	3124	93
GO:MF	isocitrate dehydrogenase (NAD+) activity	GO:0004449	0,025286834	1,597105538	5	3
GO:MF	nucleoside phosphate binding	GO:1901265	0,025969866	1,585530285	3130	93
GO:MF	purine ribonucleoside triphosphate binding	GO:0035639	0,027790238	1,556107727	2678	82
GO:MF	heterocyclic compound binding	GO:1901363	0,042814577	1,368408342	3258	95
GO:BP	mRNA metabolic process	GO:0016071	3,17E-32	31,49881886	569	69
GO:BP	RNA splicing	GO:0008380	1,00E-31	30,99984409	355	56
GO:BP	mRNA processing	GO:0006397	6,91E-31	30,16053375	468	62
GO:BP	RNA processing	GO:0006396	2,17E-28	27,66362373	1086	88
GO:BP	mRNA splicing, via spliceosome	GO:0000398	5,32E-20	19,27404242	223	36
GO:BP	RNA splicing, via transesterification reactions	GO:0000375	2,99E-19	18,52439684	250	37
GO:BP	RNA splicing, via transesterification reactions with bulged adenosine as nucleophile	GO:0000377	2,99E-19	18,52439684	250	37
GO:BP	nucleobase-containing compound biosynthetic process	GO:0034654	6,31E-15	14,19998472	3696	143
GO:BP	RNA biosynthetic process	GO:0032774	5,97E-14	13,22374447	3396	133
GO:BP	RNA metabolic process	GO:0016070	3,04E-13	12,516669	3742	140
GO:BP	nucleic acid biosynthetic process	GO:0141187	3,59E-13	12,44430754	3471	133
GO:BP	nucleobase-containing compound metabolic process	GO:0006139	6,72E-12	11,17281248	4634	158
GO:BP	nucleic acid metabolic process	GO:0090304	2,99E-10	9,524939431	4239	144
GO:BP	biosynthetic process	GO:0009058	1,73E-07	6,762698536	7320	203
GO:BP	gene expression	GO:0010467	3,09086E-06	5,509920284	5292	155
GO:BP	macromolecule biosynthetic process	GO:0009059	4,79102E-06	5,319571655	5736	164
GO:BP	ribonucleoprotein complex biogenesis	GO:0022613	9,00489E-06	5,045521383	365	26
GO:BP	response to temperature stimulus	GO:0009266	2,97416E-05	4,526635761	644	35
GO:BP	rRNA processing	GO:0006364	0,000877378	3,056813351	194	16
GO:BP	protein folding	GO:0006457	0,001101049	2,958193368	152	14
GO:BP	response to cold	GO:0009409	0,001174159	2,930272905	410	24
GO:BP	rRNA metabolic process	GO:0016072	0,001227632	2,910931686	199	16
GO:BP	negative regulation of biosynthetic process	GO:0009890	0,001305056	2,884370903	658	32
GO:BP	cellular component biogenesis	GO:0044085	0,001778875	2,749854539	1115	45
GO:BP	negative regulation of macromolecule biosynthetic process	GO:0010558	0,001872111	2,727668363	637	31
GO:BP	negative regulation of macromolecule metabolic process	GO:0010605	0,00293305	2,532680538	684	32
GO:BP	organophosphate metabolic process	GO:0019637	0,003050961	2,515563354	620	30
GO:BP	protein-RNA complex assembly	GO:0022618	0,003103596	2,508134809	122	12
GO:BP	endoplasmic reticulum tubular network organization	GO:0071786	0,003255647	2,487362626	7	4
GO:BP	protein-RNA complex organization	GO:0071826	0,003678064	2,43438071	124	12
GO:BP	negative regulation of metabolic process	GO:0009892	0,004770901	2,321399583	734	33
GO:BP	miRNA processing	GO:0035196	0,005885259	2,230234407	26	6
GO:BP	primary metabolic process	GO:0044238	0,006921356	2,159808782	10896	254
GO:BP	ribosome biogenesis	GO:0042254	0,00699659	2,155113589	254	17
GO:BP	glycerophospholipid metabolic process	GO:0006650	0,008830282	2,054025423	135	12
GO:BP	response to chemical	GO:0042221	0,010235903	1,989873845	2940	88
GO:BP	response to stimulus	GO:0050896	0,011708611	1,931494604	6002	155
GO:BP	cellular process	GO:0009987	0,014707193	1,832470216	16600	355
GO:BP	glycerolipid metabolic process	GO:0046486	0,02207413	1,65611641	172	13
GO:BP	nuclear-transcribed mRNA catabolic process, nonsense-mediated decay	GO:0000184	0,022710923	1,643765216	20	5
GO:BP	regulation of RNA splicing	GO:0043484	0,037367565	1,427505196	22	5
GO:BP	endoplasmic reticulum organization	GO:0007029	0,037367565	1,427505196	22	5
GO:BP	phosphatidylcholine metabolic process	GO:0046470	0,046994638	1,327951688	23	5
GO:CC	intracellular anatomical structure	GO:0005622	3,61E-30	29,44254694	13505	414
GO:CC	nuclear protein-containing complex	GO:0140513	5,44E-28	27,2643184	651	73
GO:CC	spliceosomal complex	GO:0005681	5,99E-27	26,22233118	130	36
GO:CC	intracellular organelle	GO:0043229	2,06E-23	22,68603522	12312	382
GO:CC	organelle	GO:0043226	2,80E-23	22,55337807	12326	382
GO:CC	intracellular membrane-bounded organelle	GO:0043231	3,66E-22	21,43637355	11808	370
GO:CC	membrane-bounded organelle	GO:0043227	5,04E-22	21,29781857	11823	370
GO:CC	mediator complex	GO:0016592	6,11E-20	19,21417321	49	21
GO:CC	nuclear speck	GO:0016607	2,01E-19	18,69748867	83	25
GO:CC	organelle lumen	GO:0043233	1,55E-18	17,81023149	1245	86
GO:CC	intracellular organelle lumen	GO:0070013	1,55E-18	17,81023149	1245	86
GO:CC	membrane-enclosed lumen	GO:0031974	1,55E-18	17,81023149	1245	86
GO:CC	nuclear lumen	GO:0031981	8,78E-18	17,05629516	1054	77
GO:CC	nuclear body	GO:0016604	1,74E-17	16,75853551	119	27

GO:CC	nucleoplasm	GO:0005654	4,97E-17	16,30393192	357	43
GO:CC	nucleus	GO:0005634	1,25E-16	15,90309291	5696	218
GO:CC	cytosol	GO:0005829	1,22E-11	10,91447295	2420	112
GO:CC	protein-containing complex	GO:0032991	2,19E-11	10,65882475	2604	117
GO:CC	cytoplasm	GO:0005737	6,16E-09	8,210168928	9524	286
GO:CC	cellular anatomical structure	GO:0110165	1,13E-08	7,945883095	18498	452
GO:CC	nucleolus	GO:0005730	5,90E-08	7,228940902	701	45
GO:CC	ribonucleoprotein complex	GO:1990904	4,86E-07	6,313104503	831	48
GO:CC	cell periphery	GO:0071944	0,000200638	3,697586241	2917	104
GO:CC	plasma membrane	GO:0005886	0,000212532	3,672575208	2524	93
GO:CC	plasmodesma	GO:0009506	0,000235819	3,627421307	900	44
GO:CC	cell-cell junction	GO:0005911	0,000235819	3,627421307	900	44
GO:CC	cell junction	GO:0030054	0,000235819	3,627421307	900	44
GO:CC	anchoring junction	GO:0070161	0,000235819	3,627421307	900	44
GO:CC	symplast	GO:0055044	0,000242777	3,614792872	901	44
GO:CC	endoplasmic reticulum lumen	GO:0005788	0,003982869	2,399803965	40	7
GO:CC	nuclear inner membrane	GO:0005637	0,006714494	2,172986727	10	4
GO:CC	membraneless organelle	GO:0043228	0,007100135	2,148733369	2568	88
GO:CC	intracellular membraneless organelle	GO:0043232	0,007100135	2,148733369	2568	88
GO:CC	cytoplasmic side of endoplasmic reticulum membrane	GO:0098554	0,015204319	1,818033033	5	3
KEGG	Spliceosome	KEGG:03040	4,60E-30	29,33749708	205	52
KEGG	mRNA surveillance pathway	KEGG:03015	6,65509E-06	5,176846028	119	18
KEGG	Phosphatidylinositol signaling system	KEGG:04070	0,048016644	1,318608199	74	9
KEGG	Nucleocytoplasmic transport	KEGG:03013	0,049714598	1,303516071	105	11
WP	TCA cycle Krebs cycle	WP:WP2624	0,012633814	1,898465536	29	4

Table 4: GO-Term Enrichment Results of the Combined Photoaffinity Shoot Dataset (g:Profiler Output). The table summarizes the results from all photoaffinity shoot conditions, combining both the custom and commercial linker reagents as well as the different competition controls. The results were obtained using g:Profiler.

Source	Term Name	Term ID	p_{adj}	$-\log_{10}(p_{adj})$	Term Size	Intersection Size
GO:MF	catalytic activity	GO:0003824	3,20E-23	22,49543984	9210	362
GO:MF	oxidoreductase activity	GO:0016491	6,69E-13	12,17477969	1475	92
GO:MF	lyase activity	GO:0016829	6,65E-10	9,177256729	446	41
GO:MF	carbon-carbon lyase activity	GO:0016830	3,10E-08	7,508720875	114	19
GO:MF	intramolecular oxidoreductase activity	GO:0016860	1,15E-07	6,939051248	51	13
GO:MF	isomerase activity	GO:0016853	6,62E-07	6,179080343	270	27
GO:MF	small molecule binding	GO:0036094	1,01653E-06	5,992879808	6758	240
GO:MF	oxidoreductase activity, acting on the CH-OH group of donors, NAD or NADP as acceptor	GO:0016616	2,86614E-06	5,542703185	148	19
GO:MF	modified amino acid binding	GO:0072341	3,32693E-06	5,477955742	19	8
GO:MF	ligase activity, forming carbon-carbon bonds	GO:0016885	6,83533E-06	5,165240779	9	6
GO:MF	CoA carboxylase activity	GO:0016421	6,83533E-06	5,165240779	9	6
GO:MF	transferase activity, transferring alkyl or aryl (other than methyl) groups	GO:0016765	8,46267E-06	5,072492828	158	19
GO:MF	copper ion binding	GO:0005507	8,58318E-06	5,066351883	192	21
GO:MF	oxidoreductase activity, acting on CH-OH group of donors	GO:0016614	2,66354E-05	4,574541365	187	20
GO:MF	ligase activity	GO:0016874	3,04256E-05	4,516760938	283	25
GO:MF	magnesium ion binding	GO:0000287	4,36546E-05	4,359969567	141	17
GO:MF	carboxylic acid binding	GO:0031406	5,1285E-05	4,290009857	96	14
GO:MF	organic acid binding	GO:0043177	5,1285E-05	4,290009857	96	14
GO:MF	hydro-lyase activity	GO:0016836	5,18682E-05	4,285098767	82	13
GO:MF	cation binding	GO:0043169	5,91847E-05	4,227790313	3961	151
GO:MF	metal ion binding	GO:0046872	8,14554E-05	4,08907998	3948	150
GO:MF	carboxy-lyase activity	GO:0016831	0,0001199	3,921181338	74	12
GO:MF	ion binding	GO:0043167	0,000166943	3,777432604	6637	226
GO:MF	amide binding	GO:0033218	0,000344326	3,46303037	32	8
GO:MF	alkali metal ion binding	GO:0031420	0,000558352	3,253091932	16	6
GO:MF	cobalt ion binding	GO:0050897	0,000817688	3,087412585	47	9
GO:MF	glutathione transferase activity	GO:0004364	0,000863537	3,063719232	60	10
GO:MF	monocarboxylic acid binding	GO:0033293	0,001009251	2,996001009	61	10
GO:MF	oxidoreductase activity, acting on the aldehyde or oxo group of donors	GO:0016903	0,001009251	2,996001009	61	10
GO:MF	salicylic acid binding	GO:1901149	0,001639619	2,78525705	28	7
GO:MF	oligopeptide binding	GO:1900750	0,002316402	2,635186057	12	5
GO:MF	glutathione binding	GO:0043295	0,002316402	2,635186057	12	5

GO:MF	vitamin binding	GO:0019842	0,002739842	2,562274449	133	14
GO:MF	aldehyde dehydrogenase (NAD+) activity	GO:0004029	0,00368426	2,433649739	13	5
GO:MF	aldehyde dehydrogenase [NAD(P)+] activity	GO:0004030	0,00368426	2,433649739	13	5
GO:MF	acetyl-CoA carboxylase activity	GO:0003989	0,004365374	2,35997854	7	4
GO:MF	potassium ion binding	GO:0030955	0,005609504	2,251075539	14	5
GO:MF	pyruvate kinase activity	GO:0004743	0,005609504	2,251075539	14	5
GO:MF	ligase activity, forming carbon-nitrogen bonds	GO:0016879	0,007551507	2,121966385	61	9
GO:MF	protein domain specific binding	GO:0019904	0,012403055	1,906471327	97	11
GO:MF	heterocyclic compound binding	GO:1901363	0,012675717	1,897027466	3258	119
GO:MF	NAD binding	GO:0051287	0,01325226	1,877710054	81	10
GO:MF	binding	GO:0005488	0,015069233	1,821908862	15005	431
GO:MF	intramolecular oxidoreductase activity, interconverting aldoses and ketoses	GO:0016861	0,016259654	1,788888697	17	5
GO:MF	NADPH binding	GO:0070402	0,020288671	1,6927464	4	3
GO:MF	oxidoreductase activity, acting on the aldehyde or oxo group of donors, NAD or NADP as acceptor	GO:0016620	0,027324711	1,563444418	42	7
GO:MF	anion binding	GO:0043168	0,028361904	1,547264616	3218	116
GO:MF	carbon-oxygen lyase activity	GO:0016835	0,03043674	1,516601863	165	14
GO:MF	sulfur compound binding	GO:1901681	0,030440997	1,516541133	30	6
GO:MF	oxidoreductase activity, acting on single donors with incorporation of molecular oxygen, incorporation of one atom of oxygen (internal monooxygenases or internal mixed function oxidases)	GO:0016703	0,049746736	1,303235407	5	3
GO:MF	oxidoreductase activity, acting on metal ions, oxygen as acceptor	GO:0016724	0,049746736	1,303235407	5	3
GO:MF	ferroxidase activity	GO:0004322	0,049746736	1,303235407	5	3
GO:BP	small molecule metabolic process	GO:0044281	2,07E-52	51,68311778	1602	161
GO:BP	carboxylic acid metabolic process	GO:0019752	7,21E-38	37,14189713	955	108
GO:BP	oxoacid metabolic process	GO:0043436	4,75E-37	36,32355251	1067	113
GO:BP	organic acid metabolic process	GO:0006082	5,20E-37	36,28413547	1068	113
GO:BP	small molecule biosynthetic process	GO:0044283	9,04E-26	25,04384437	686	77
GO:BP	organophosphate metabolic process	GO:0019637	4,38E-24	23,35878749	620	71
GO:BP	amino acid metabolic process	GO:0006520	1,90E-23	22,72165849	435	59
GO:BP	nucleoside phosphate metabolic process	GO:0006753	4,39E-23	22,35710247	366	54
GO:BP	nucleobase-containing small molecule metabolic process	GO:0055086	7,27E-21	20,13825143	406	54
GO:BP	proteinogenic amino acid metabolic process	GO:0170039	2,99E-19	18,52375459	271	43
GO:BP	L-amino acid metabolic process	GO:0170033	4,03E-19	18,39473372	273	43
GO:BP	alpha-amino acid metabolic process	GO:1901605	4,60E-19	18,33695671	317	46
GO:BP	carboxylic acid biosynthetic process	GO:0046394	5,91E-18	17,22843559	556	59
GO:BP	organic acid biosynthetic process	GO:0016053	5,91E-18	17,22843559	556	59
GO:BP	cellular process	GO:0009987	1,83E-16	15,73689573	16600	500
GO:BP	nucleotide metabolic process	GO:0009117	1,95E-16	15,70924339	274	40
GO:BP	metabolic process	GO:0008152	4,81E-16	15,31754934	12629	417
GO:BP	ribonucleotide metabolic process	GO:0009259	5,71E-16	15,2434157	185	33
GO:BP	ribose phosphate metabolic process	GO:0019693	5,71E-16	15,2434157	185	33
GO:BP	carbohydrate derivative metabolic process	GO:1901135	4,99E-15	14,30220571	659	60
GO:BP	purine-containing compound metabolic process	GO:0072521	4,29E-14	13,36718297	271	37
GO:BP	generation of precursor metabolites and energy	GO:0006091	8,54E-14	13,06870547	464	48
GO:BP	organophosphate biosynthetic process	GO:0090407	1,14E-13	12,94134228	413	45
GO:BP	amino acid biosynthetic process	GO:0008652	1,28E-13	12,89437219	220	33
GO:BP	monocarboxylic acid metabolic process	GO:0032787	1,46E-13	12,8349791	489	49
GO:BP	nucleoside phosphate biosynthetic process	GO:1901293	1,93E-13	12,71519111	223	33
GO:BP	alpha-amino acid biosynthetic process	GO:1901607	2,00E-13	12,69849906	195	31
GO:BP	purine nucleotide metabolic process	GO:0006163	1,94E-12	11,71220149	211	31
GO:BP	proteinogenic amino acid biosynthetic process	GO:0170038	2,75E-12	11,56139819	158	27
GO:BP	L-amino acid biosynthetic process	GO:0170034	2,75E-12	11,56139819	158	27
GO:BP	purine ribonucleotide metabolic process	GO:0009150	6,11E-12	11,2136897	163	27
GO:BP	photosynthesis	GO:0015979	3,10E-11	10,50826833	281	34
GO:BP	nucleoside diphosphate metabolic process	GO:0009132	4,21E-11	10,37550694	78	19
GO:BP	nucleoside triphosphate metabolic process	GO:0009141	4,55E-11	10,34233055	136	24
GO:BP	nucleoside phosphate catabolic process	GO:1901292	6,94E-11	10,15858835	80	19
GO:BP	nucleoside diphosphate catabolic process	GO:0009134	1,55E-10	9,808396109	73	18
GO:BP	ribonucleoside triphosphate metabolic process	GO:0009199	1,71E-10	9,766910812	131	23
GO:BP	pyridine-containing compound catabolic process	GO:0072526	2,01E-10	9,697498488	74	18
GO:BP	pyruvate metabolic process	GO:0006090	2,58E-10	9,588457826	75	18
GO:BP	sulfur compound metabolic process	GO:0006790	2,68E-10	9,571868421	320	35
GO:BP	ribonucleoside diphosphate metabolic process	GO:0009185	3,30E-10	9,481217647	76	18

GO:BP	organophosphate catabolic process	GO:0046434	8,40E-10	9,075557247	91	19
GO:BP	purine ribonucleoside diphosphate catabolic process	GO:0009181	1,57E-09	8,803971232	72	17
GO:BP	ribonucleotide catabolic process	GO:0009261	1,57E-09	8,803971232	72	17
GO:BP	purine nucleoside diphosphate catabolic process	GO:0009137	1,57E-09	8,803971232	72	17
GO:BP	glycolytic process	GO:0006096	1,57E-09	8,803971232	72	17
GO:BP	ADP metabolic process	GO:0046031	1,57E-09	8,803971232	72	17
GO:BP	ADP catabolic process	GO:0046032	1,57E-09	8,803971232	72	17
GO:BP	ribonucleoside diphosphate catabolic process	GO:0009191	1,57E-09	8,803971232	72	17
GO:BP	purine ribonucleotide catabolic process	GO:0009154	1,57E-09	8,803971232	72	17
GO:BP	purine nucleotide catabolic process	GO:0006195	2,00E-09	8,698964776	73	17
GO:BP	pyridine nucleotide catabolic process	GO:0019364	2,00E-09	8,698964776	73	17
GO:BP	pyridine-containing compound metabolic process	GO:0072524	2,33E-09	8,631809349	134	22
GO:BP	nicotinamide nucleotide metabolic process	GO:0046496	2,41E-09	8,617823832	121	21
GO:BP	purine ribonucleoside diphosphate metabolic process	GO:0009179	3,20E-09	8,49423014	75	17
GO:BP	purine nucleoside diphosphate metabolic process	GO:0009135	3,20E-09	8,49423014	75	17
GO:BP	pyridine nucleotide metabolic process	GO:0019362	3,35E-09	8,474822457	123	21
GO:BP	nucleotide catabolic process	GO:0009166	5,06E-09	8,296182896	77	17
GO:BP	biosynthetic process	GO:0009058	5,16E-09	8,286987181	7320	261
GO:BP	ATP metabolic process	GO:0046034	5,46E-09	8,262619728	113	20
GO:BP	purine nucleoside triphosphate metabolic process	GO:0009144	1,47E-08	7,832670964	119	20
GO:BP	purine ribonucleoside triphosphate metabolic process	GO:0009205	1,47E-08	7,832670964	119	20
GO:BP	response to cadmium ion	GO:0046686	1,52E-08	7,818935482	71	16
GO:BP	carbohydrate derivative catabolic process	GO:1901136	2,45E-08	7,611318933	136	21
GO:BP	purine-containing compound catabolic process	GO:0072523	3,33E-08	7,478050532	86	17
GO:BP	cellular catabolic process	GO:0044248	3,40E-08	7,468958936	199	25
GO:BP	catabolic process	GO:0009056	7,75E-08	7,110566135	1379	76
GO:BP	pyruvate family amino acid metabolic process	GO:0009078	1,01E-07	6,996412388	31	11
GO:BP	nucleobase-containing compound catabolic process	GO:0034655	1,22E-07	6,912194833	228	26
GO:BP	purine-containing compound biosynthetic process	GO:0072522	5,61E-07	6,250928536	130	19
GO:BP	response to metal ion	GO:0010038	1,40331E-06	5,852845239	219	24
GO:BP	sulfur compound biosynthetic process	GO:0044272	2,85801E-06	5,543935758	175	21
GO:BP	ribonucleoside monophosphate biosynthetic process	GO:0009156	6,78611E-06	5,168379161	44	11
GO:BP	carbohydrate derivative biosynthetic process	GO:1901137	1,2391E-05	4,906892533	407	32
GO:BP	response to cold	GO:0009409	1,47461E-05	4,831322648	410	32
GO:BP	ribonucleoside monophosphate metabolic process	GO:0009161	1,8249E-05	4,738761464	48	11
GO:BP	response to abiotic stimulus	GO:0009628	2,02636E-05	4,693282773	2131	95
GO:BP	ribonucleotide biosynthetic process	GO:0009260	2,12935E-05	4,671753	99	15
GO:BP	ribose phosphate biosynthetic process	GO:0046390	2,12935E-05	4,671753	99	15
GO:BP	nucleotide biosynthetic process	GO:0009165	2,19852E-05	4,657869831	145	18
GO:BP	dicarboxylic acid metabolic process	GO:0043648	2,82172E-05	4,549486798	87	14
GO:BP	fatty acid metabolic process	GO:0006631	3,1262E-05	4,504983019	276	25
GO:BP	nucleoside monophosphate biosynthetic process	GO:0009124	3,59212E-05	4,444649009	51	11
GO:BP	L-leucine metabolic process	GO:0006551	4,32496E-05	4,364018021	23	8
GO:BP	alpha-amino acid catabolic process	GO:1901606	5,54534E-05	4,256071777	65	12
GO:BP	aromatic amino acid metabolic process	GO:0009072	5,85645E-05	4,232365631	92	14
GO:BP	L-amino acid catabolic process	GO:0170035	6,88677E-05	4,161984297	43	10
GO:BP	proteinogenic amino acid catabolic process	GO:0170040	6,88677E-05	4,161984297	43	10
GO:BP	nucleoside monophosphate metabolic process	GO:0009123	0,000110738	3,955703575	69	12
GO:BP	response to temperature stimulus	GO:0009266	0,00013894	3,857173226	644	40
GO:BP	lipid metabolic process	GO:0006629	0,000161243	3,792518845	1098	57
GO:BP	carbon fixation	GO:0015977	0,000179154	3,746773471	27	8
GO:BP	amino acid catabolic process	GO:0009063	0,000180188	3,744274813	72	12
GO:BP	branched-chain amino acid metabolic process	GO:0009081	0,000209786	3,678222655	48	10
GO:BP	mRNA metabolic process	GO:0016071	0,000375459	3,42543732	569	36
GO:BP	photosynthesis, dark reaction	GO:0019685	0,00042311	3,37354708	21	7
GO:BP	reductive pentose-phosphate cycle	GO:0019253	0,00042311	3,37354708	21	7
GO:BP	glutamine family amino acid biosynthetic process	GO:0009084	0,000441847	3,354727864	30	8
GO:BP	aromatic amino acid family biosynthetic process	GO:0009073	0,000575656	3,239837114	66	11
GO:BP	glutamine family amino acid metabolic process	GO:0009064	0,000605074	3,218191401	95	13
GO:BP	cold acclimation	GO:0009631	0,000800756	3,096499874	55	10
GO:BP	primary metabolic process	GO:0044238	0,000875541	3,057723411	10896	330
GO:BP	acyl-CoA biosynthetic process	GO:0071616	0,001179221	2,928404772	24	7
GO:BP	thioester biosynthetic process	GO:0035384	0,001179221	2,928404772	24	7
GO:BP	monocarboxylic acid biosynthetic process	GO:0072330	0,00122317	2,912513235	249	21
GO:BP	fatty acid biosynthetic process	GO:0006633	0,001306923	2,883750056	171	17
GO:BP	pyruvate family amino acid catabolic process	GO:0009080	0,001801555	2,744352408	10	5
GO:BP	response to stimulus	GO:0050896	0,002290901	2,639993738	6002	200

GO:BP	small molecule catabolic process	GO:0044282	0,002453104	2,610283955	179	17
GO:BP	photosynthesis, light harvesting	GO:0009765	0,002495144	2,602904423	37	8
GO:BP	mRNA processing	GO:0006397	0,002631742	2,579756737	468	30
GO:BP	L-histidine metabolic process	GO:0006547	0,003234271	2,490223627	11	5
GO:BP	imidazole-containing compound metabolic process	GO:0052803	0,003234271	2,490223627	11	5
GO:BP	L-histidine biosynthetic process	GO:0000105	0,003234271	2,490223627	11	5
GO:BP	lipid biosynthetic process	GO:0008610	0,003920353	2,406674807	630	36
GO:BP	L-arginine biosynthetic process	GO:0006526	0,005429406	2,265247699	12	5
GO:BP	UMP metabolic process	GO:0046049	0,005429406	2,265247699	12	5
GO:BP	pyrimidine ribonucleoside monophosphate biosynthetic process	GO:0009174	0,005429406	2,265247699	12	5
GO:BP	UMP biosynthetic process	GO:0006222	0,005429406	2,265247699	12	5
GO:BP	pyruvate family amino acid biosynthetic process	GO:0009079	0,005688191	2,245025866	20	6
GO:BP	phosphate-containing compound metabolic process	GO:0006796	0,005865341	2,231706759	2032	83
GO:BP	phosphorus metabolic process	GO:0006793	0,00619887	2,207687446	2035	83
GO:BP	amide metabolic process	GO:0043603	0,007974229	2,098311294	156	15
GO:BP	pyrimidine ribonucleoside monophosphate metabolic process	GO:0009173	0,008639802	2,063496208	13	5
GO:BP	response to chemical	GO:0042221	0,008758966	2,057547162	2940	110
GO:BP	response to oxidative stress	GO:0006979	0,009946025	2,002350468	426	27
GO:BP	endoplasmic reticulum tubular network organization	GO:0071786	0,010449825	1,980890992	7	4
GO:BP	RNA splicing	GO:0008380	0,01051723	1,97809864	355	24
GO:BP	cellular metabolic compound salvage	GO:0043094	0,011332415	1,945677539	73	10
GO:BP	protein peptidyl-prolyl isomerization	GO:0000413	0,011954302	1,922475761	33	7
GO:BP	toxin catabolic process	GO:0009407	0,01381589	1,85962112	46	8
GO:BP	toxin metabolic process	GO:0009404	0,014400149	1,841633025	60	9
GO:BP	response to toxic substance	GO:0009636	0,015266038	1,816273656	316	22
GO:BP	regulation of fatty acid metabolic process	GO:0019217	0,018127187	1,74166958	24	6
GO:BP	carboxylic acid catabolic process	GO:0046395	0,018729203	1,727480709	129	13
GO:BP	organic acid catabolic process	GO:0016054	0,018729203	1,727480709	129	13
GO:BP	L-leucine biosynthetic process	GO:0009098	0,019332728	1,713706863	15	5
GO:BP	detoxification	GO:0098754	0,019466849	1,710704333	275	20
GO:BP	pyrimidine ribonucleotide biosynthetic process	GO:0009220	0,023345566	1,631795591	25	6
GO:BP	branched-chain amino acid biosynthetic process	GO:0009082	0,023345566	1,631795591	25	6
GO:BP	pyrimidine ribonucleotide metabolic process	GO:0009218	0,023345566	1,631795591	25	6
GO:BP	response to stress	GO:0006950	0,024699308	1,60731522	3733	131
GO:BP	pyrimidine nucleoside monophosphate biosynthetic process	GO:0009130	0,02753816	1,560065079	16	5
GO:BP	monosaccharide biosynthetic process	GO:0046364	0,030187824	1,520168193	51	8
GO:BP	thioester metabolic process	GO:0035383	0,031664594	1,499426077	38	7
GO:BP	acyl-CoA metabolic process	GO:0006637	0,031664594	1,499426077	38	7
GO:BP	pyrimidine nucleoside monophosphate metabolic process	GO:0009129	0,03820521	1,417877414	17	5
GO:BP	IMP metabolic process	GO:0046040	0,03820521	1,417877414	17	5
GO:BP	IMP biosynthetic process	GO:0006188	0,03820521	1,417877414	17	5
GO:BP	nucleotide salvage	GO:0043173	0,03820521	1,417877414	17	5
GO:BP	water-soluble vitamin metabolic process	GO:0006767	0,039317825	1,40541051	84	10
GO:BP	purine ribonucleoside monophosphate biosynthetic process	GO:0009168	0,046571057	1,331883907	28	6
GO:BP	purine nucleoside monophosphate biosynthetic process	GO:0009127	0,046571057	1,331883907	28	6
GO:BP	negative regulation of fatty acid metabolic process	GO:0045922	0,049648448	1,304094323	4	3
GO:CC	cytoplasm	GO:0005737	3,02E-76	75,52014394	9524	509
GO:CC	plastid	GO:0009536	8,93E-72	71,04914536	2856	269
GO:CC	cytosol	GO:0005829	4,58E-65	64,33877588	2420	238
GO:CC	intracellular anatomical structure	GO:0005622	5,29E-60	59,27639559	13505	576
GO:CC	chloroplast	GO:0009507	7,70E-60	59,11333744	2351	227
GO:CC	plastid stroma	GO:0009532	9,64E-57	56,01572331	769	128
GO:CC	chloroplast stroma	GO:0009570	2,51E-56	55,59946345	762	127
GO:CC	intracellular membrane-bounded organelle	GO:0043231	1,58E-36	35,80207077	11808	506
GO:CC	membrane-bounded organelle	GO:0043227	2,52E-36	35,59860905	11823	506
GO:CC	intracellular organelle	GO:0043229	4,46E-33	32,35050321	12312	512
GO:CC	organelle	GO:0043226	6,78E-33	32,16883901	12326	512
GO:CC	plastid envelope	GO:0009526	3,18E-17	16,49716585	988	87
GO:CC	thylakoid	GO:0009579	7,34E-16	15,13430208	596	63
GO:CC	organelle envelope	GO:0031967	2,18E-15	14,66217994	1451	106
GO:CC	chloroplast envelope	GO:0009941	3,45E-12	11,46266112	692	62
GO:CC	cellular anatomical structure	GO:0110165	8,97E-11	10,04741613	18498	601
GO:CC	chloroplast thylakoid	GO:0009534	9,75E-10	9,011144542	517	48
GO:CC	plastid thylakoid	GO:0031976	1,12E-09	8,951371932	519	48
GO:CC	photosynthetic membrane	GO:0034357	3,88E-08	7,410662222	443	41

GO:CC	thylakoid membrane	GO:0042651	1,25E-07	6,90299782	442	40
GO:CC	chloroplast thylakoid membrane	GO:0009535	1,28E-07	6,892356004	424	39
GO:CC	plastid thylakoid membrane	GO:0055035	1,47E-07	6,833577396	426	39
GO:CC	outer membrane	GO:0019867	6,59E-07	6,181376751	546	44
GO:CC	organelle outer membrane	GO:0031968	6,59E-07	6,181376751	546	44
GO:CC	mitochondrion	GO:0005739	1,07844E-06	5,967205457	1720	95
GO:CC	spliceosomal complex	GO:0005681	2,13685E-06	5,67022695	130	19
GO:CC	nuclear speck	GO:0016607	2,75889E-05	4,559266003	83	14
GO:CC	plastid membrane	GO:0042170	4,30347E-05	4,366181358	674	46
GO:CC	cytoplasmic side of endoplasmic reticulum membrane	GO:0098554	0,000598528	3,222915799	5	4
GO:CC	thylakoid lumen	GO:0031977	0,001998524	2,699290713	74	11
GO:CC	nuclear body	GO:0016604	0,002260377	2,645819171	119	14
GO:CC	apoplast	GO:0048046	0,002630933	2,579890208	489	33
GO:CC	endoplasmic reticulum tubular network	GO:0071782	0,003989917	2,399036139	7	4
GO:CC	photosystem	GO:0009521	0,005484825	2,260837252	97	12
GO:CC	protein-containing complex	GO:0032991	0,007224396	2,141198457	2604	113
GO:CC	secretory vesicle	GO:0099503	0,009406675	2,026563858	170	16
GO:CC	plastoglobule	GO:0010287	0,01461109	1,83531738	62	9
GO:CC	photosystem II	GO:0009523	0,017800489	1,749568071	78	10
GO:CC	plastid thylakoid lumen	GO:0031978	0,020303928	1,692419946	51	8
GO:CC	chloroplast thylakoid lumen	GO:0009543	0,020303928	1,692419946	51	8
GO:CC	intracellular membraneless organelle	GO:0043232	0,024358978	1,613340936	2568	109
GO:CC	membraneless organelle	GO:0043228	0,024358978	1,613340936	2568	109
GO:CC	vacuole	GO:0005773	0,025744319	1,589318588	1094	55
GO:CC	membrane protein complex	GO:0098796	0,046549609	1,332083965	452	28
KEGG	Metabolic pathways	KEGG:01100	1,02E-16	15,98999378	2337	214
KEGG	Biosynthesis of amino acids	KEGG:01230	1,28E-12	11,89318629	242	48
KEGG	Carbon metabolism	KEGG:01200	6,96E-12	11,15770204	271	50
KEGG	Biosynthesis of secondary metabolites	KEGG:01110	1,27E-09	8,895242546	1283	128
KEGG	Pyruvate metabolism	KEGG:00620	1,19406E-06	5,922974751	96	22
KEGG	Carbon fixation in photosynthetic organisms	KEGG:00710	2,13441E-06	5,67072158	68	18
KEGG	Glycolysis / Gluconeogenesis	KEGG:00010	3,83241E-06	5,416528233	119	24
KEGG	2-Oxocarboxylic acid metabolism	KEGG:01210	8,94954E-05	4,048199272	103	20
KEGG	Arginine biosynthesis	KEGG:00220	0,001427539	2,845412053	36	10
KEGG	Histidine metabolism	KEGG:00340	0,003106431	2,50773825	19	7
KEGG	Glyoxylate and dicarboxylate metabolism	KEGG:00630	0,006735476	2,171631732	77	14
KEGG	Alanine, aspartate and glutamate metabolism	KEGG:00250	0,00721844	2,141556666	51	11
KEGG	Glutathione metabolism	KEGG:00480	0,015565539	1,807835824	103	16
KEGG	C5-Branched dibasic acid metabolism	KEGG:00660	0,017246295	1,763304181	7	4
KEGG	Spliceosome	KEGG:03040	0,019976165	1,699487893	205	25
KEGG	Fructose and mannose metabolism	KEGG:00051	0,049083772	1,309062072	63	11
KEGG	Valine, leucine and isoleucine biosynthesis	KEGG:00290	0,049652206	1,304061455	21	6
WP	Glycolysis	WP:WP2621	0,002925614	2,533783035	37	10
WP	Glucosinolate biosynthesis from methionine	WP:WP4597	0,036664787	1,435750836	15	5

8 NMR – Spectra

(Sorted according to molecule numbering)

Compound 8: ^{1}H – NMR ($CDCl_3$, 400 MHz)

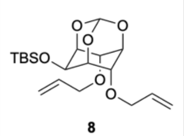

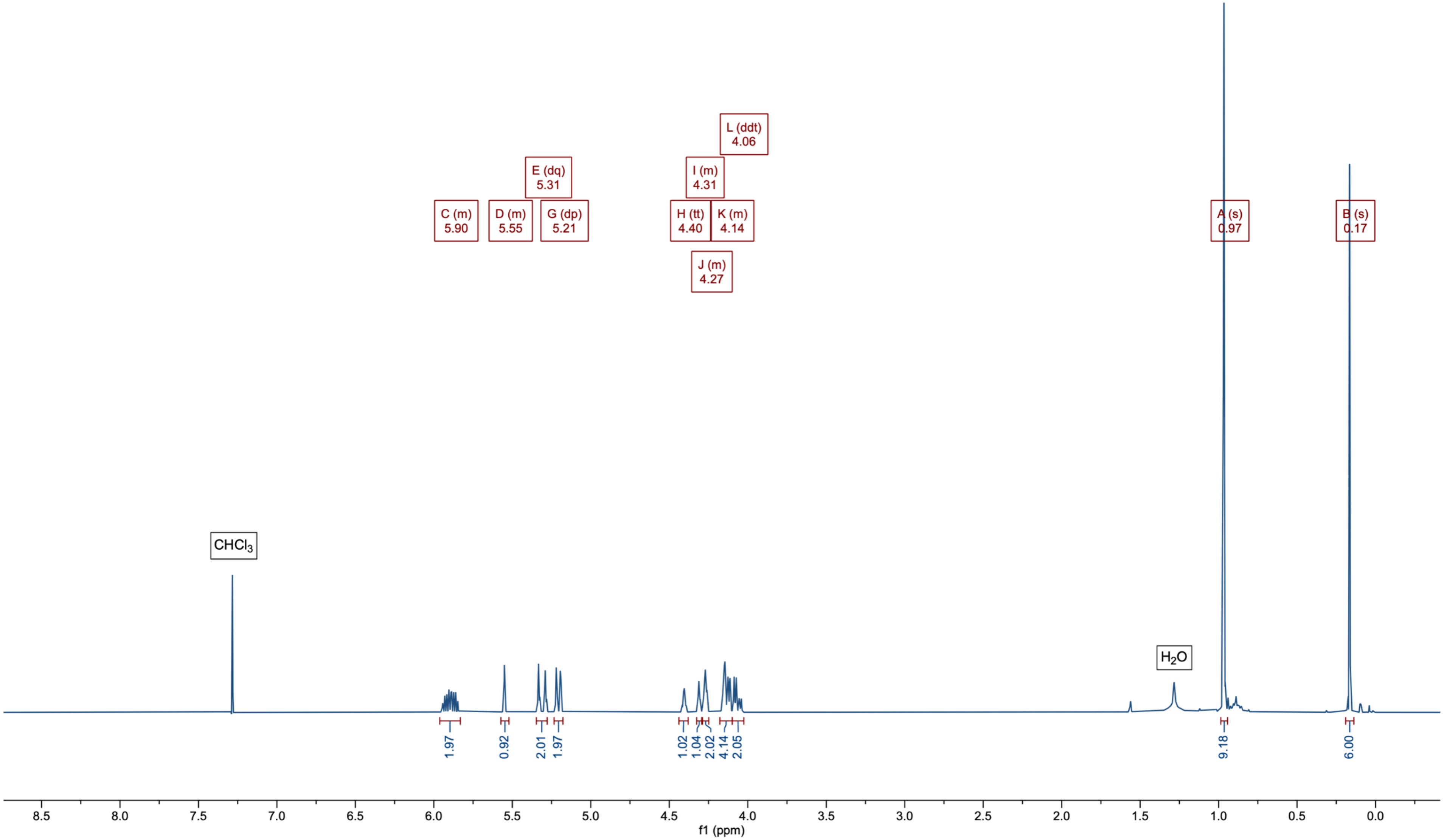

Compound 8: $^{13}C\{^{1}H\}$ – NMR ($CDCl_3$, 101 MHz)

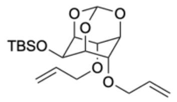

8

$C_{19}H_{32}O_6Si$
MW: 384,5440

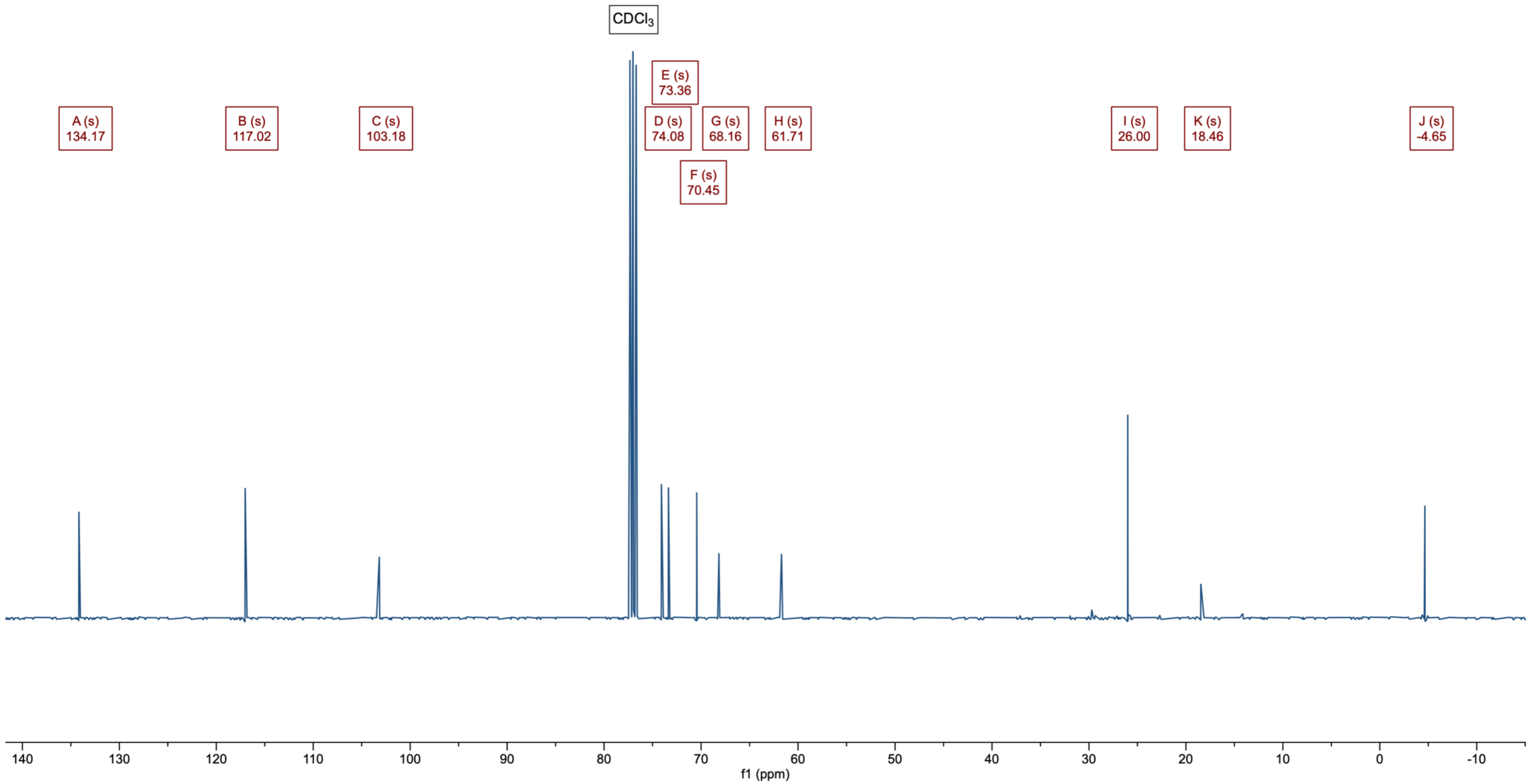

Compound 9: ^{1}H – NMR (MeCN-d_3, 400 MHz)

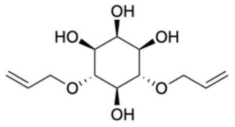

9

$C_{12}H_{20}O_6$
MW: 260,2860

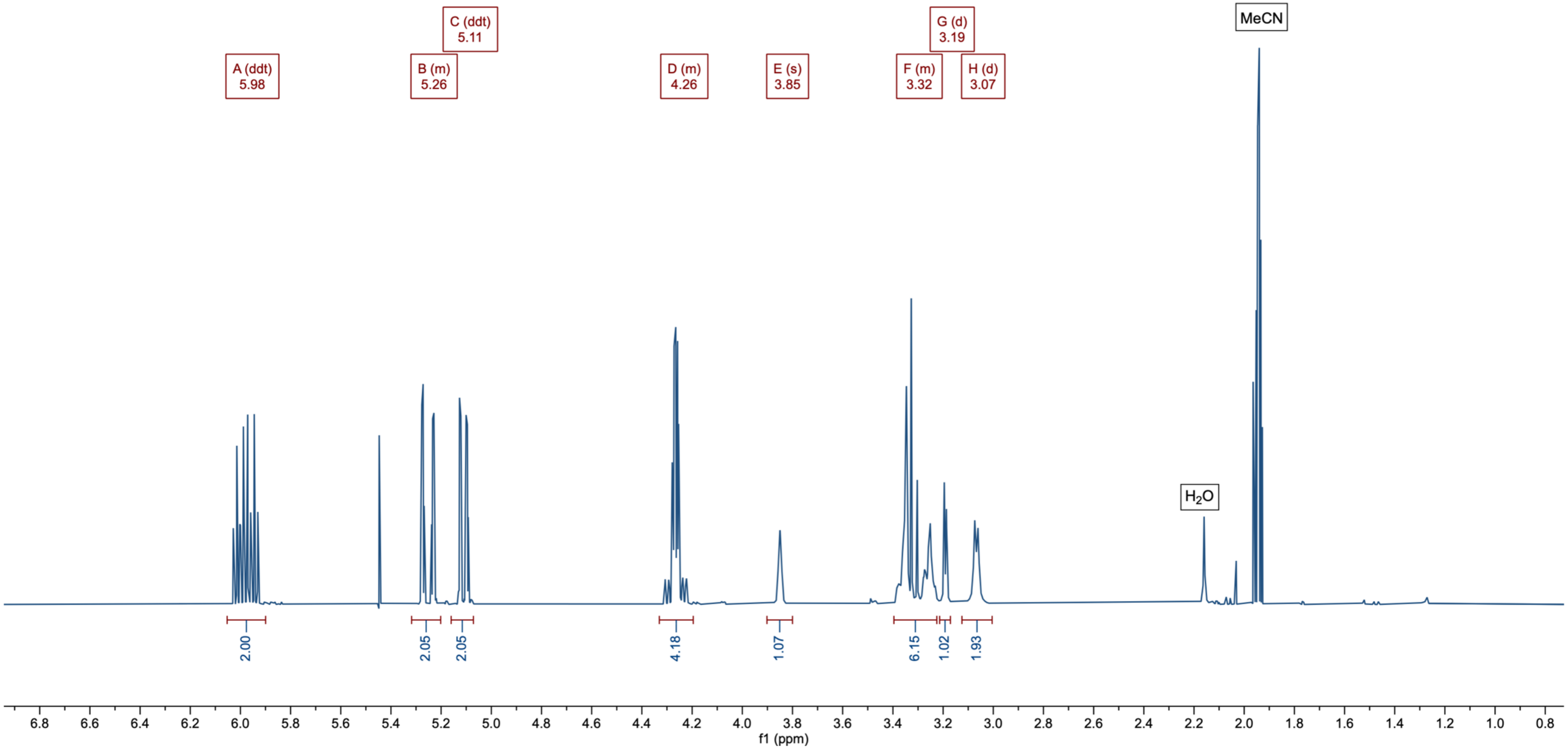

Compound 9: $^{13}C\{^{1}H\}$ – NMR (MeCN-d_3, 101 MHz)

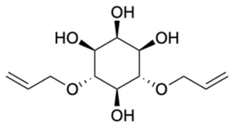

9

$C_{12}H_{20}O_6$
MW: 260,2860

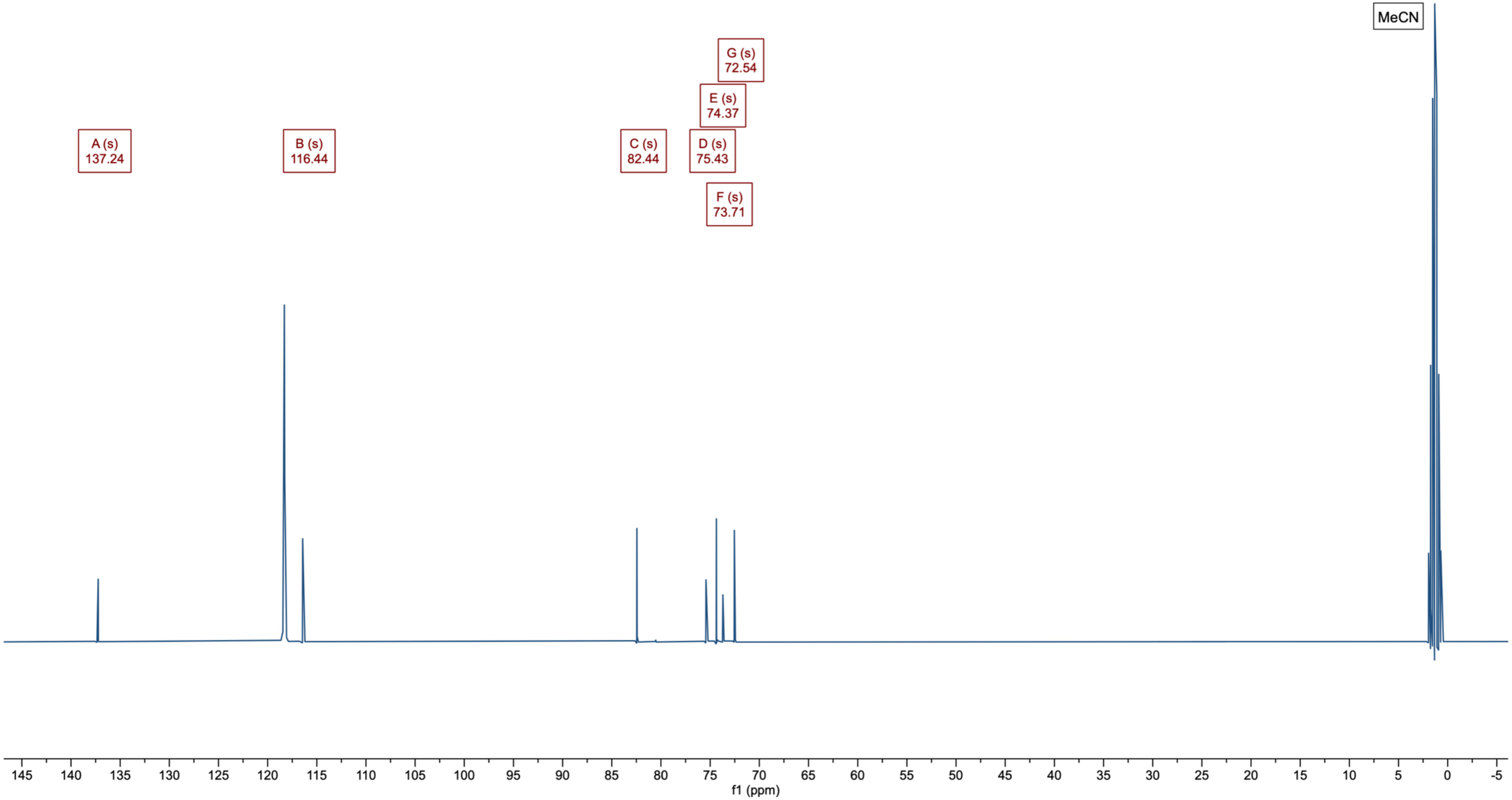

Compound 10: $^{1}H-NMR$ ($CDCl_3$, 400 MHz)

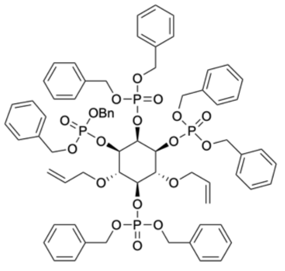

10

$C_{68}H_{72}O_{18}P_4$
MW: 1301,2010

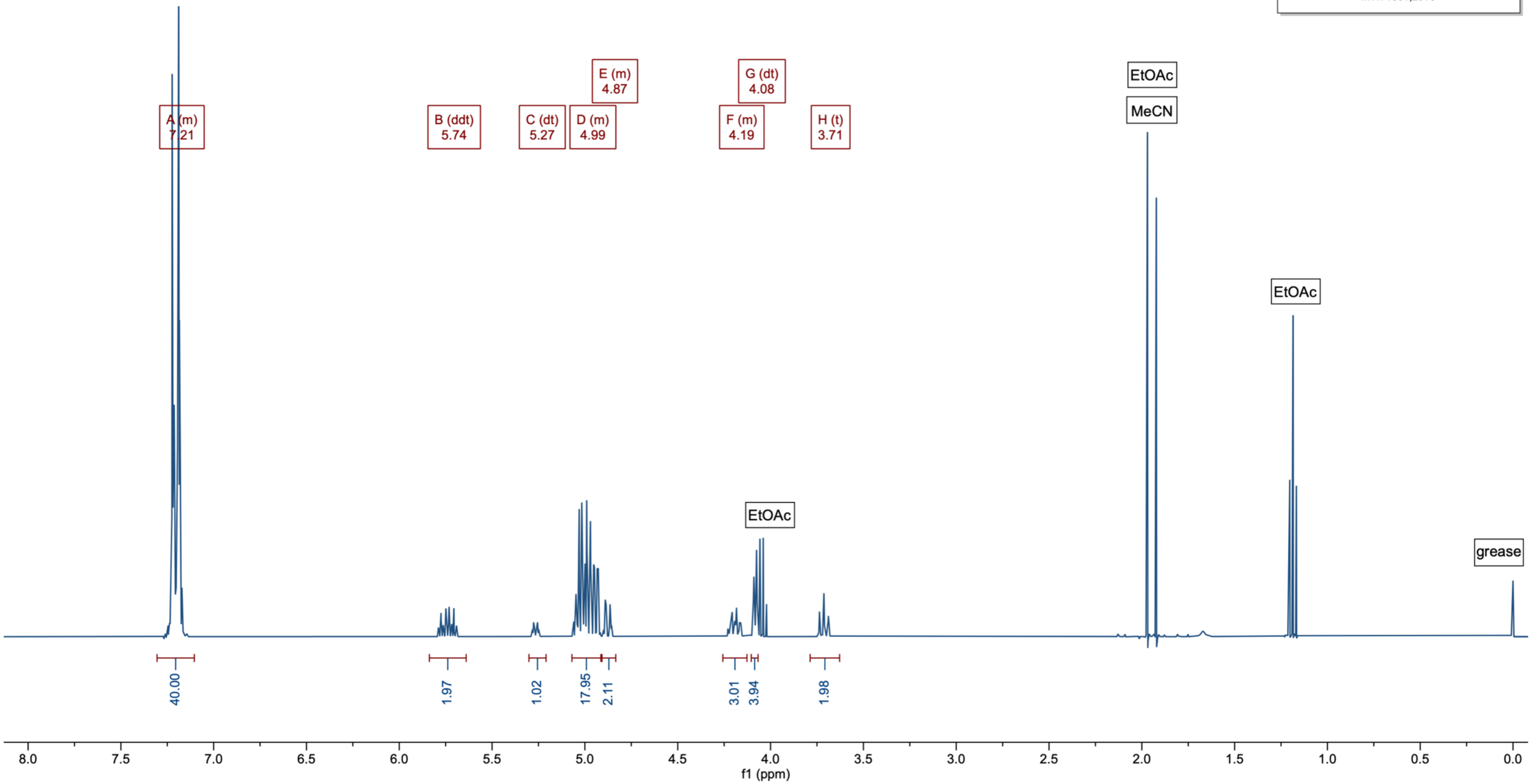

Compound 10: $^{31}P\{^{1}H\}$ – NMR ($CDCl_3$, 162 MHz)

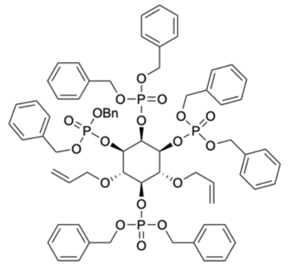

10

$C_{68}H_{72}O_{18}P_4$
MW: 1301,2010

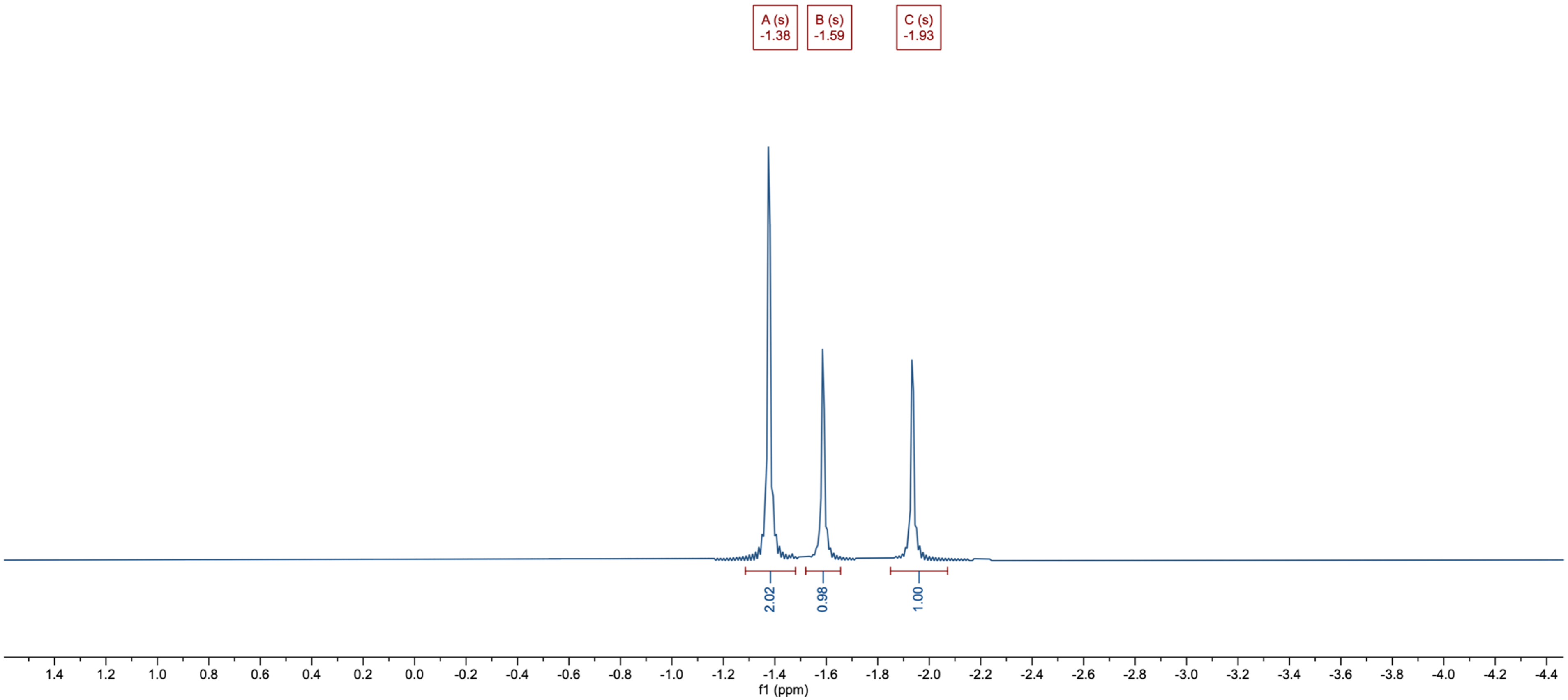

Compound 10: $^{13}C\{^{1}H\}$ – NMR ($CDCl_3$, 101 MHz)

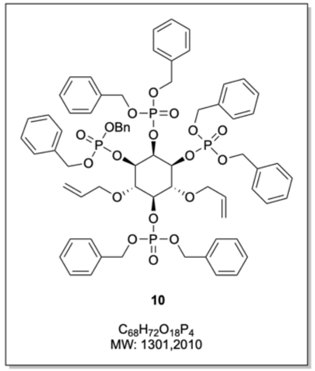

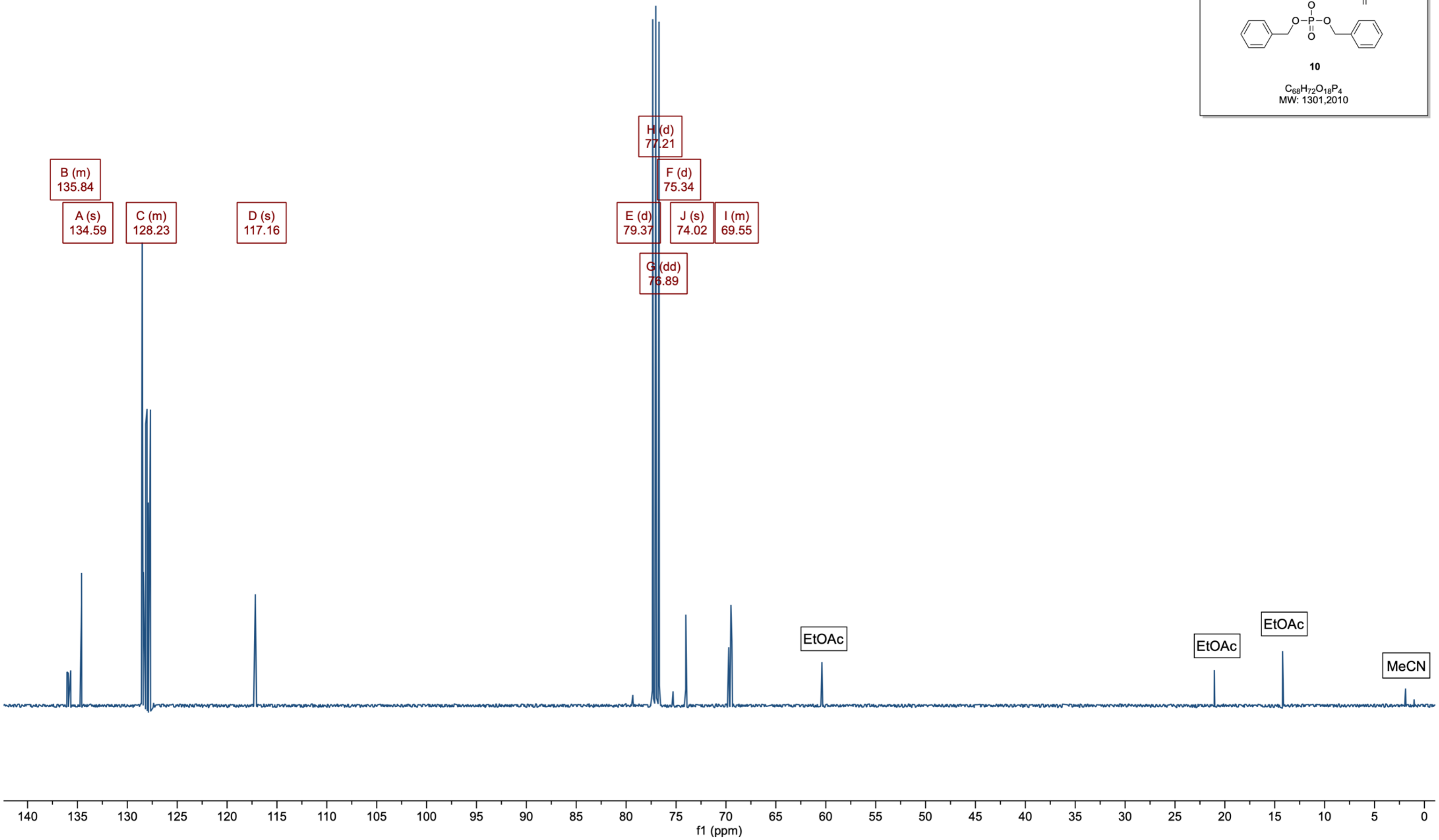

Compound 11: ^{1}H – NMR ($CDCl_3$, 400 MHz)

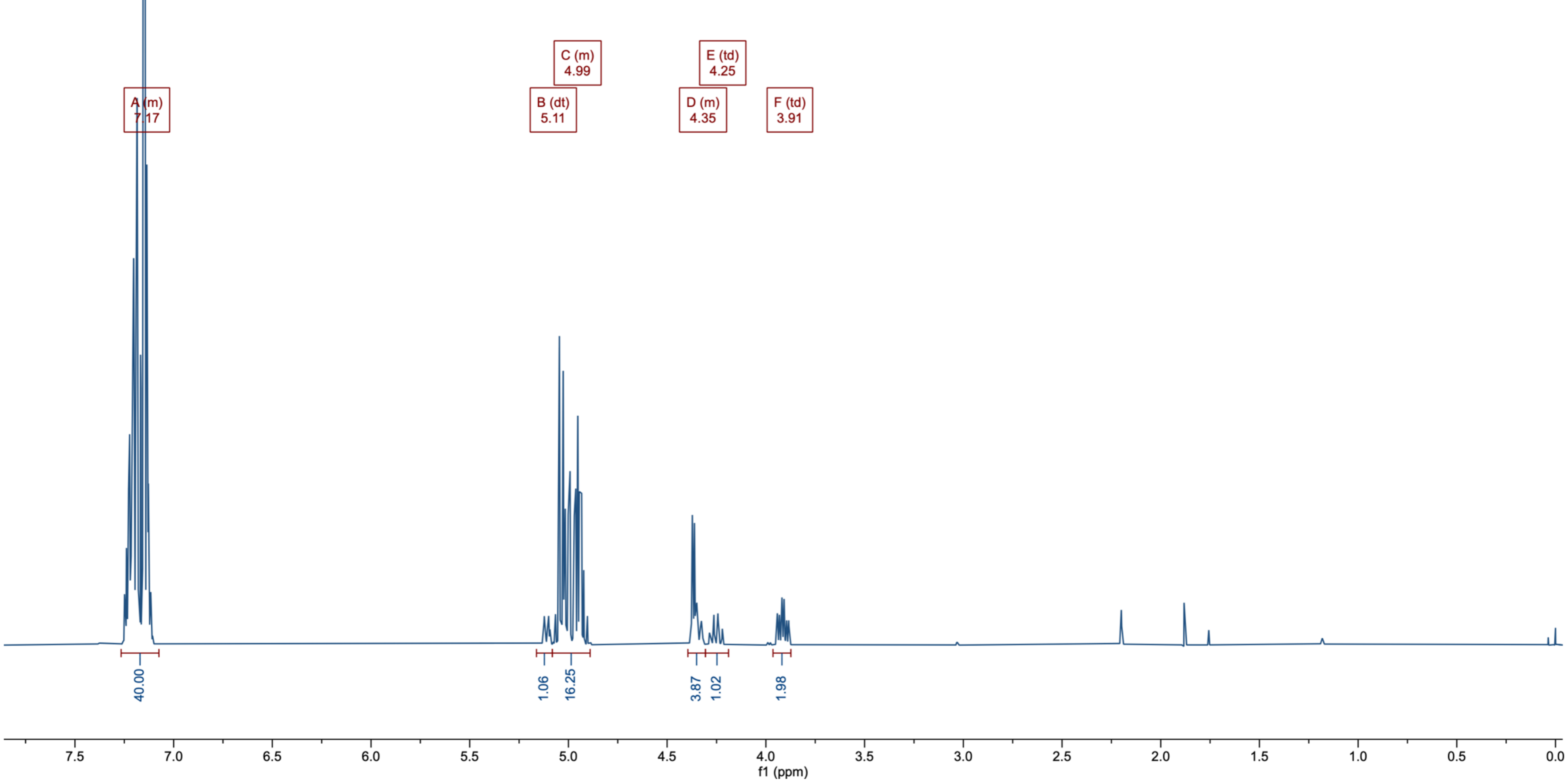

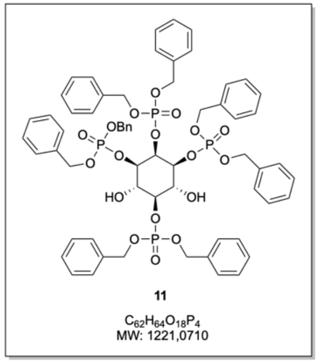

Compound 11: $^{31}P\{^{1}H\}$ – NMR ($CDCl_3$, 162 MHz)

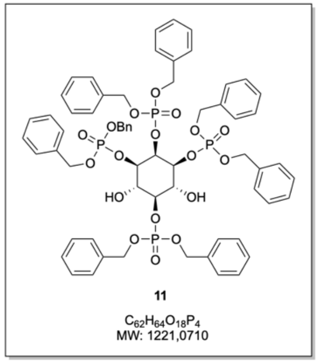

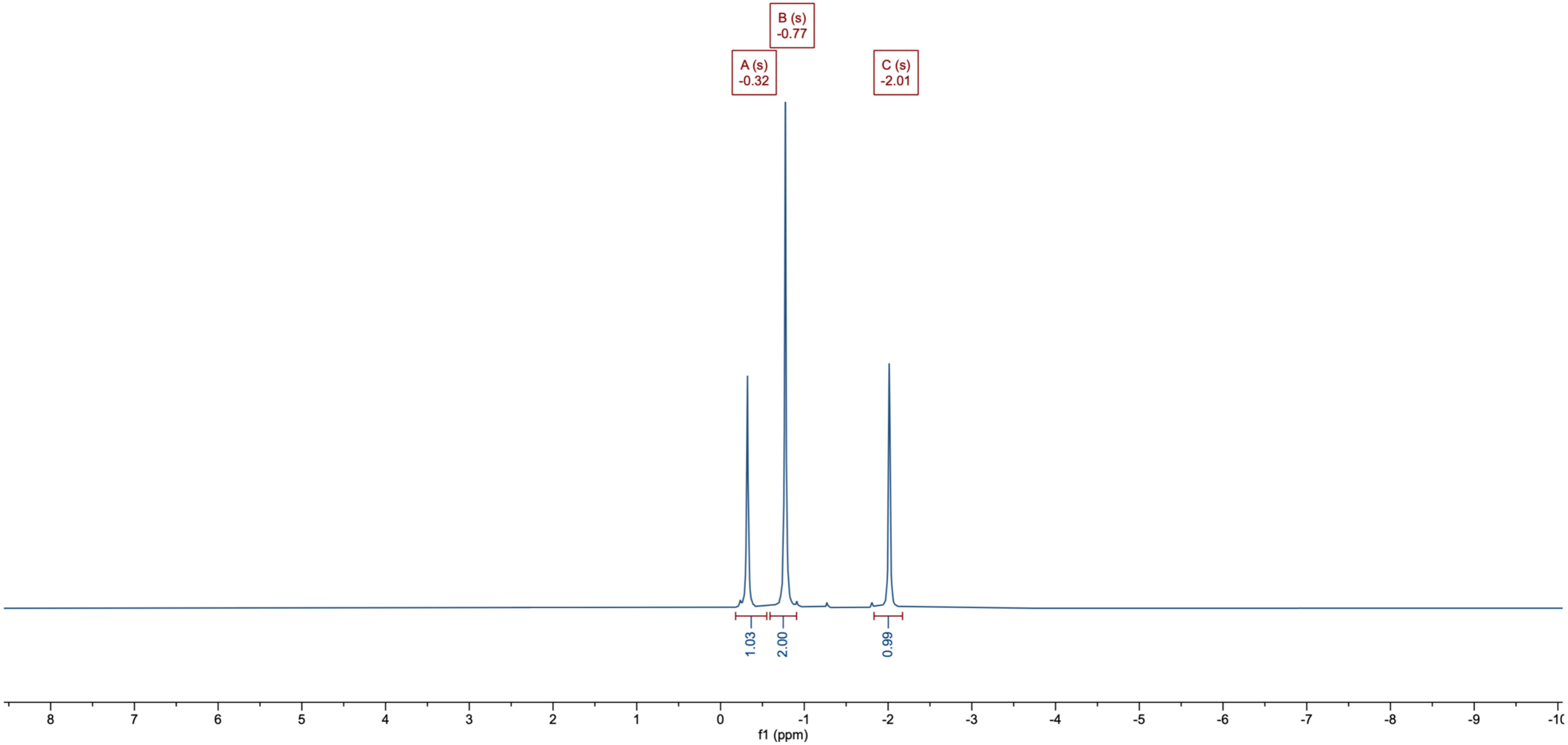

Compound 11: $^{13}C\{^{1}H\}$ – NMR ($CDCl_3$, 101 MHz)

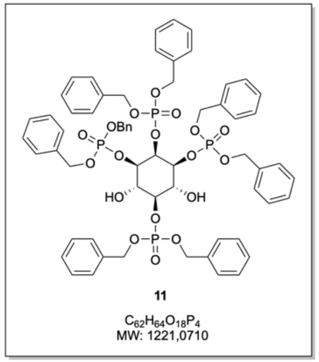

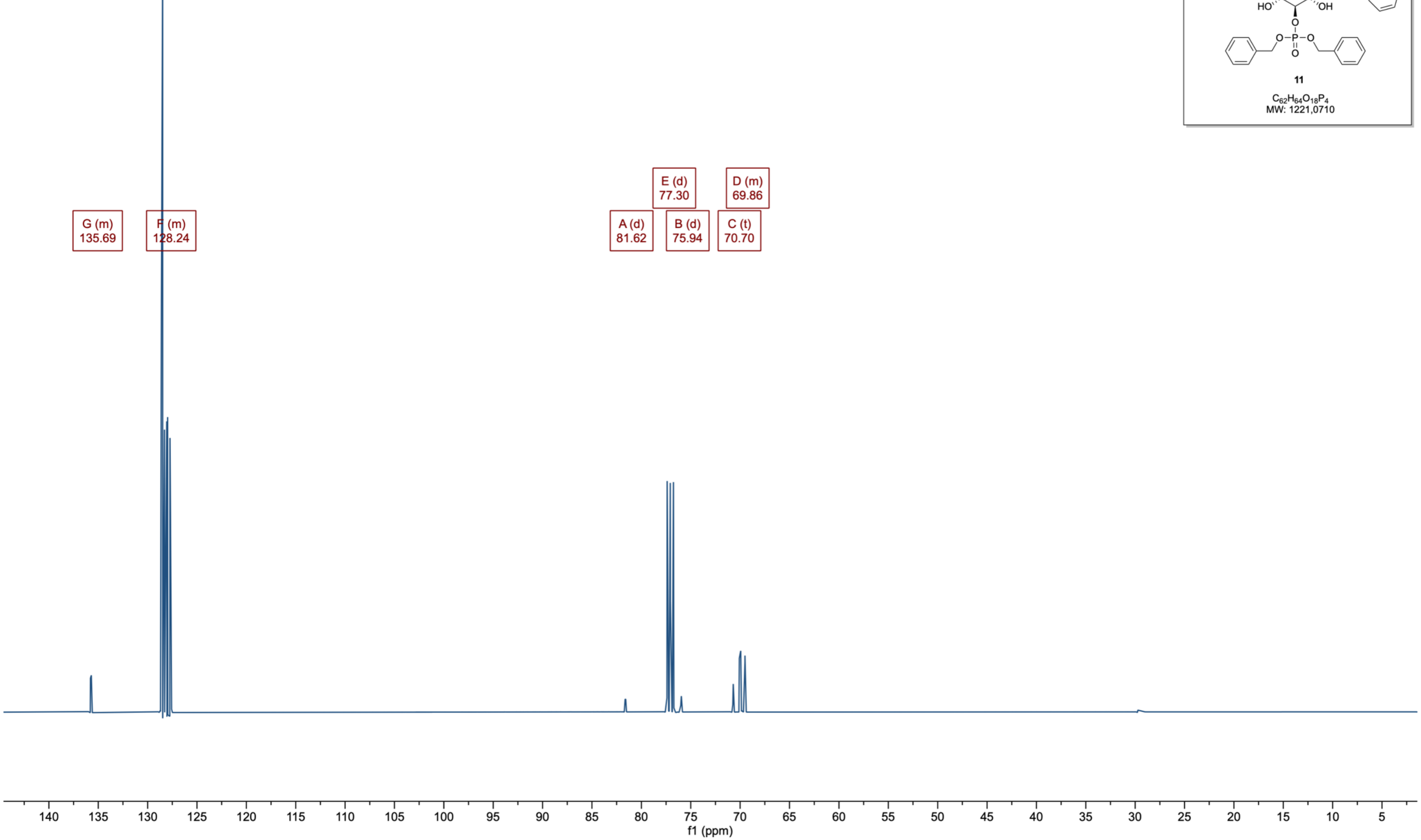

Compound 12: ^{1}H – NMR ($CDCl_3$, 400 MHz)

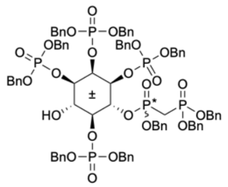

12

$C_{84}H_{86}O_{23}P_6$
MW: 1649,4316

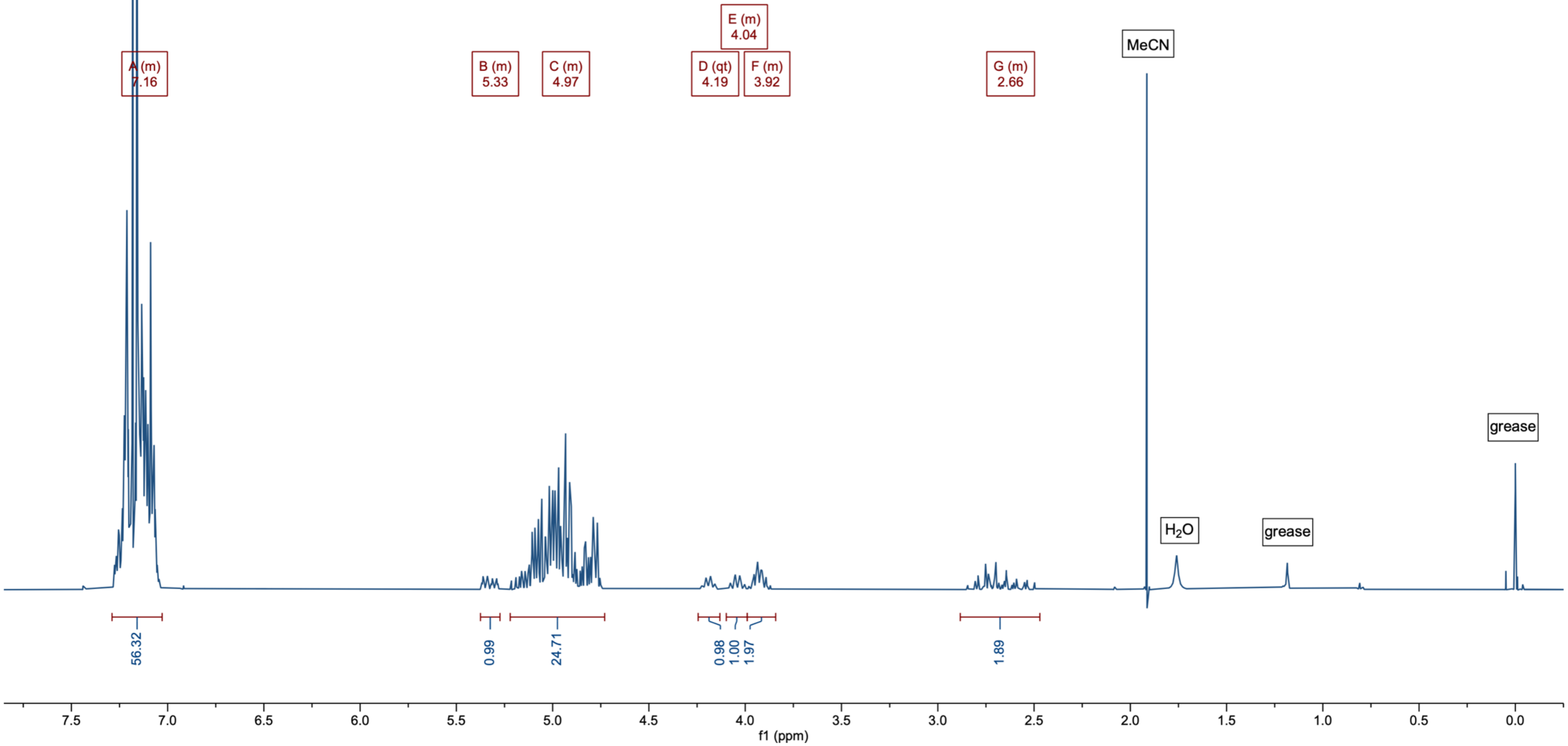

Compound 12: $^{31}P\{^{1}H\}$ – NMR ($CDCl_3$, 162 MHz)

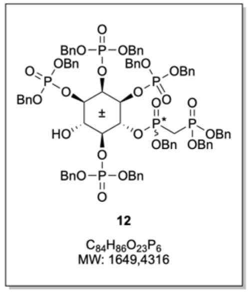

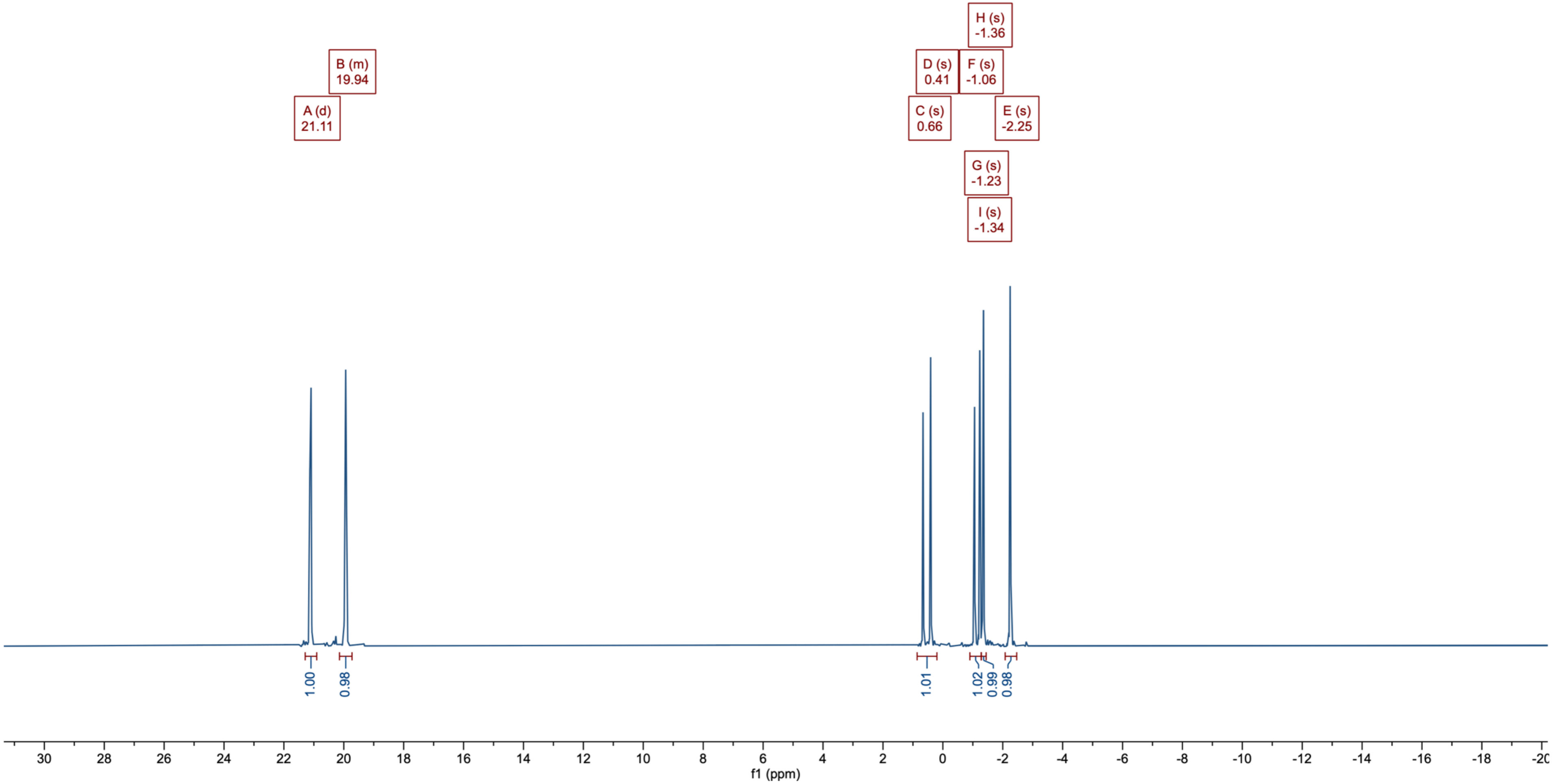

Compound 12: $^{13}C\{^{1}H\}$ – NMR ($CDCl_3$, 101 MHz)

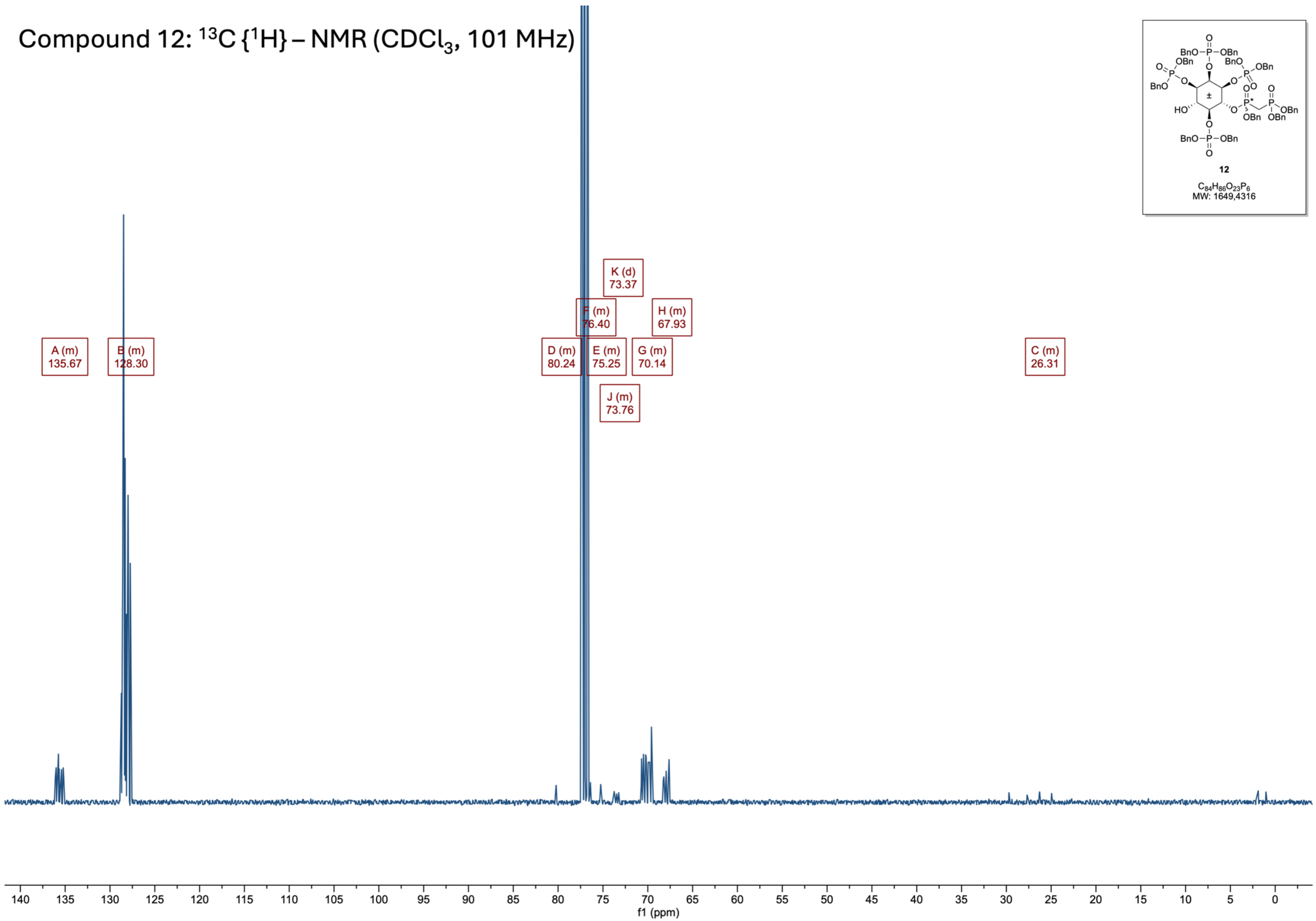

12

$C_{84}H_{86}O_{23}P_6$
MW: 1649,4316

Compound 13: ^{1}H – NMR ($CDCl_3$, 400 MHz)

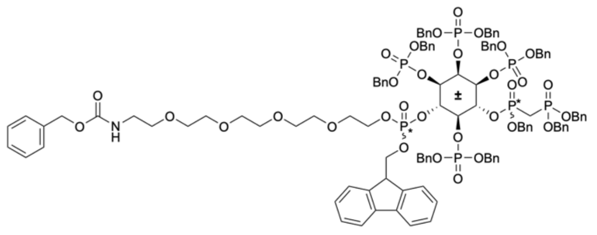

13

$C_{116}H_{124}NO_{32}P_7$
MW: 2261,0593

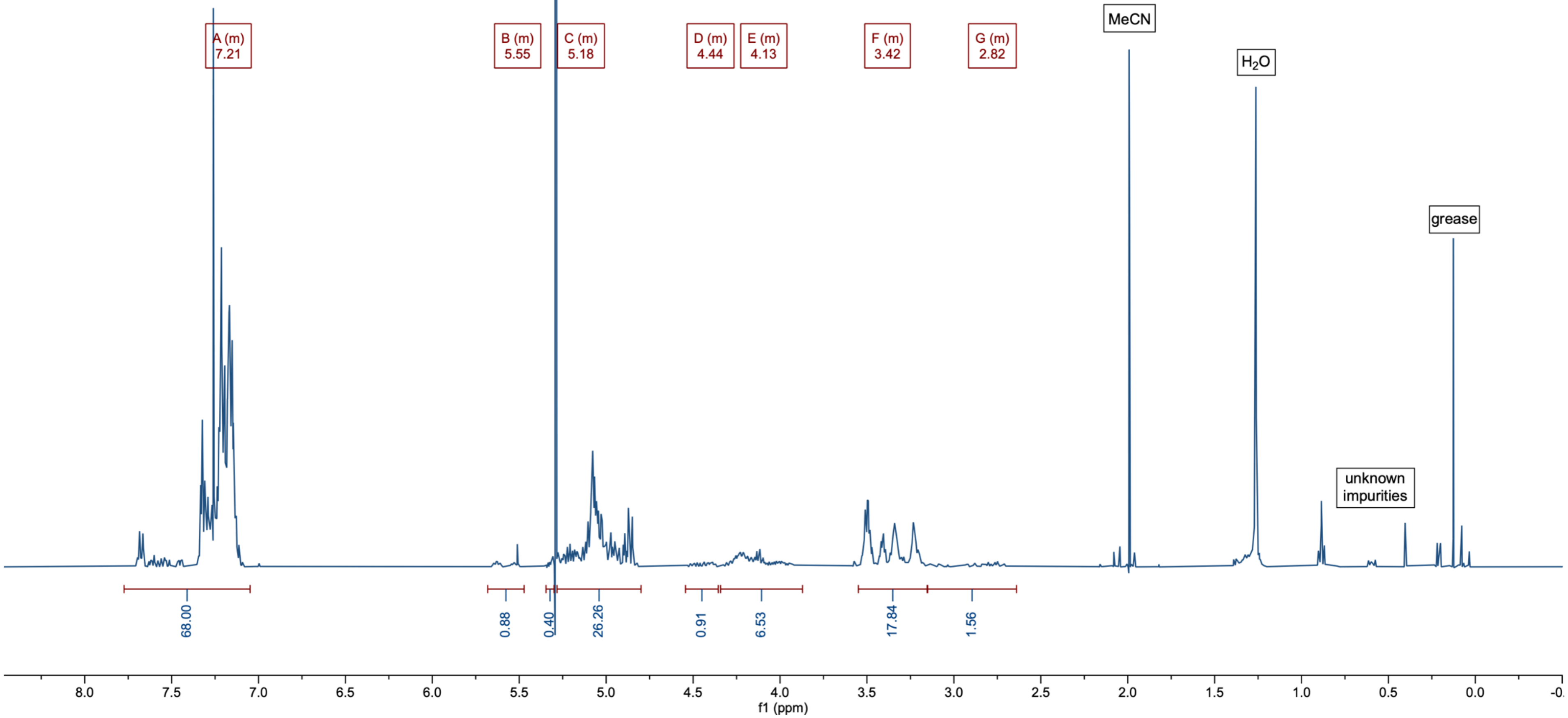

Compound 13: $^{31}P\{^{1}H\}$ – NMR ($CDCl_3$, 162 MHz)

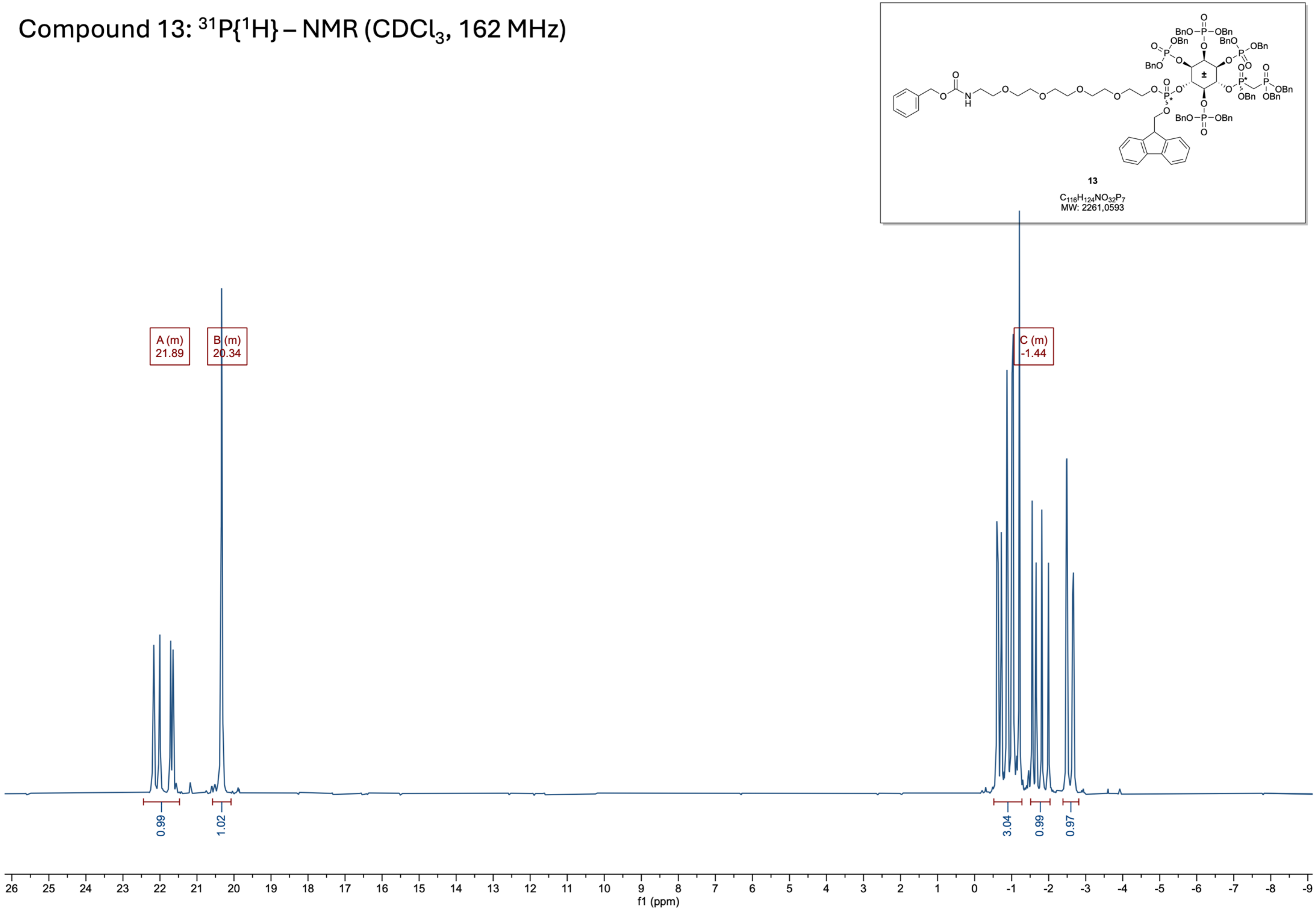

Compound 13: $^{13}C\{^1H\}$ – NMR ($CDCl_3$, 101 MHz)

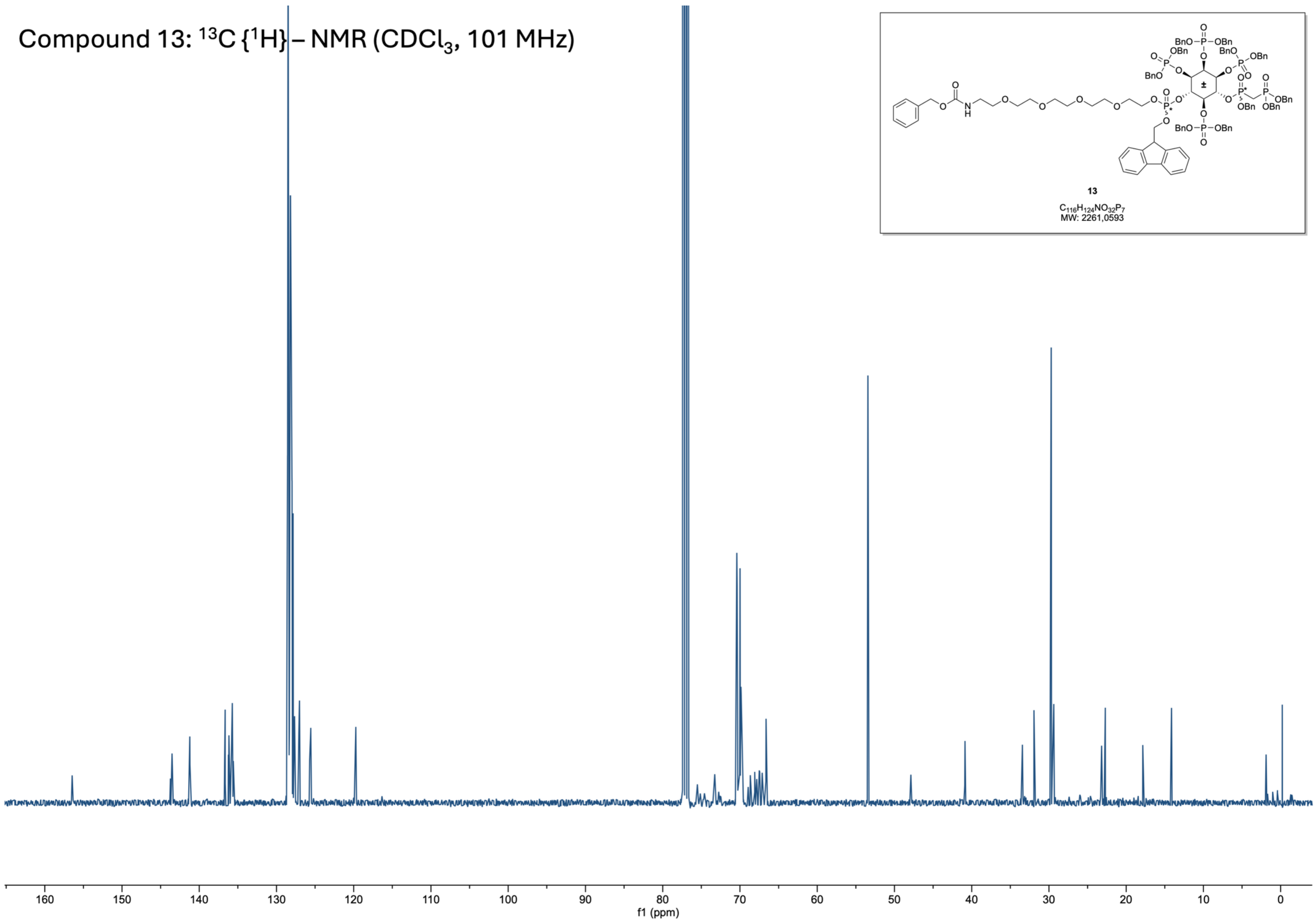

13

$C_{116}H_{124}NO_{32}P_7$
MW: 2261,0593

Compound 14: ^1H – NMR (D_2O, 400 MHz)

Supplemented with $N(iPr)_2$ to enhance resolution

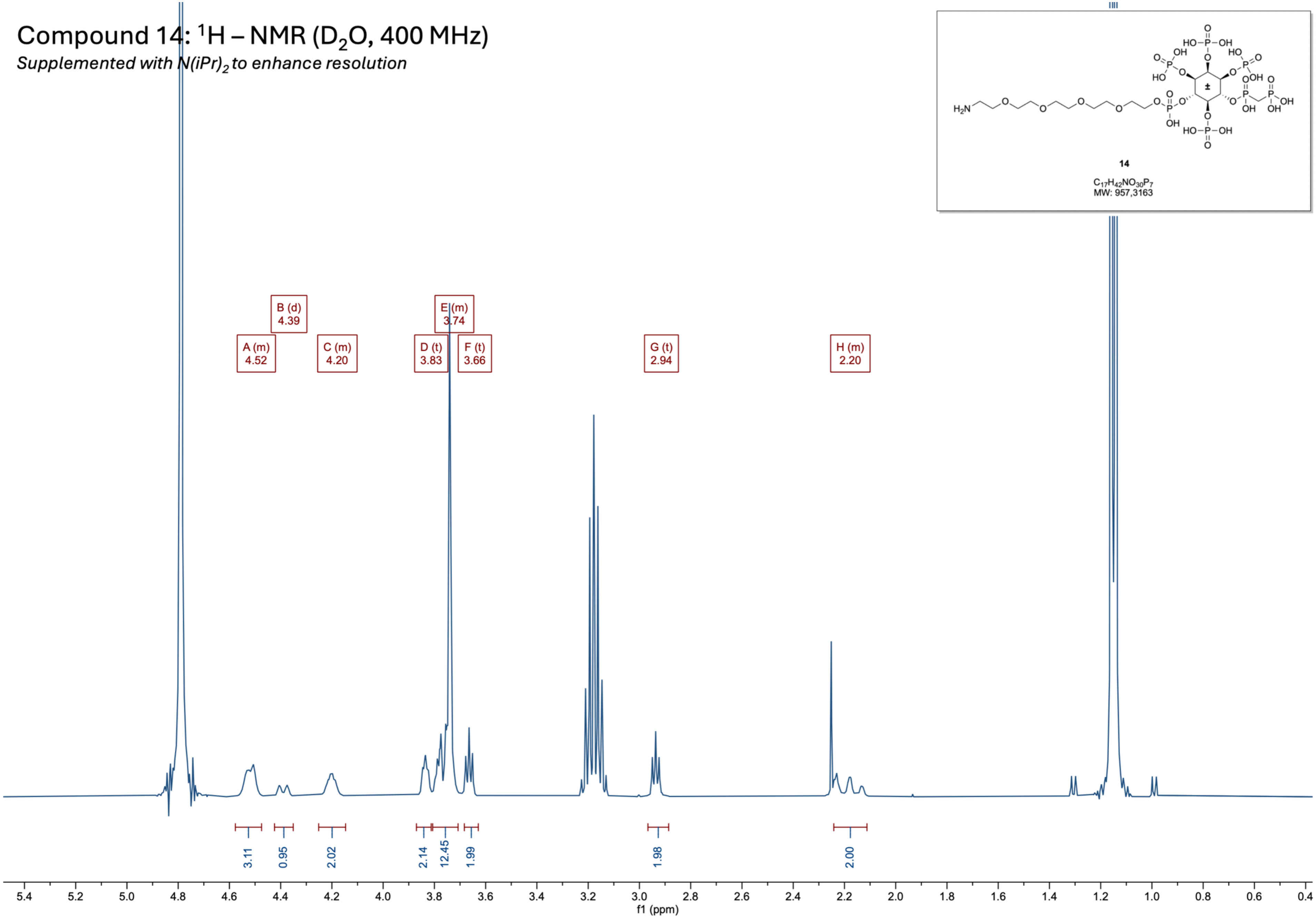

Compound 14: $^{31}P\{^{1}H\}$ – NMR (D_2O, 162 MHz)

Supplemented with $N(iPr)_2$ to enhance resolution

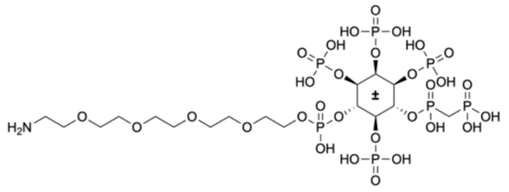

14

$C_{17}H_{42}NO_{30}P_7$
MW: 957,3163

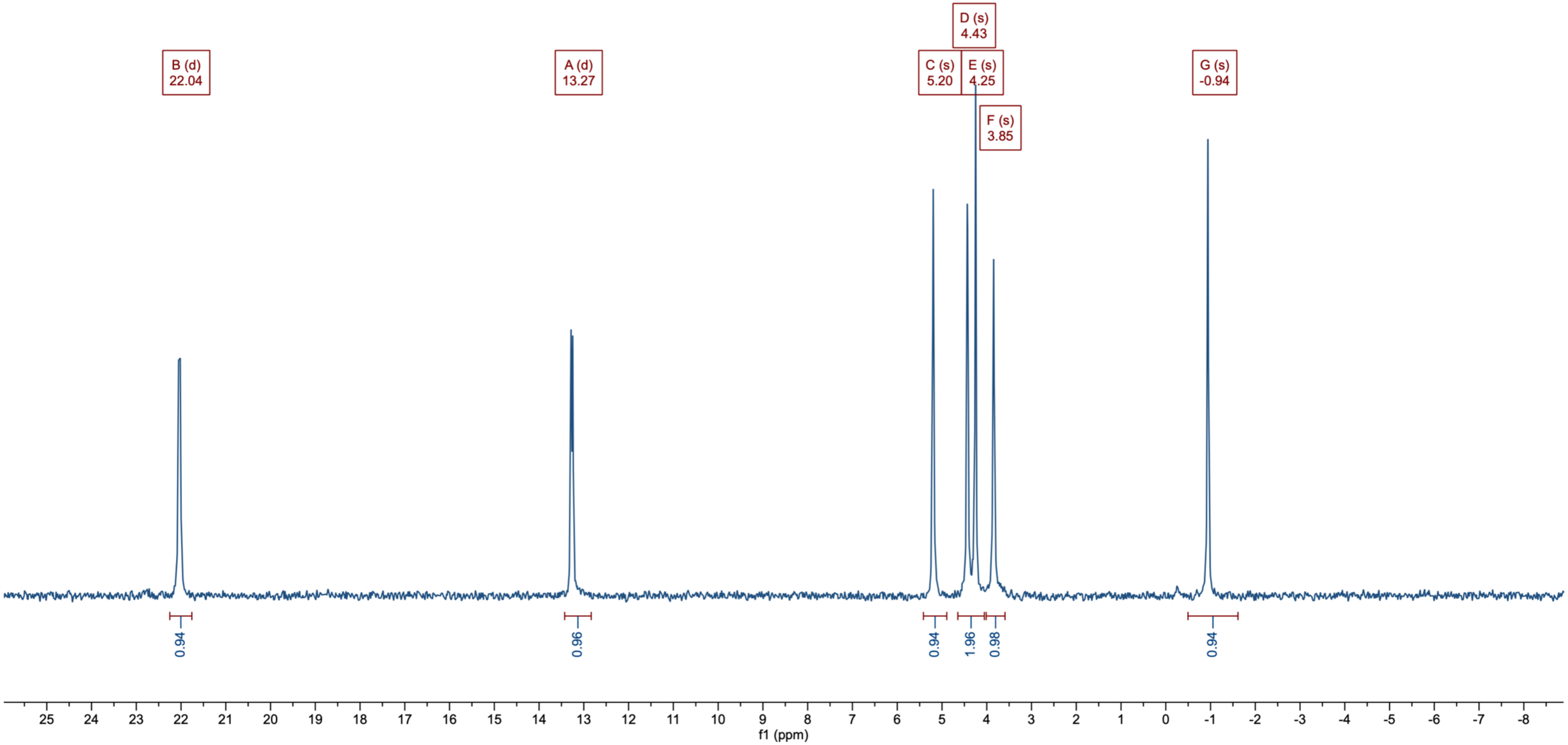

Compound 14: $^{13}C\{^1H\}$ – NMR (D_2O, 101 MHz)

Supplemented with $N(iPr)_2$ to enhance resolution

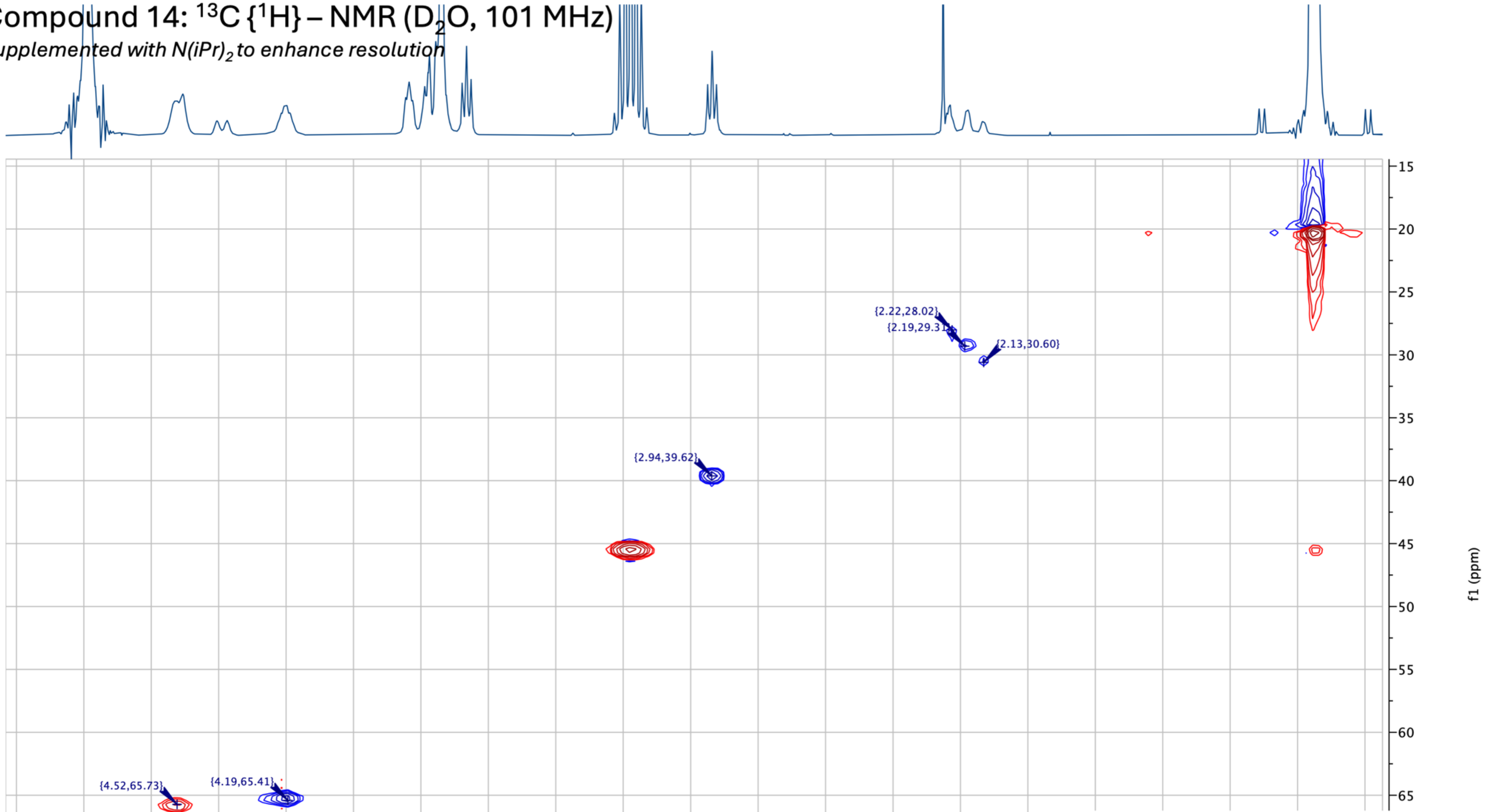

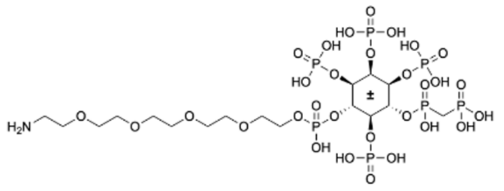

14

$C_{17}H_{42}NO_{30}P_7$
MW: 957,3163

Compound 16: ^{1}H – NMR ($CDCl_3$, 400 MHz)

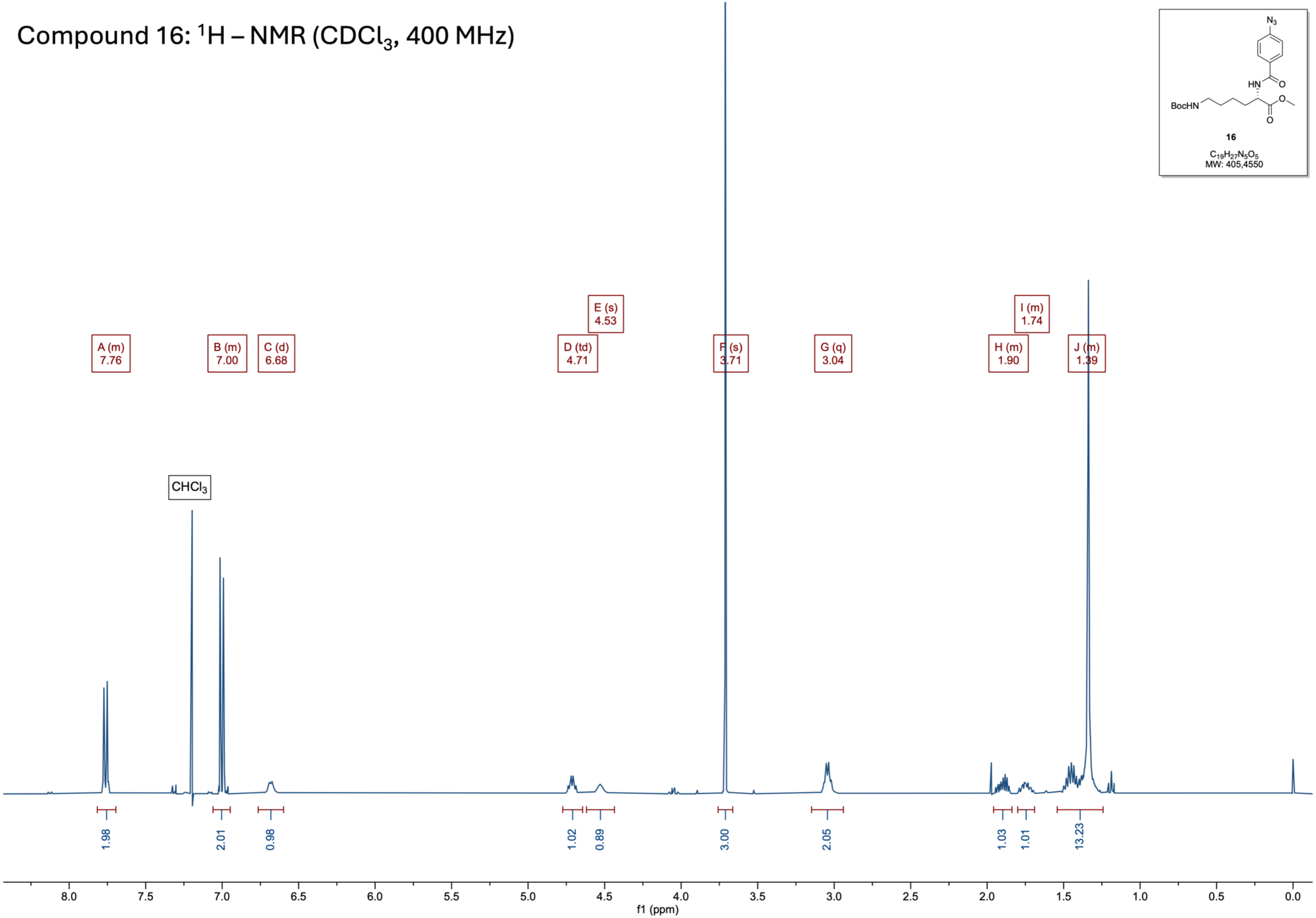

Compound 16: $^{13}C\{^1H\}$ – NMR ($CDCl_3$, 101 MHz)

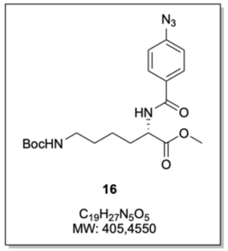

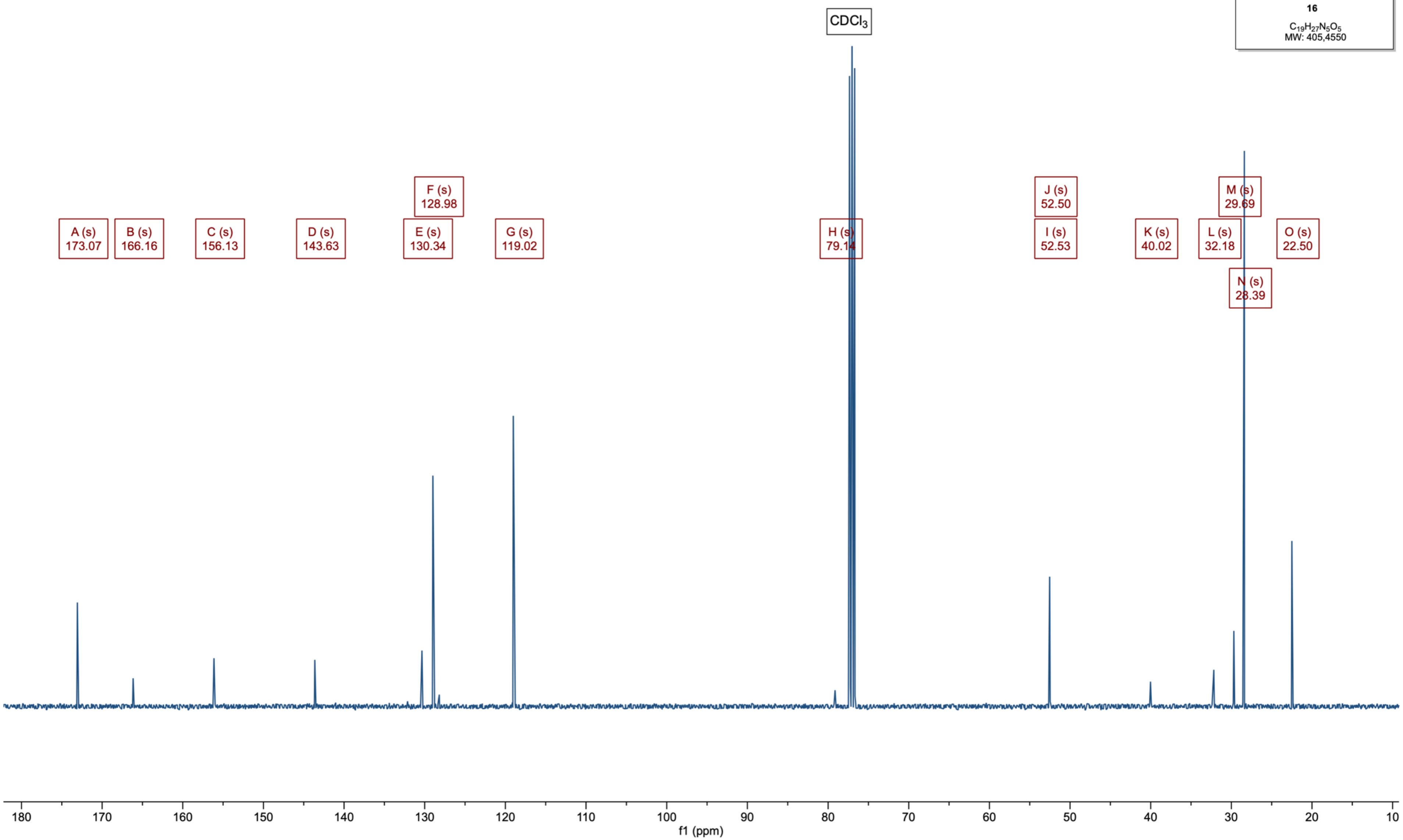

Compound 17: ^1H – NMR ($CDCl_3$, 400 MHz)

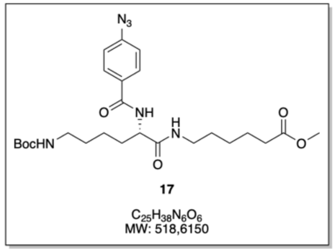

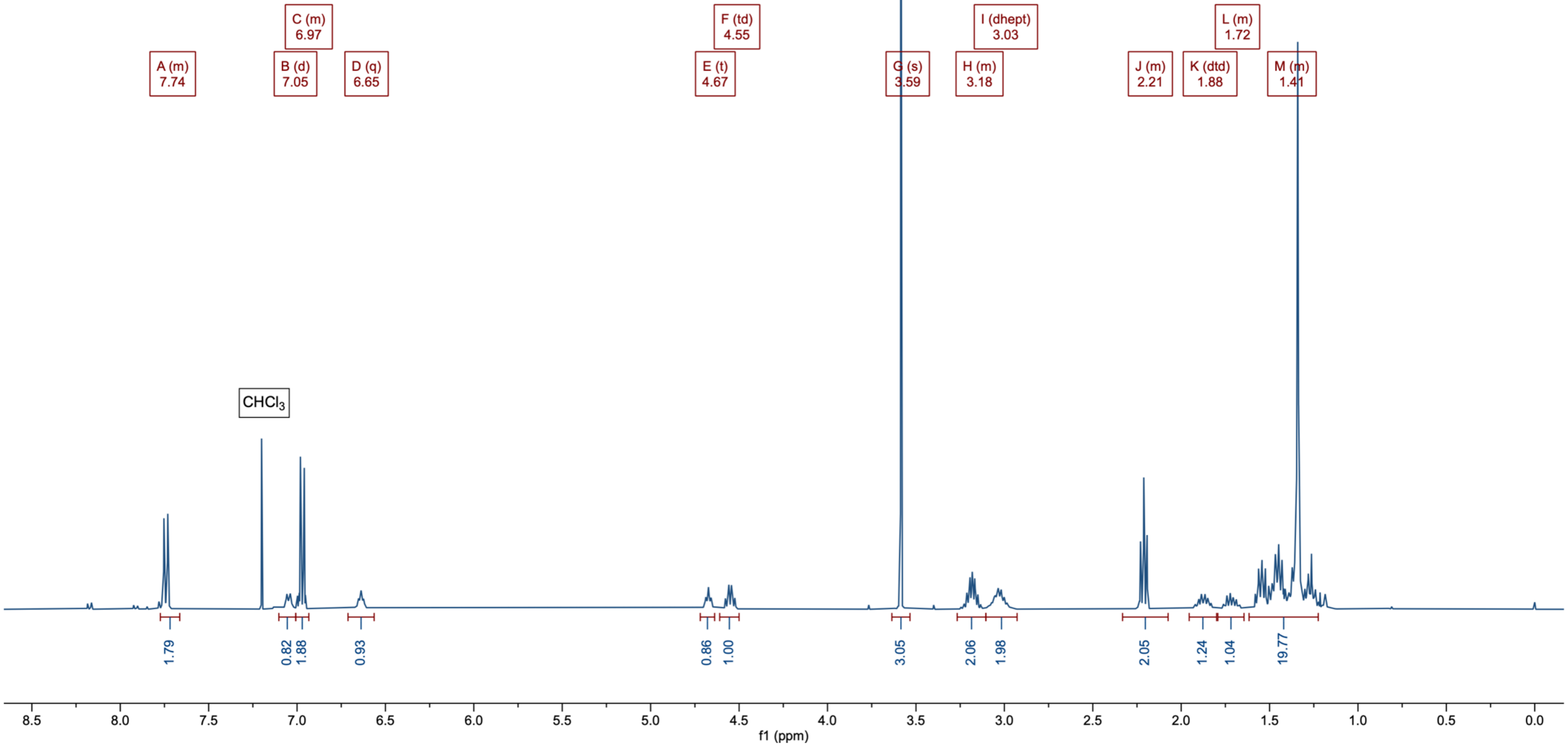

Compound 17: $^{13}C\{^{1}H\}$ – NMR ($CDCl_3$, 101 MHz)

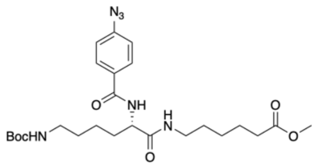

17

$C_{25}H_{38}N_6O_6$
MW: 518,6150

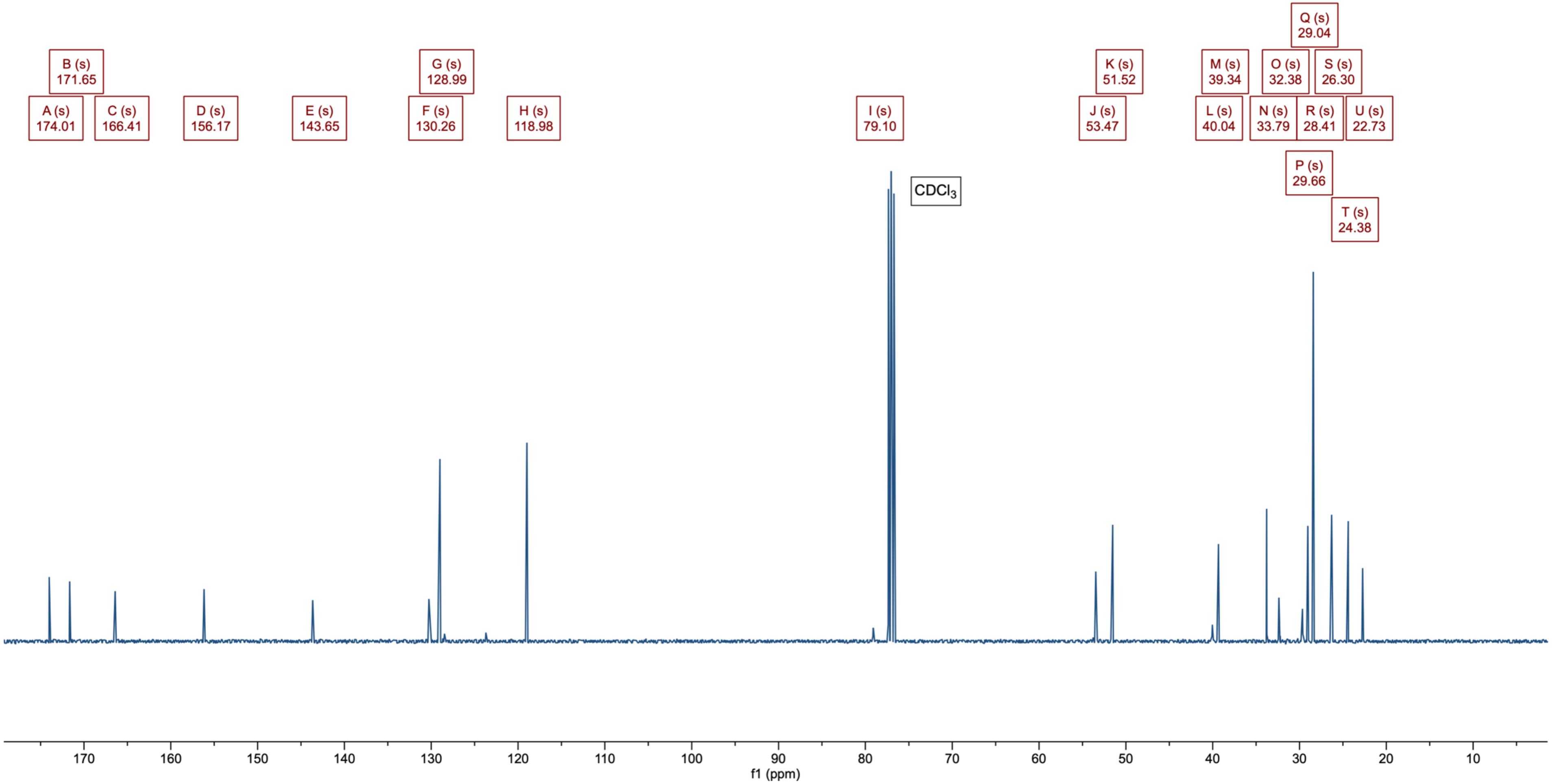

Compound 18: ^{1}H – NMR ($CDCl_3$, 400 MHz)

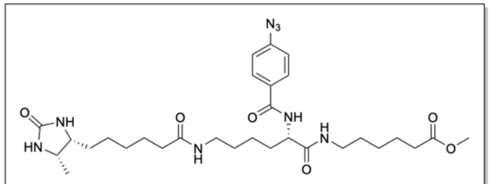

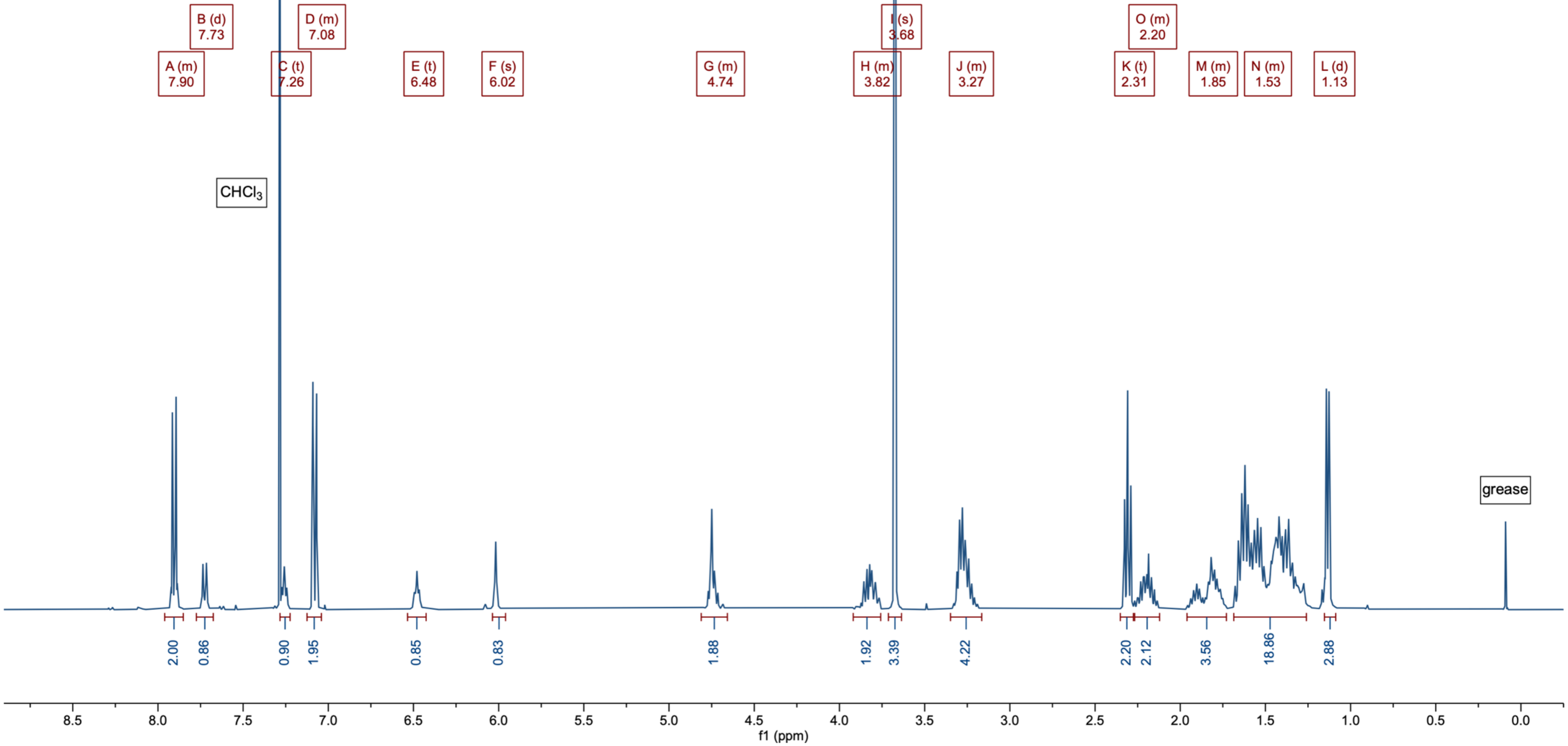

Compound 18: $^{13}C\{^{1}H\}$ – NMR ($CDCl_3$, 101 MHz)

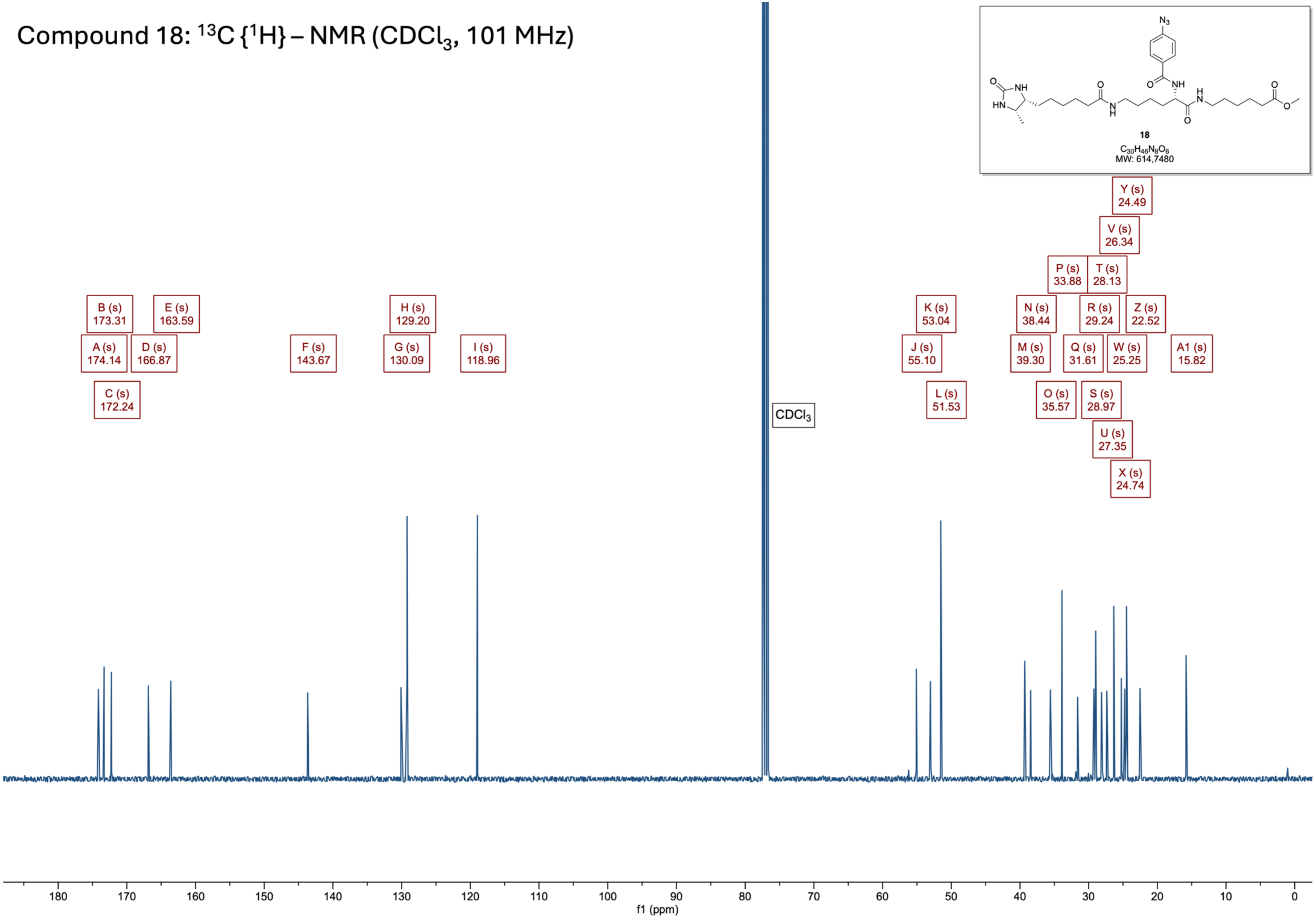

Compound 19: ^{1}H – NMR (DMSO-d_6, 400 MHz)

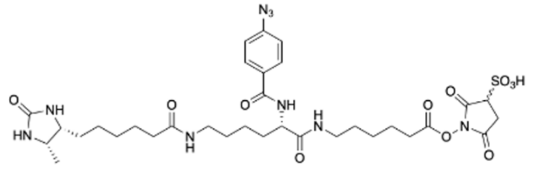

19

$C_{33}H_{47}N_9O_{11}S$

MW: 777,8510

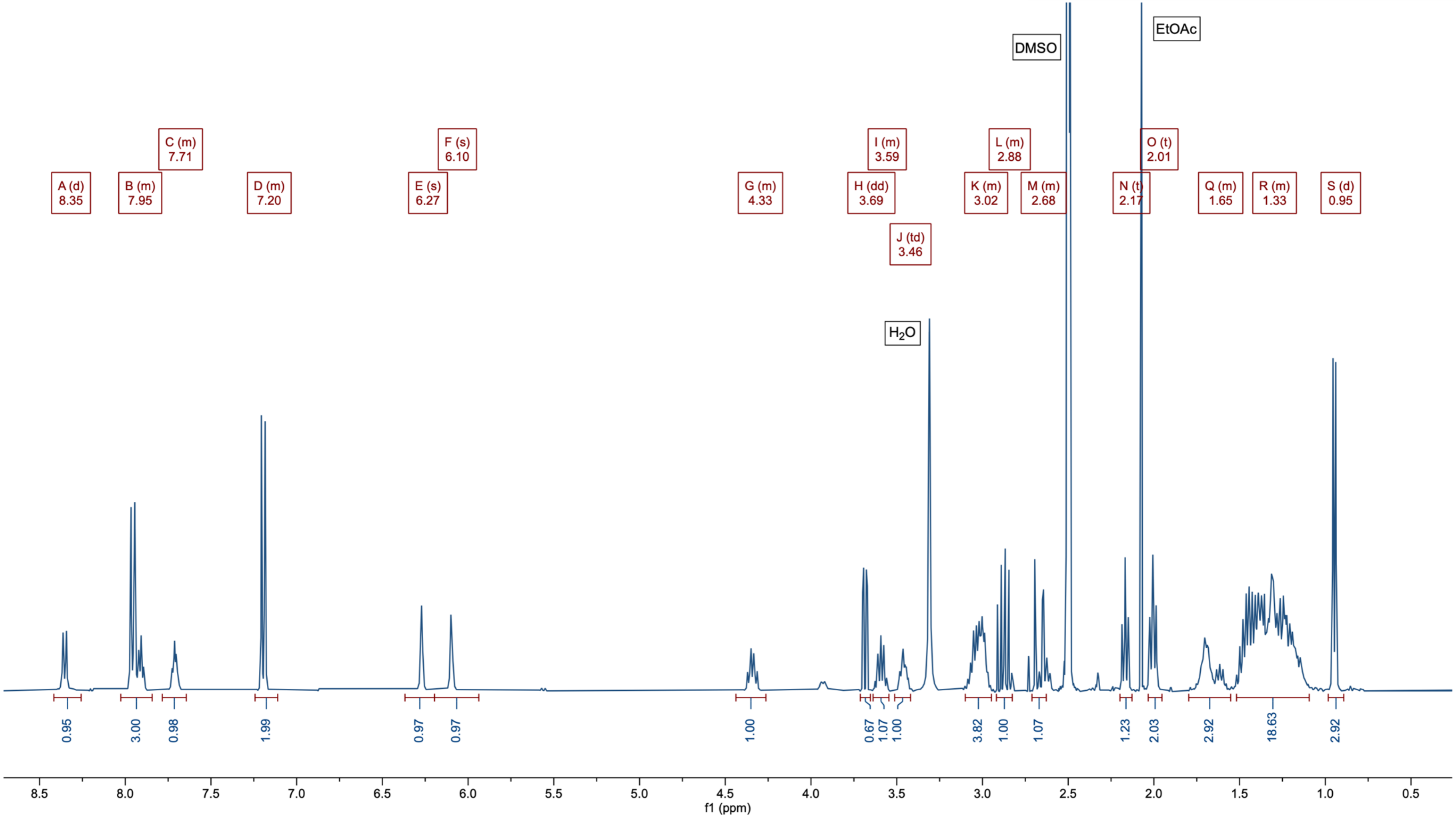

Compound 19: $^{13}C\{^1H\}$ – NMR (DMSO-d_6, 101 MHz)

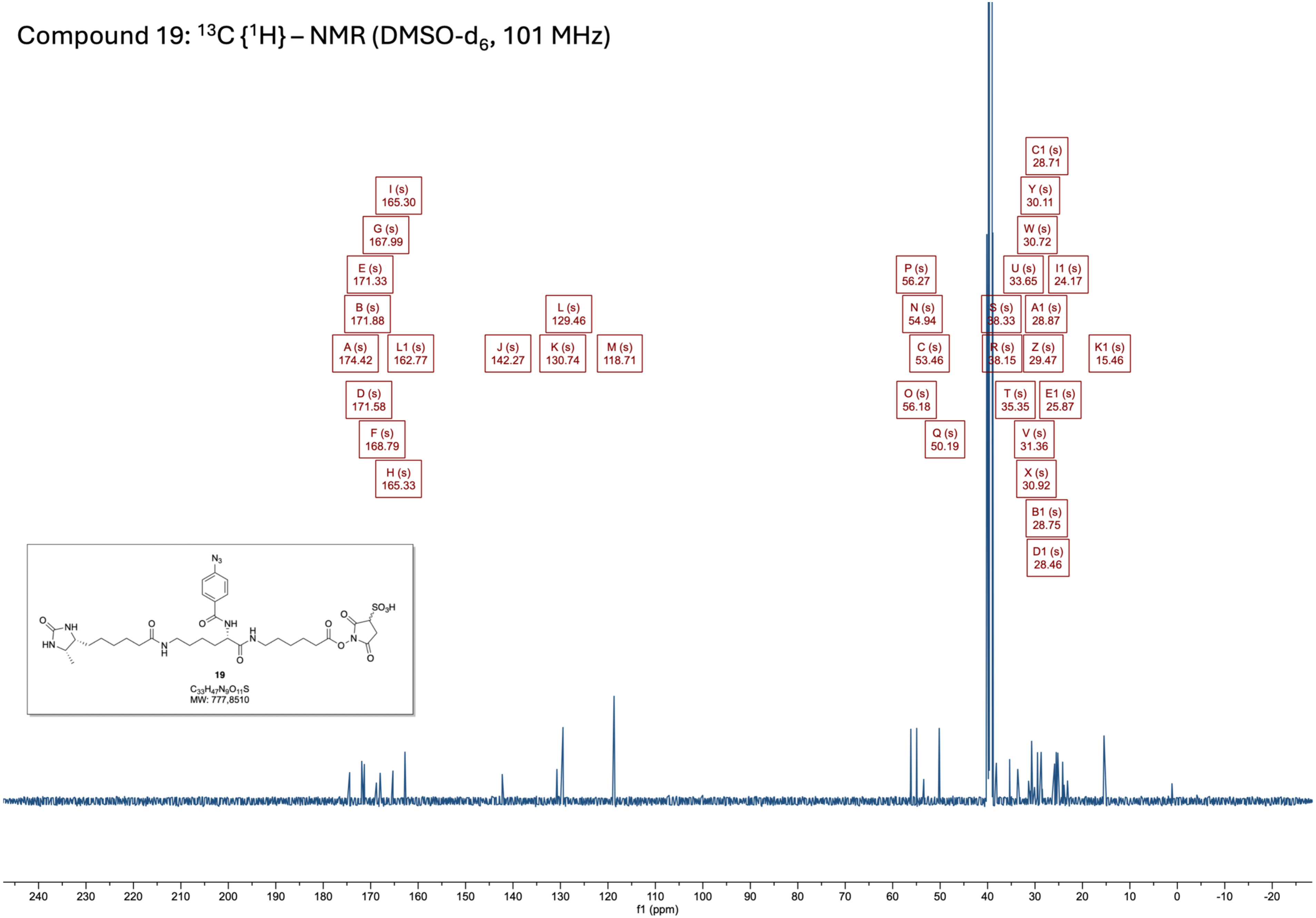

19

$C_{33}H_{47}N_9O_{11}S$

MW: 777,8510

Compound 20: ^1H – NMR (MeOH-d_4, 400 MHz)

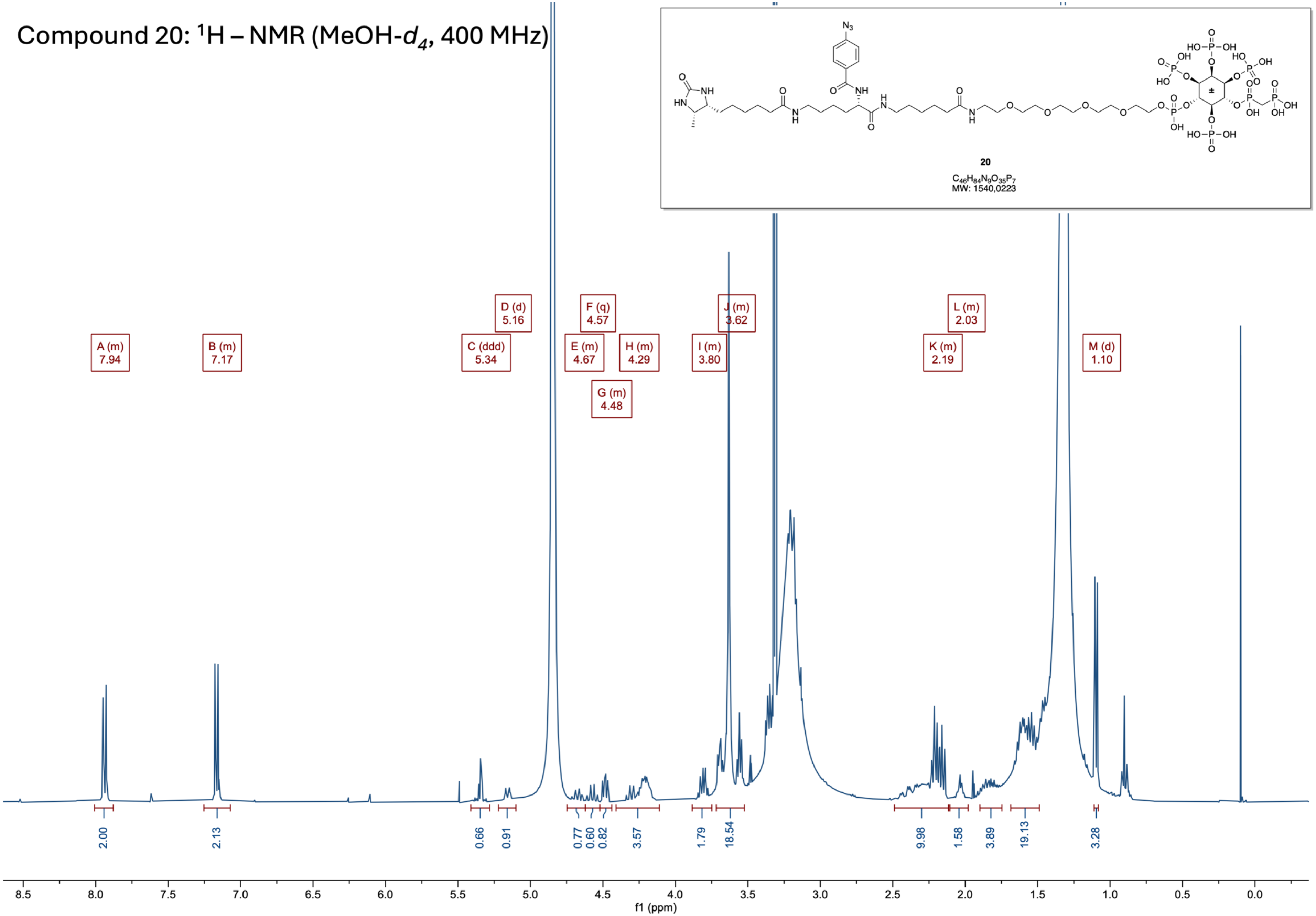

Compound 20: $^{31}P\{^{1}H\}$ – NMR (MeOH-d_4, 162 MHz)

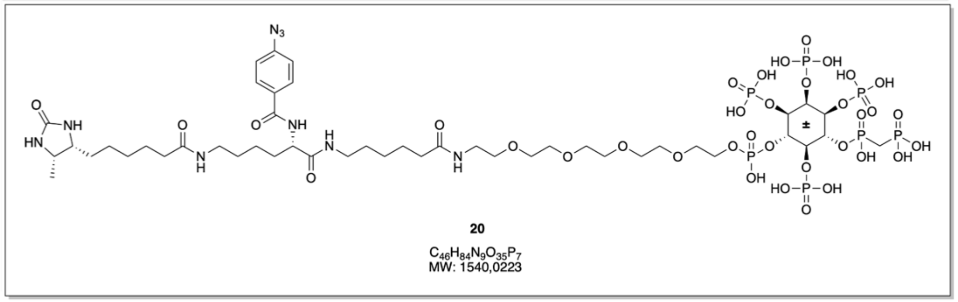

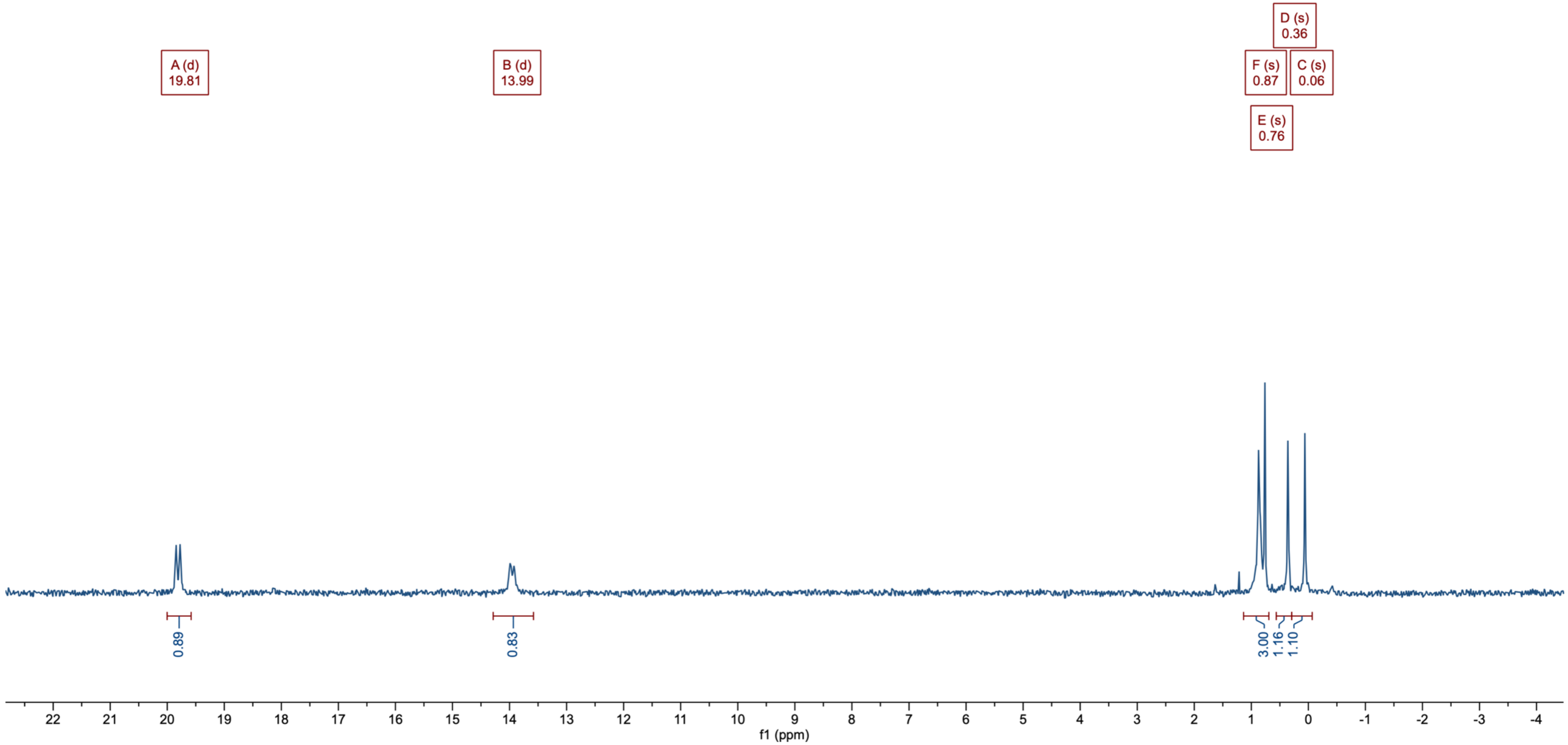

Compound 20: $^{13}C\{^{1}H\}$ – NMR (MeOH-d_4, 101 MHz)

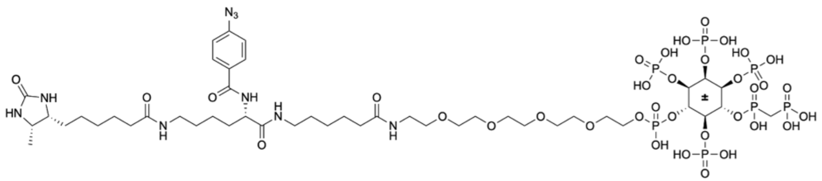

20

$C_{46}H_{84}N_9O_{35}P_7$
MW: 1540,0223

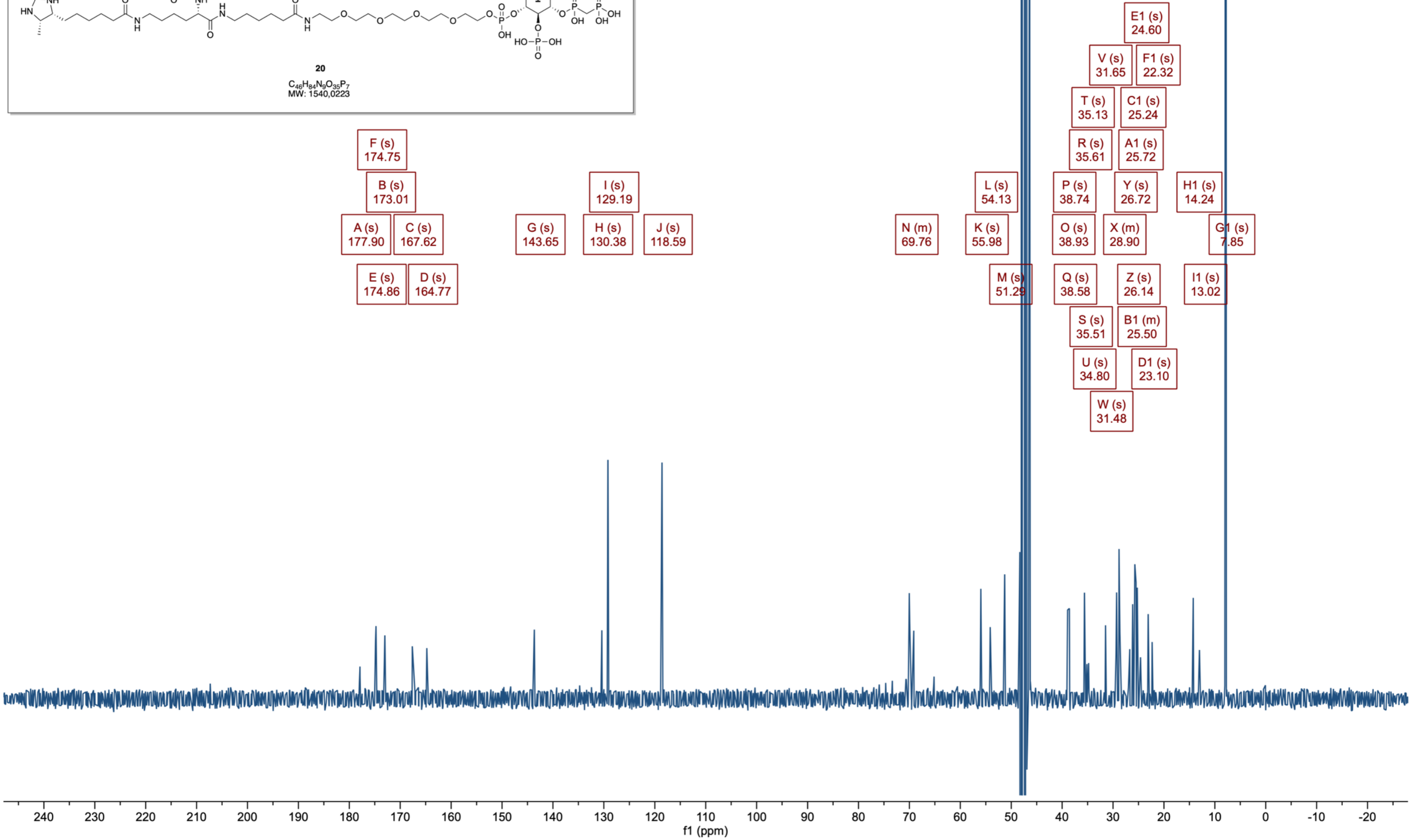

Compound 21: ^1H – NMR (MeOH-d_4, 400 MHz)

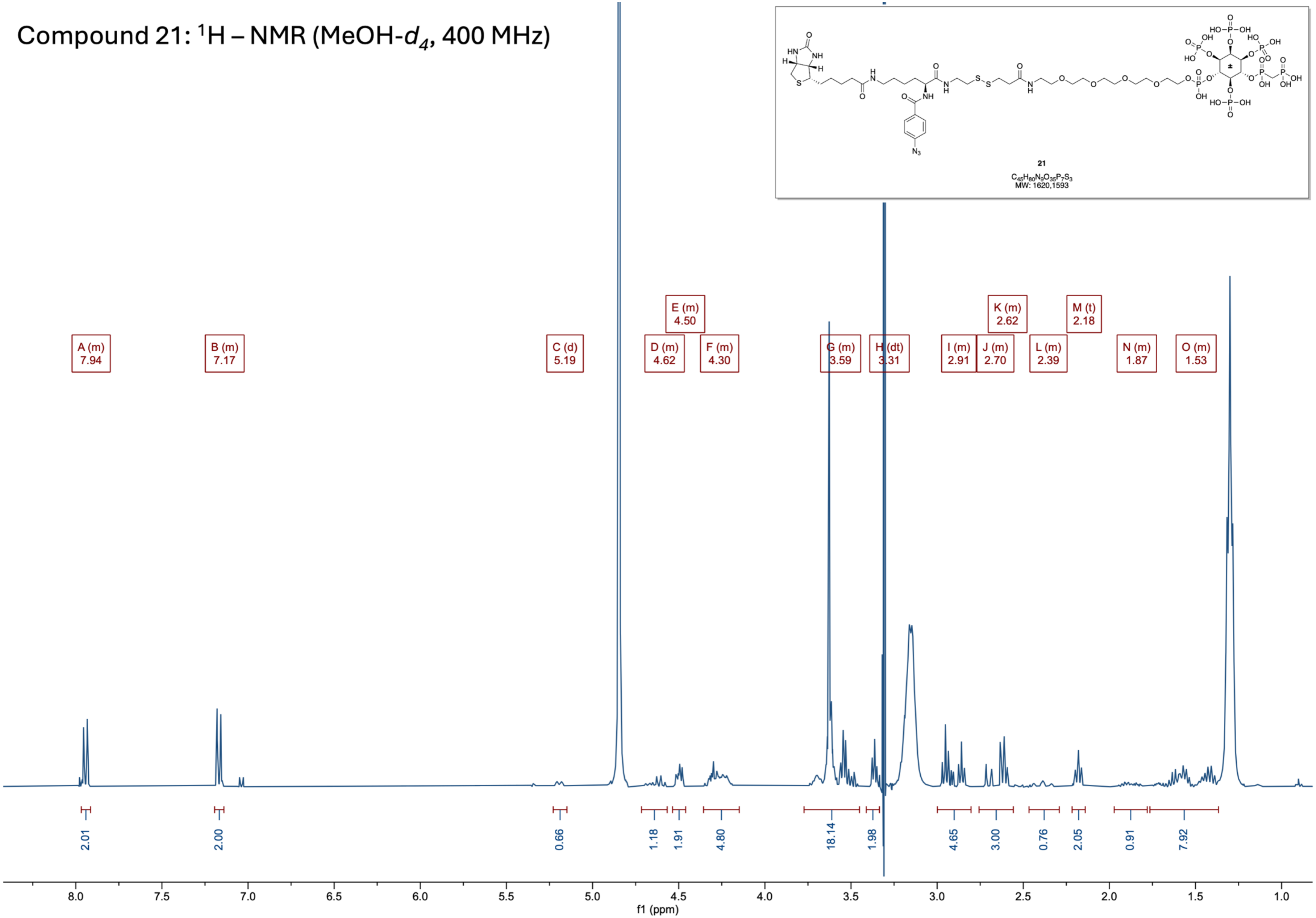

Compound 21: $^{31}P\{^1H\}$ – NMR (MeOH-d_4, 162 MHz)

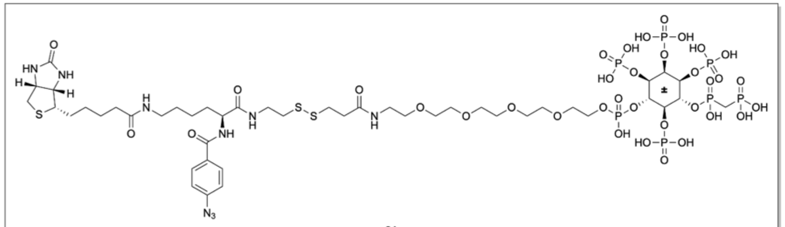

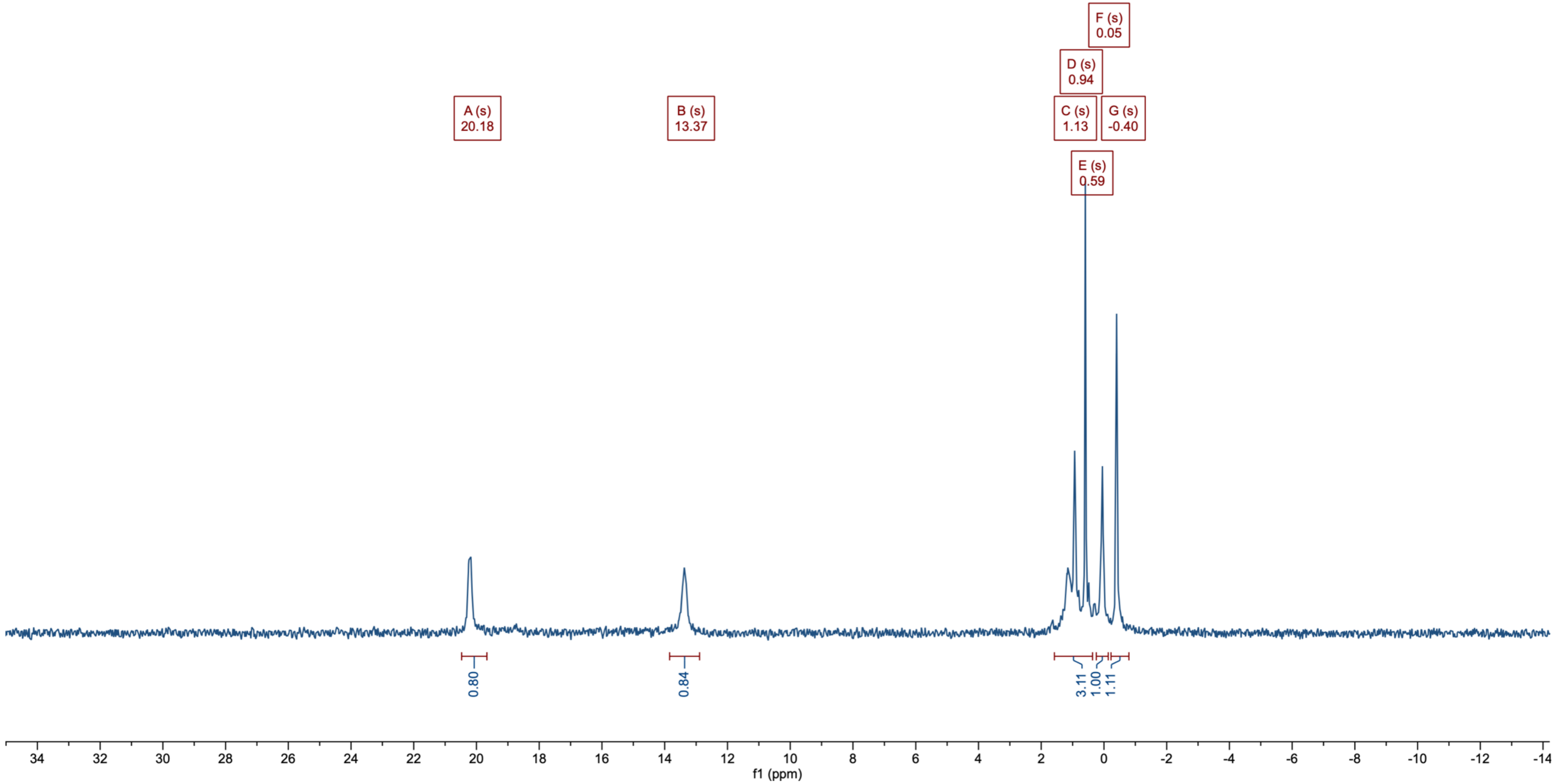

Compound 21: $^{13}C\{^{1}H\}$ – NMR (MeOH-d_4, 101 MHz)

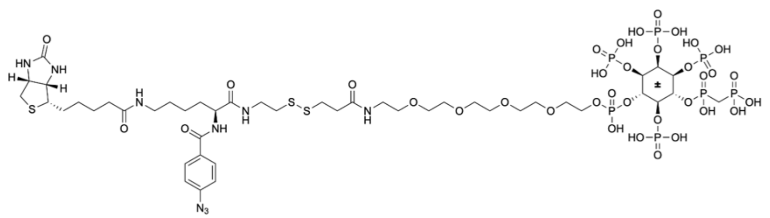

21

$C_{45}H_{80}N_9O_{35}P_7S_3$
MW: 1620,1593

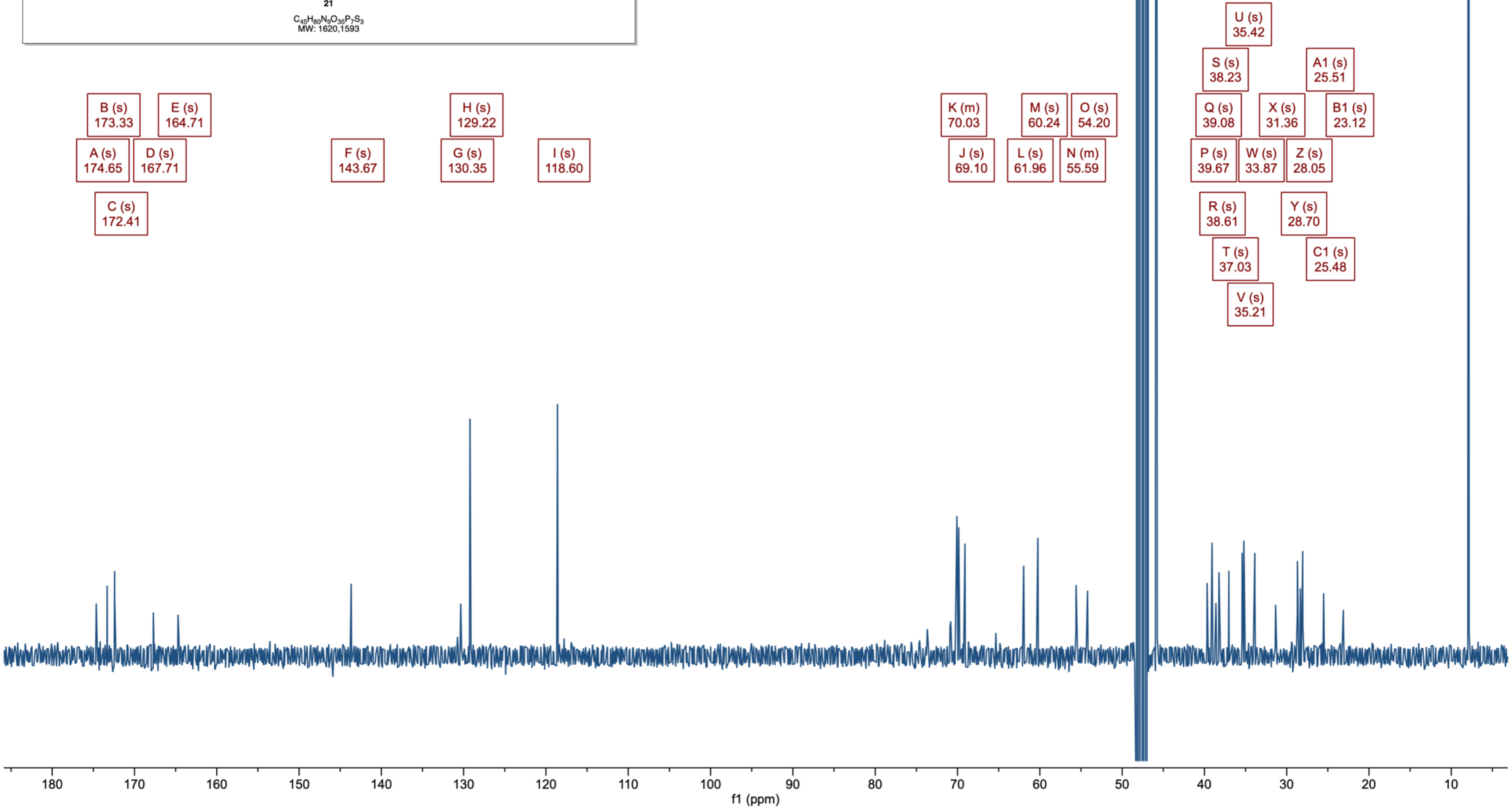

9 Mass Spectra, CE Electropherograms & HPLC

(Sorted according to molecule numbering)

Compound 8: HRMS (APCI) Analysis

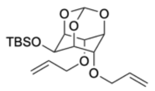

8
$C_{19}H_{32}O_6Si$
MW: 384,5440

D:\data_2023\rijea43thr1 3/9/2023 8:41:15 AM kr.a304
vap250

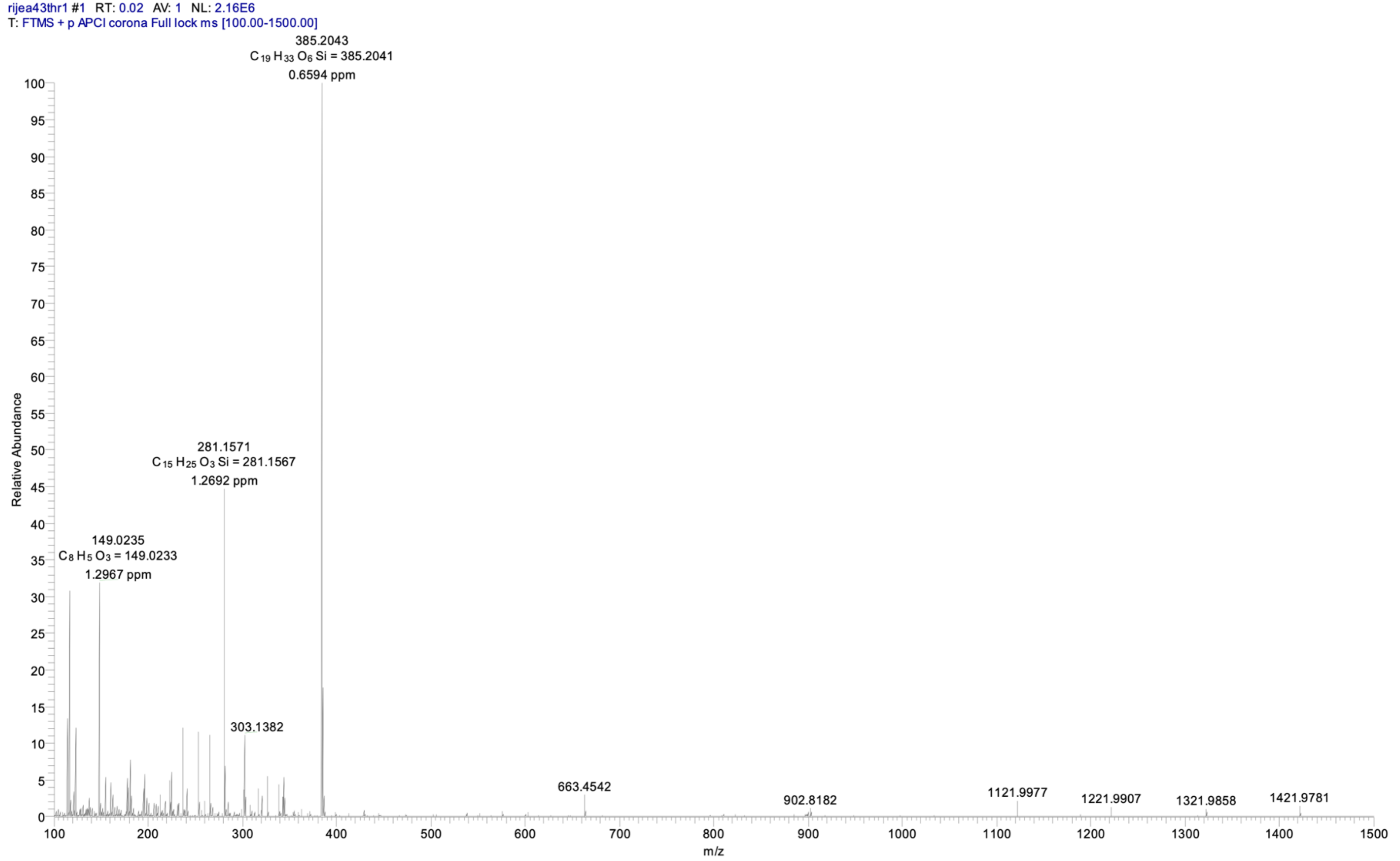

Compound 9: HRMS (APCI) Analysis

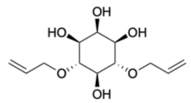

9

$C_{12}H_{20}O_6$
MW: 260,2860

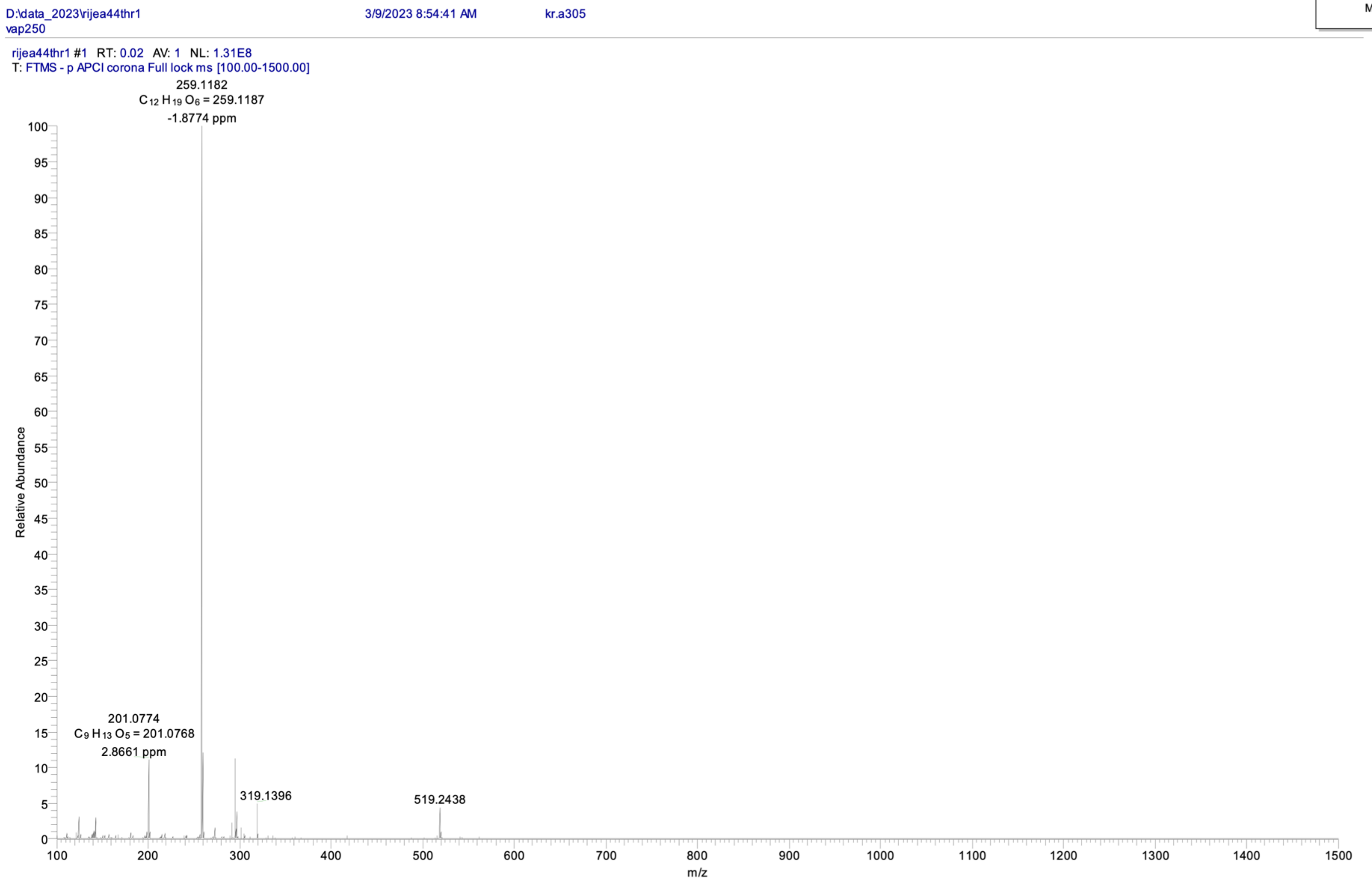

Compound 10: HRMS (ESI) Analysis

Analysis Report

Agilent | Trusted Answers

Sample Information

**Name**	kr.a450	**Data File Path**	D:\MassHunter\Data\2025\04\rijea75dis01.d
**Sample ID**		**Acq. Time (Local)**	4/7/2025 1:52:49 PM (UTC+02:00)
**Instrument**	QTOF-2	**Method Path (Acq)**	D:\MassHunter\Methods\Christoph\direkt0,2mlACN.m
**MS Type**	QTOF	**Version (Acq SW)**	6200 series TOF/6500 series Q-TOF 10.1 (48.0)
**Inj. Vol. (ul)**	0	**IRM Status**	Some ions missed
**Position**		**Method Path (DA)**	
**Plate Pos.**		**Target Source Path**	
**Operator**		**Result Summary**	

Sample Chromatograms

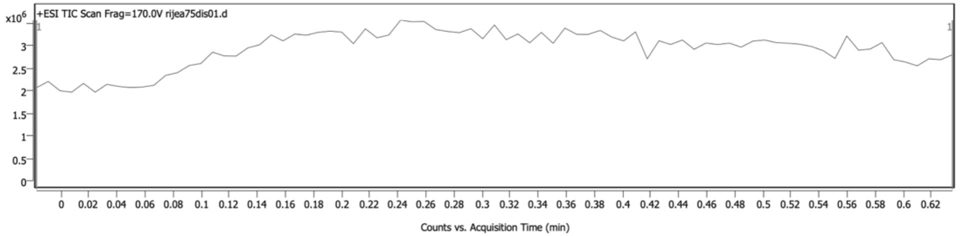

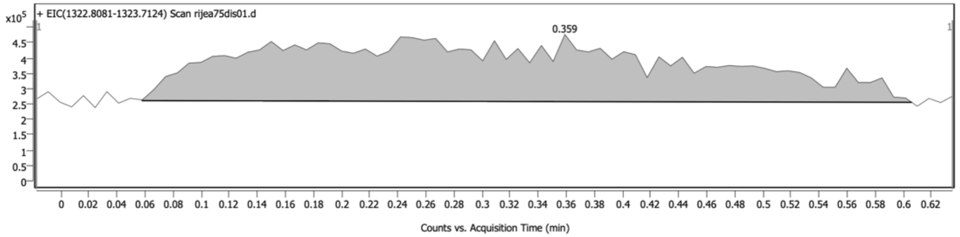

Chromatogram Peaks

Peak	Start	RT	End	Height	Area	Area %	SNR
1	0.058	0.359	0.606	218633	4384157	100.00	

Sample Spectra

+ Scan (rt: 0.200-0.250 min) Sub

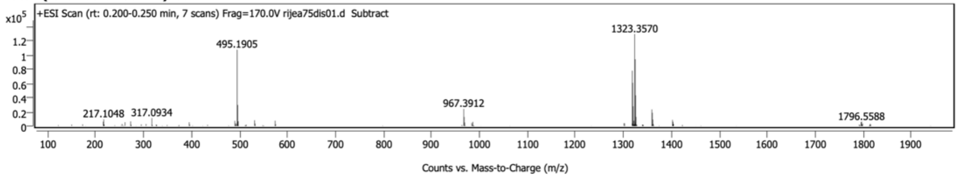

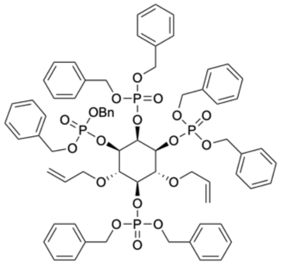

10

$C_{68}H_{72}O_{18}P_4$
MW: 1301,2010

Compound 11: HRMS (ESI) Analysis

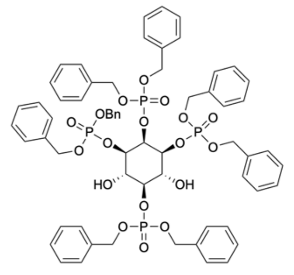

11

$C_{62}H_{64}O_{18}P_4$
MW: 1221,0710

Sample Chromatograms

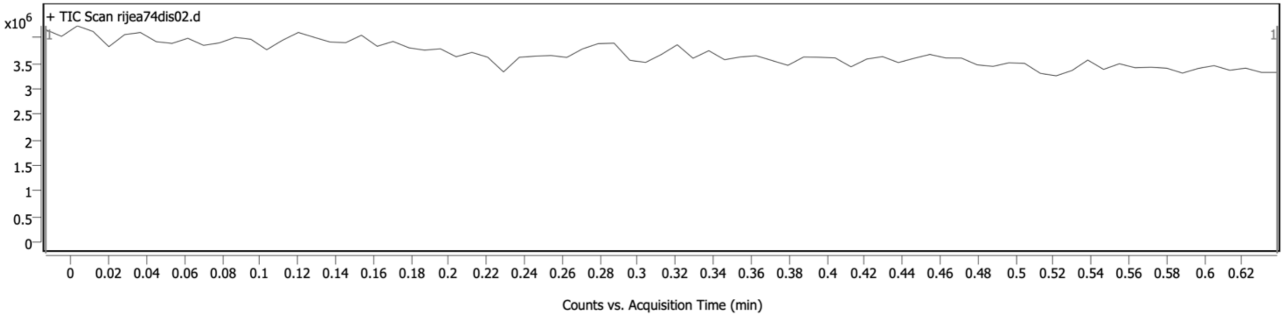

Sample Spectra

+ Scan (rt: -0.005-0.011 min) Sub

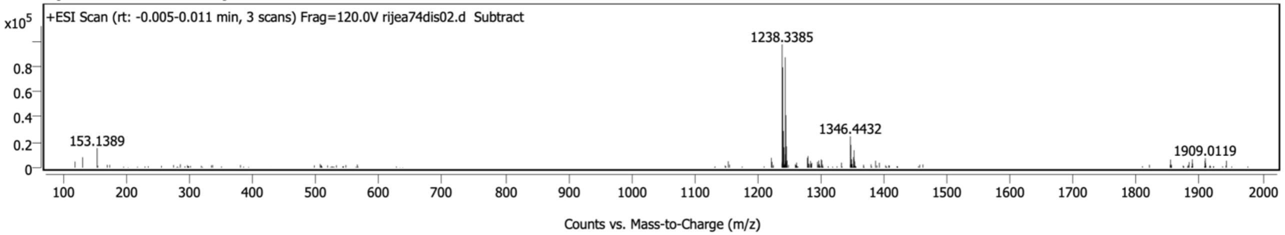

Compound 12: HRMS (ESI) Analysis

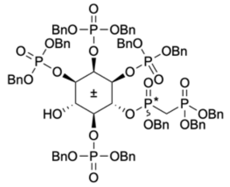

12

$C_{84}H_{86}O_{23}P_6$
MW: 1649,4316

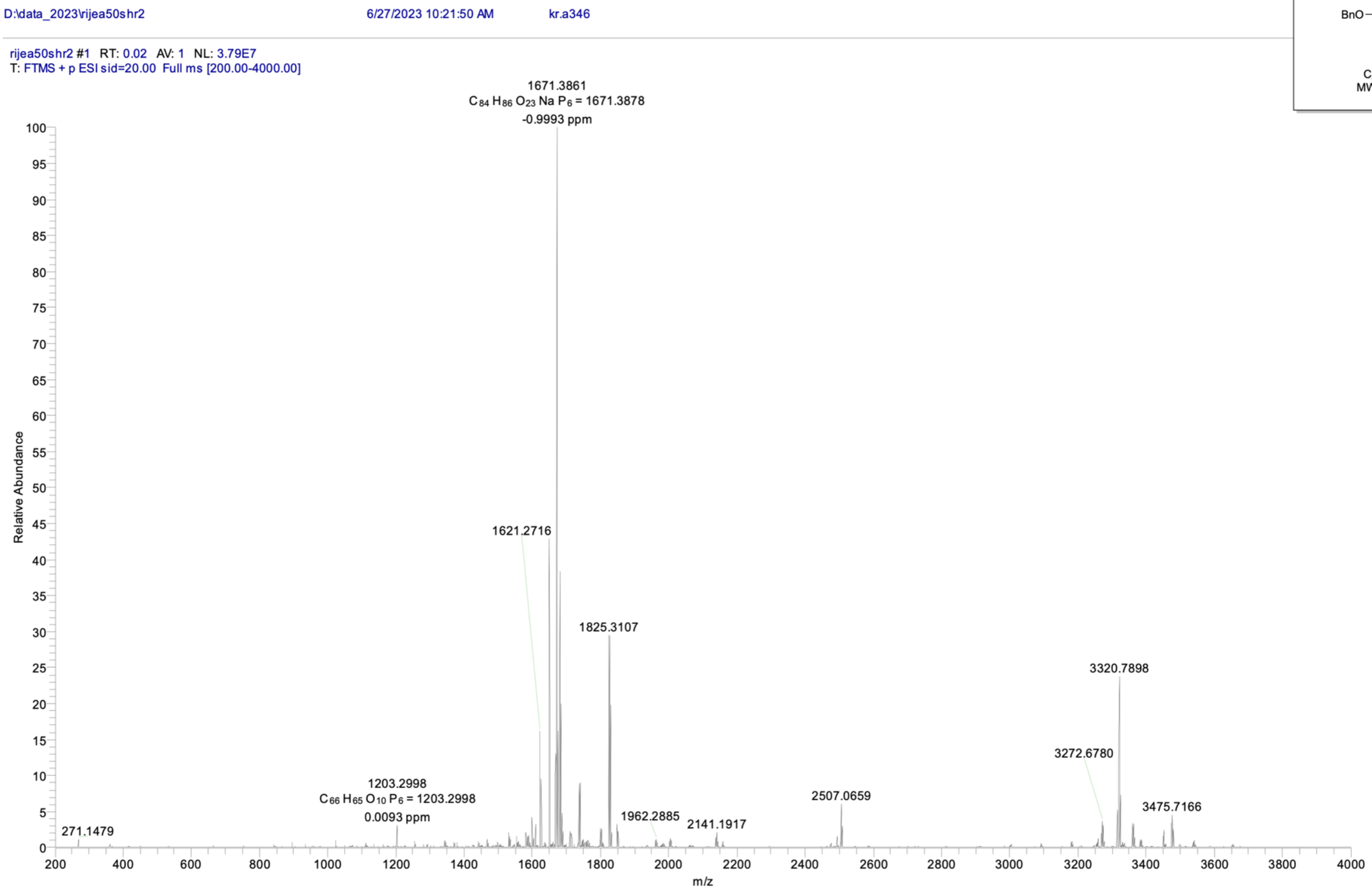

Compound 13: HRMS (ESI) Analysis

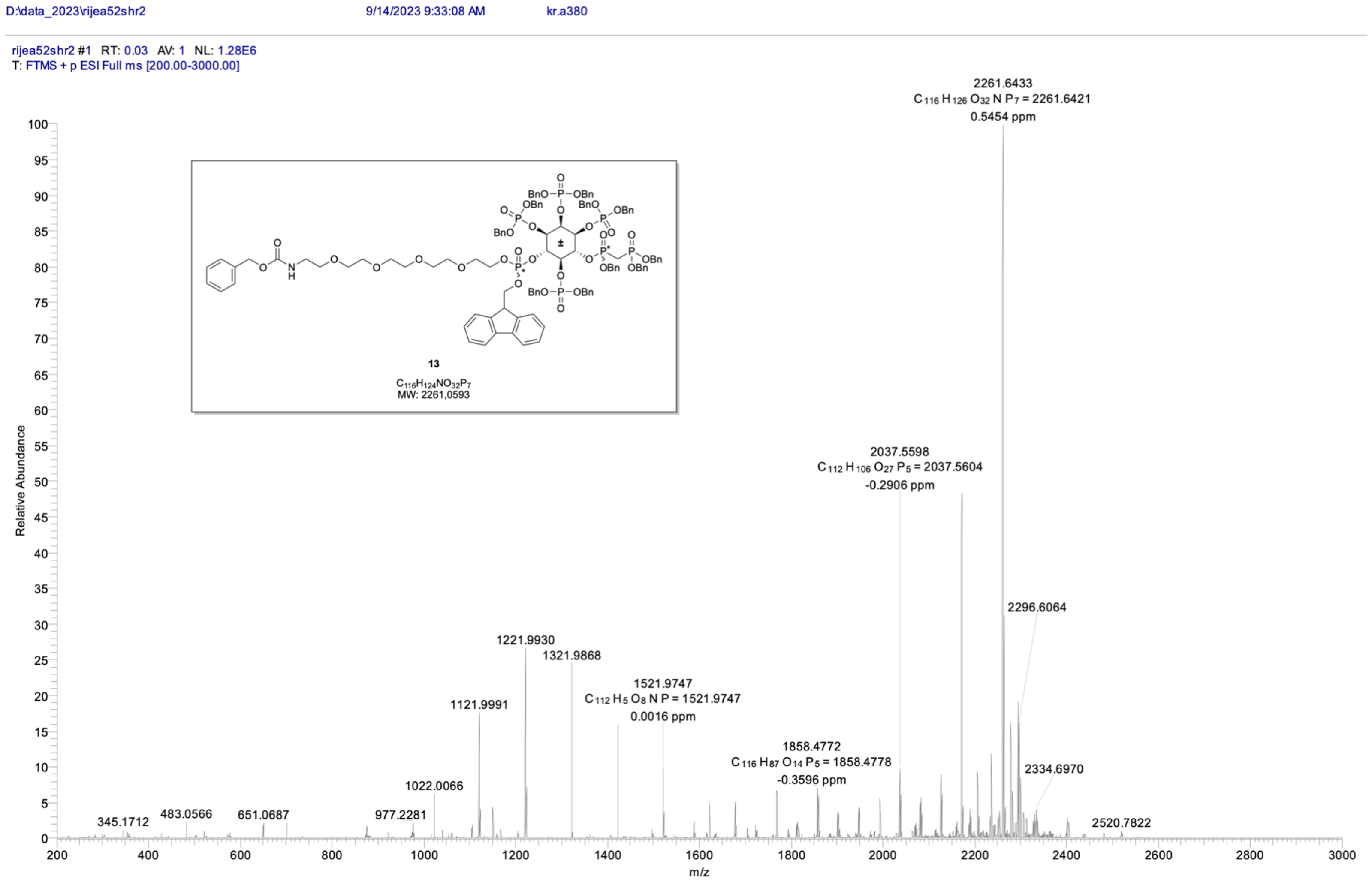

Compound 14: HRMS (ESI) Analysis

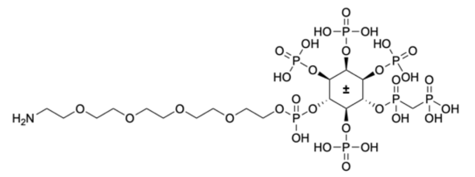

14

$C_{17}H_{42}NO_{30}P_7$
MW: 957,3163

**Sample Name**	KR-A381	**Position**	42	**Instrument Name**	QTOF-1
**User Name**		**Inj Vol**	Unknown / Injection Program	**InjPosition**	
**Sample Type**	Sample	**IRM Calibration Status**	Success	**Data Filename**	KR-A381.d
**ACQ Method**	Standard method.m	**Comment**		**Acquired Time**	16.09.2023 19:54:29 (UTC+02:00)

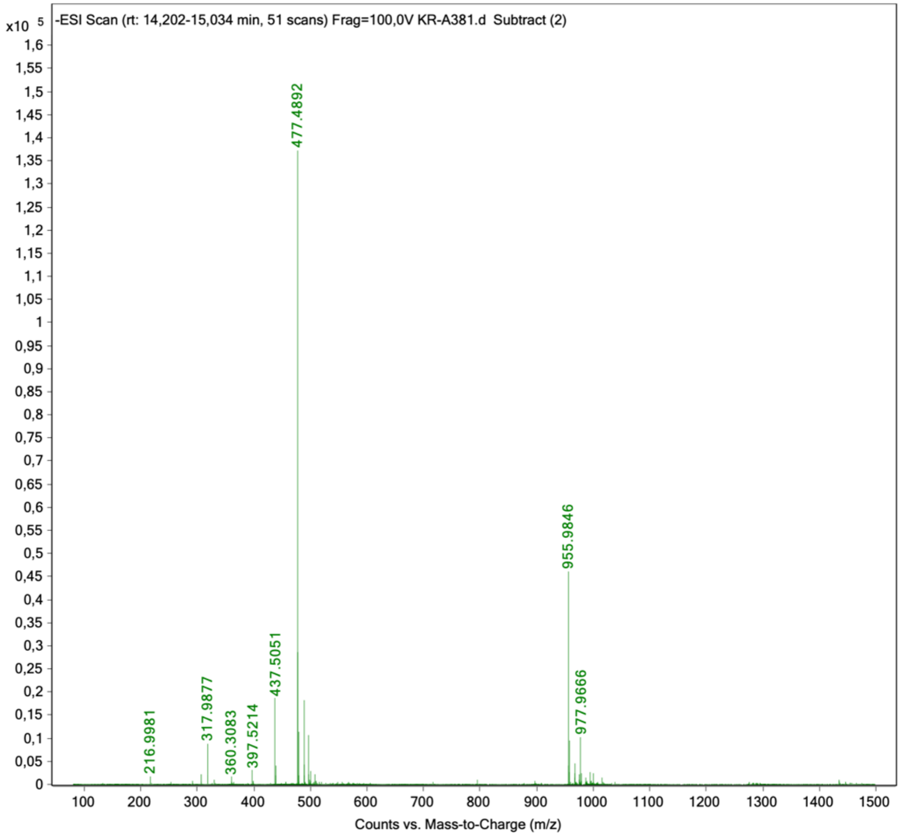

Compound 16: HRMS (ESI) Analysis

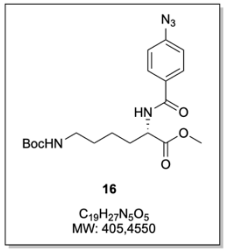

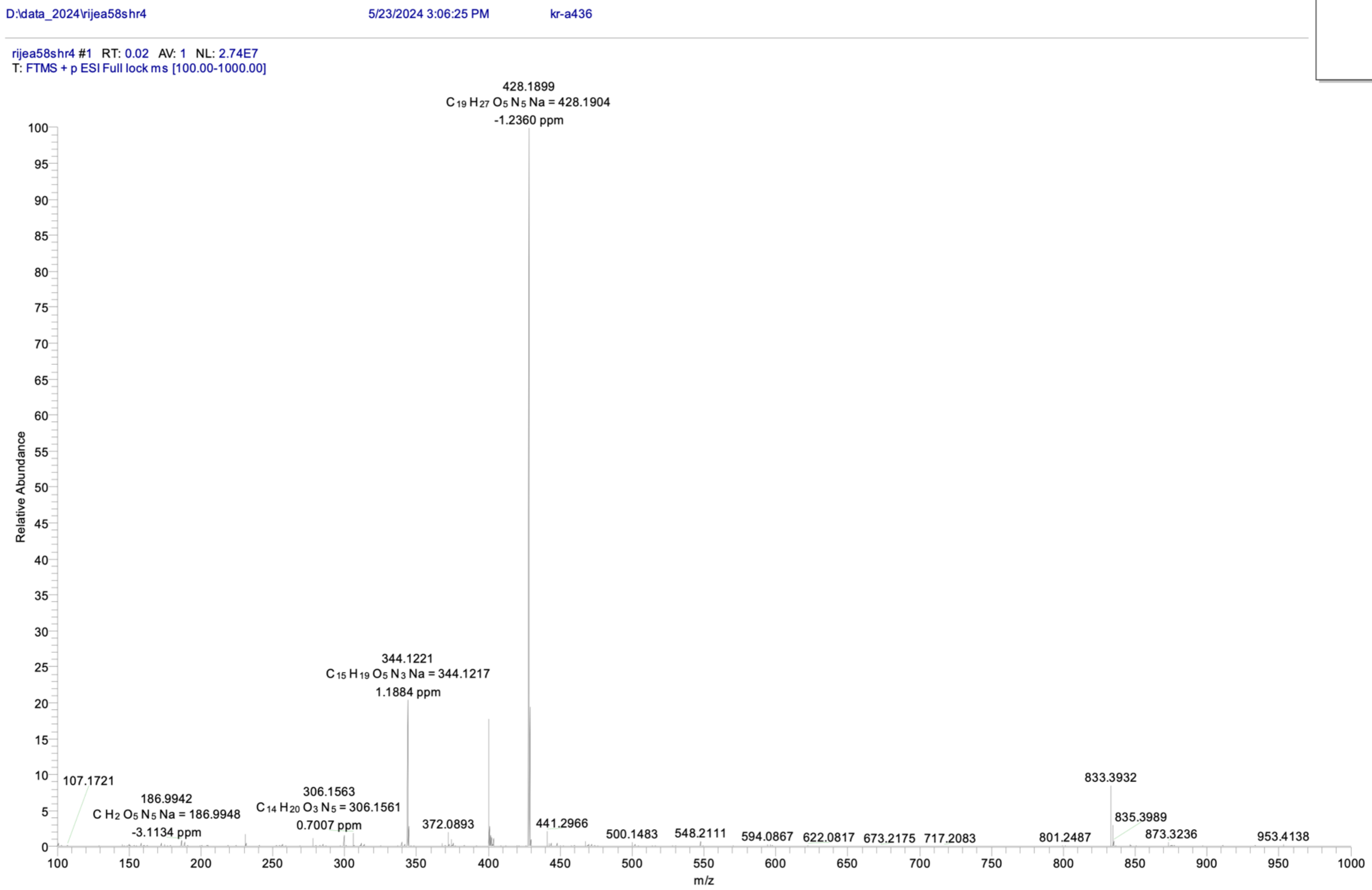

Compound 17: HRMS (ESI) Analysis

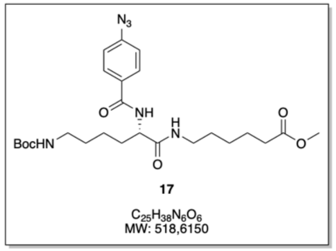

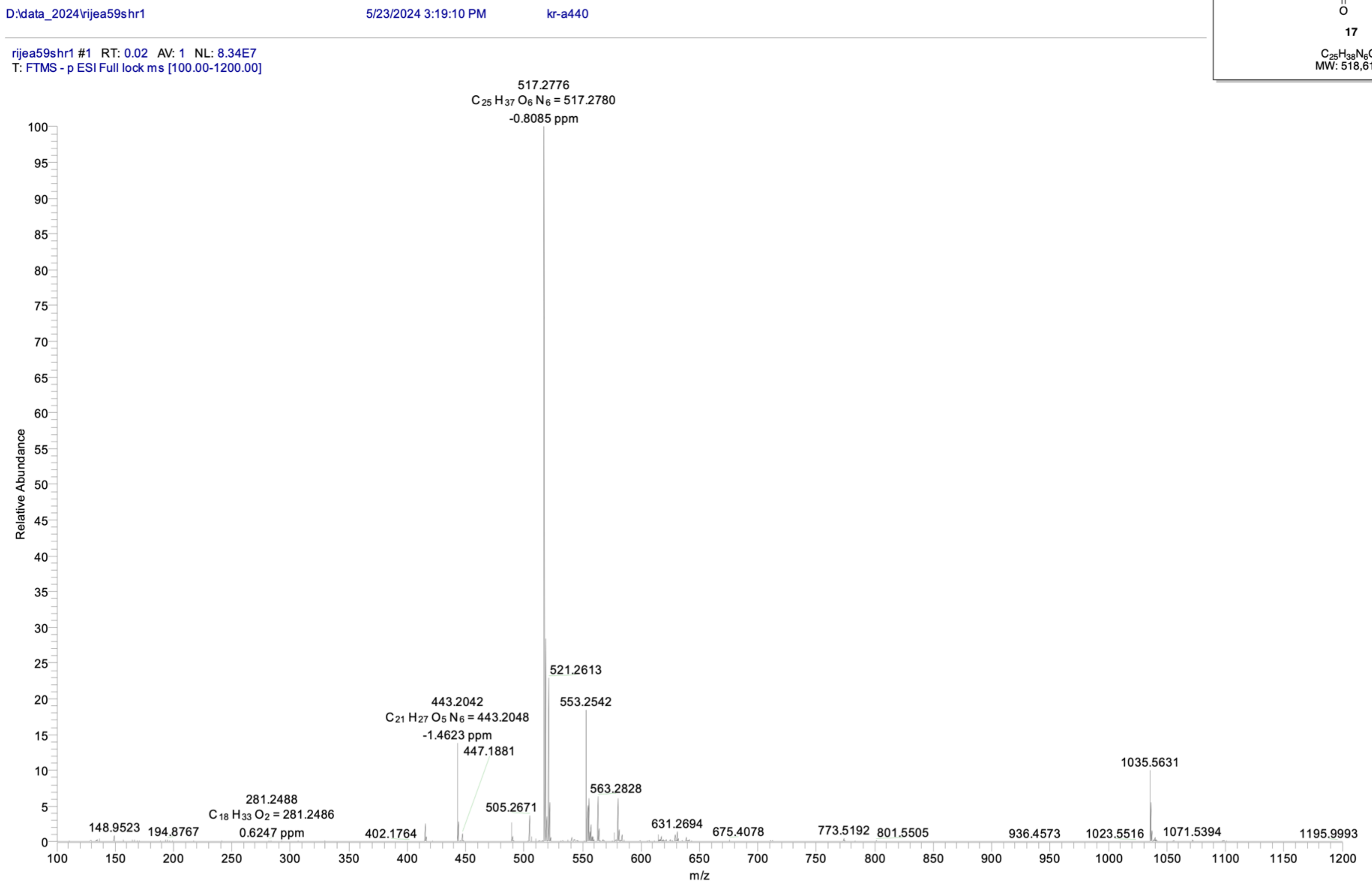

Compound 18: HRMS (ESI) Analysis

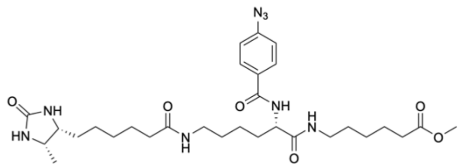

18

$C_{30}H_{46}N_8O_6$
MW: 614,7480

Analysis Report

Agilent | Trusted Answers

Sample Information

**Name**	kr-a441	**Data File Path**	D:\MassHunter\Data\2025\04\rijea73dis01.d
**Sample ID**		**Acq. Time (Local)**	4/2/2025 3:35:30 PM (UTC+02:00)
**Instrument**	QTOF-2	**Method Path (Acq)**	D:\MassHunter\Methods\Christoph\direkt0,2mlACN.m
**MS Type**	QTOF	**Version (Acq SW)**	6200 series TOF/6500 series Q-TOF 10.1 (48.0)
**Inj. Vol. (ul)**	0	**IRM Status**	All ions missed
**Position**		**Method Path (DA)**	
**Plate Pos.**		**Target Source Path**	
**Operator**		**Result Summary**	

Sample Chromatograms

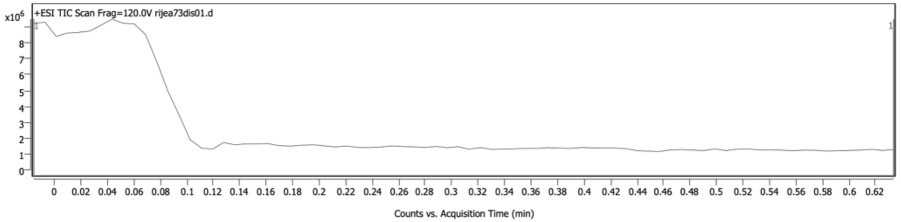

Sample Spectra

+ Scan (rt: 0.027-0.060 min) Sub

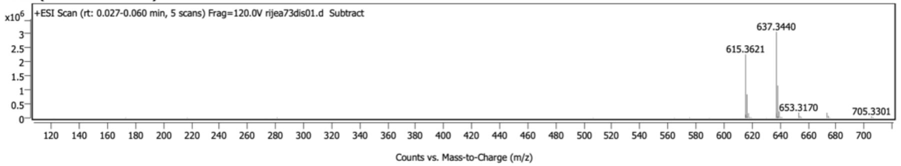

Spectrum Peaks

m/z	Z	Abund	Abund %	m/z (Calc)	Diff (ppm)	Ion Species	Formula	Ion Type
615.3621	1	2271542	73.92	615.3613	1.22	(M+H)+	C30 H46 N8 O6	
616.3648	1	841146	27.37	616.3642	0.91	(M+H)+	C30 H46 N8 O6	
617.3669	1	173511	5.65	617.3668	0.10	(M+H)+	C30 H46 N8 O6	
618.3693	1	31521	1.03	618.3694	-0.07	(M+H)+	C30 H46 N8 O6	
637.3440	1	3072784	100.00	637.3433	1.23	(M+Na)+	C30 H46 N8 O6	
638.3468	1	1158552	37.70	638.3461	1.08	(M+Na)+	C30 H46 N8 O6	
639.3491	1	240628	7.83	639.3488	0.51	(M+Na)+	C30 H46 N8 O6	
640.3512	1	41502	1.35	640.3513	-0.23	(M+Na)+	C30 H46 N8 O6	
615.4147	1	115485	3.76					
616.4184	1	42547	1.38					
637.3968	1	163628	5.33					
638.4011	1	58200	1.89					
653.3170	1	195182	6.35					
654.3200	1	68973	2.24					
673.4143	1	187053	6.09					
674.4166	1	72719	2.37					
705.3301		57257	1.86					

Spectrum Identification Table

Best	ID Source	Name	Formula	Species	m/z	Diff (ppm)	CAS	Score	Score (Lib)	Score (DB)	Score (MFG)	Lib/DB
No	MFG		C30 H46 N8 O6	(M+H)+	615.3621	1.07		99.09			99.09	
No	MFG		C30 H46 N8 O6	(M+Na)+	637.3440	1.18		98.85			98.85	

Sample Information

**Name**	kr-a441	**Data File Path**	D:\MassHunter\Data\2025\04\rijea73dis02.d
**Sample ID**		**Acq. Time (Local)**	4/2/2025 3:36:59 PM (UTC+02:00)
**Instrument**	QTOF-2	**Method Path (Acq)**	D:\MassHunter\Methods\Christoph\direkt0,2mlACN.m
**MS Type**	QTOF	**Version (Acq SW)**	6200 series TOF/6500 series Q-TOF 10.1 (48.0)
**Inj. Vol. (ul)**	0	**IRM Status**	Some ions missed
**Position**		**Method Path (DA)**	
**Plate Pos.**		**Target Source Path**	
**Operator**		**Result Summary**	

MassHunter Qualitative Analysis | Page 1 of 2 | Generated at 3:39 PM on 4/2/2025

Compound 19: HRMS (ESI) Analysis

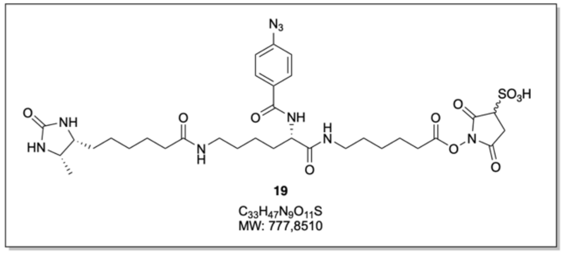

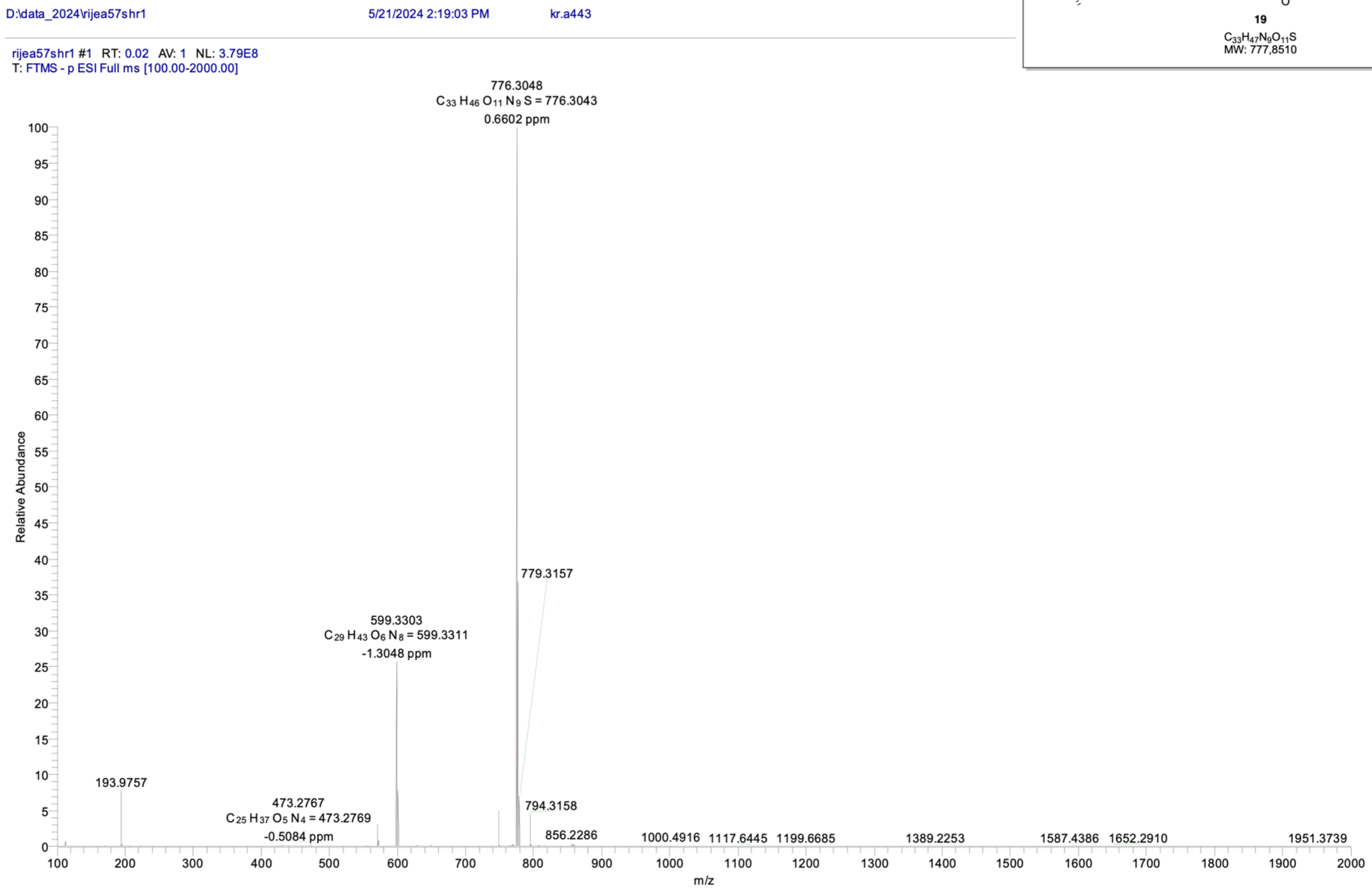

Compound 20: HRMS (ESI) Analysis

Analysis Report

Agilent Trusted Answers

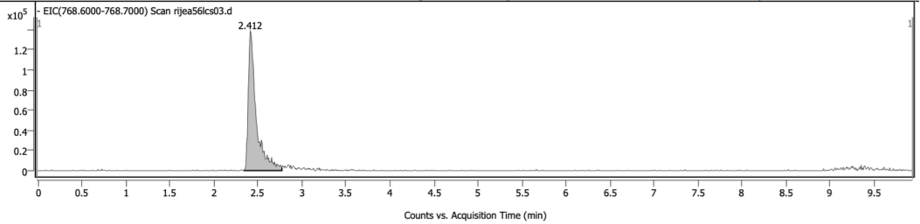

Chromatogram Peaks

Peak	Start	RT	End	Height	Area	Area %	SNR
1	2.338	2.412	2.772	138777	878178	100.00	

Sample Spectra

- Scan (rt: 2.379-2.546 min) Sub Peak 1 from - EIC(768.6000-768.7000) Scan

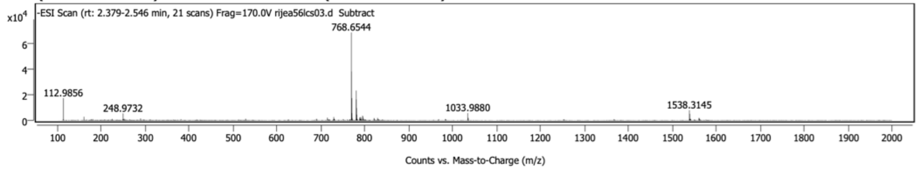

Spectrum Peaks

m/z	Z	Abund	Abund %	m/z (Calc)	Diff (ppm)	Ion Species	Formula	Ion Type
768.6544	2	69065	100.00	768.6544	0.02	(M-2H)-2	C46 H84 N9 O35 P7	
769.1559	2	38364	55.55	769.1559	0.02	(M-2H)-2	C46 H84 N9 O35 P7	
769.6570	2	17442	25.25	769.6571	-0.14	(M-2H)-2	C46 H84 N9 O35 P7	
770.1586	2	5688	8.24	770.1584	0.30	(M-2H)-2	C46 H84 N9 O35 P7	
770.6581	2	1883	2.73	770.6596	-1.91	(M-2H)-2	C46 H84 N9 O35 P7	
1033.9880	1	5745	8.32	1033.9870	0.97	(M-H)-	C35 H30 N8 O16 P7	
1034.9911	1	1939	2.81	1034.9900	1.04	(M-H)-	C35 H30 N8 O16 P7	
1538.3145	1	8035	11.63	1538.3160	-0.98	(M-H)-	C46 H84 N9 O35 P7	
1539.3178	1	5105	7.39	1539.3191	-0.80	(M-H)-	C46 H84 N9 O35 P7	
112.9856		17479	25.31					
160.0153		2846	4.12					
248.9732		5479	7.93					
288.9497		1544	2.24					
714.3572		2133	3.09					
728.6712		2671	3.87					
768.7146		1576	2.28					
779.6401	2	23427	33.92					
780.1414	2	16464	23.84					
780.6406	2	9638	13.95					
781.1410	2	4459	6.46					
787.6287		2294	3.32					
789.6770		2632	3.81					
790.6319		2120	3.07					
794.3145	1	3701	5.36					
795.3193	1	2920	4.23					
796.3203	1	1420	2.06					
820.6401		1796	2.60					
828.6280		1896	2.74					
1560.2886	1	1879	2.72					
1561.2918	1	1693	2.45					

Spectrum Identification Table

Best	ID Source	Name	Formula	Species	m/z	Diff (ppm)	CAS	Score	Score (Lib)	Score (DB)	Score (MFG)	Lib/DB
No	MFG		C46 H84 N9 O35 P7	(M-2H)-2	768.6544	-0.02		98.55			98.55	
No	MFG		C35 H30 N8 O16 P7	(M-H)-	1033.9880	1.00		83.69			83.69	
No	MFG		C36 H36 N O21 P7	(M-H)-	1033.9880	0.89		83.15			83.15	
No	MFG		C46 H84 N9 O35 P7	(M-H)-	1538.3145	-0.79		82.83			82.83	

MassHunter Qual 10.0
(End of Report)

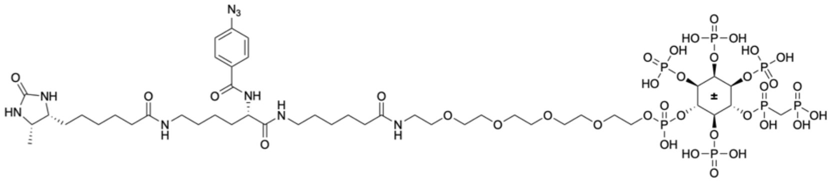

20

$C_{46}H_{84}N_9O_{35}P_7$
MW: 1540,0223

Compound 20: HPLC Analysis

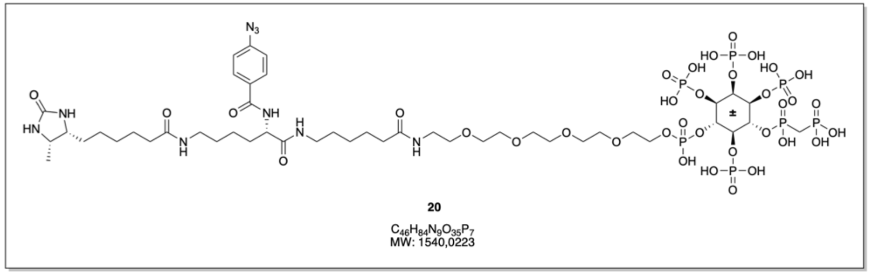

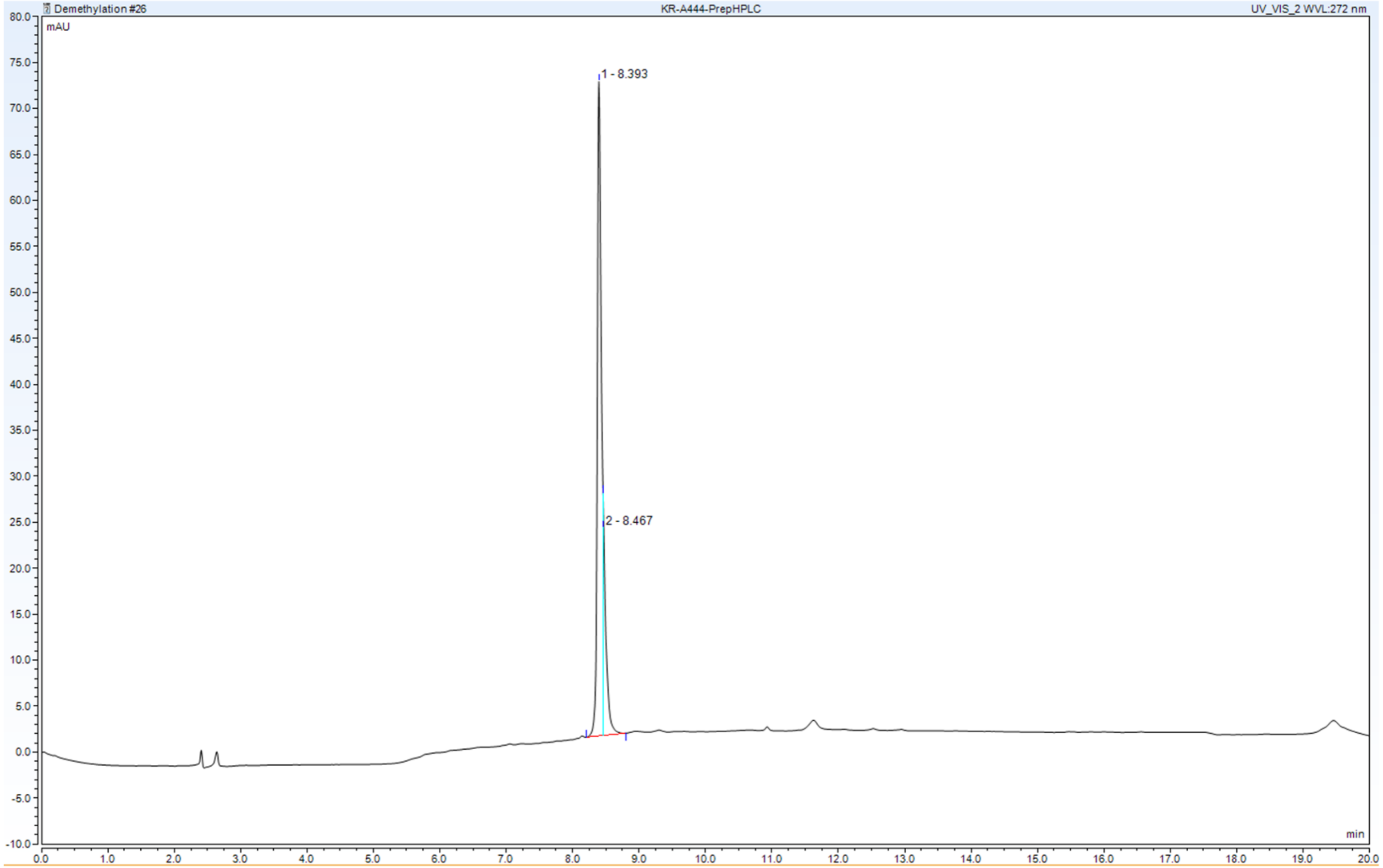

Compound 21: HRMS (ESI) Analysis

Analysis Report

Agilent | Trusted Answers

Sample Information

**Name**	kr.a383	**Data File Path**	D:\MassHunter\Data\2023\09\rijea53dis01.d
**Sample ID**	lc neg.esi	**Acq. Time (Local)**	9/20/2023 2:52:40 PM (UTC+02:00)
**Instrument**	QTOF-2	**Method Path (Acq)**	D:\MassHunter\Methods\Christoph\esi-neg90a-100D(8-10)700c20.m
**MS Type**	QTOF	**Version (Acq SW)**	6200 series TOF/6500 series Q-TOF 10.1 (48.0)
**Inj. Vol. (ul)**	0.2	**IRM Status**	Success
**Position**		**Method Path (DA)**	
**Plate Pos.**		**Target Source Path**	
**Operator**		**Result Summary**	

Sample Chromatograms

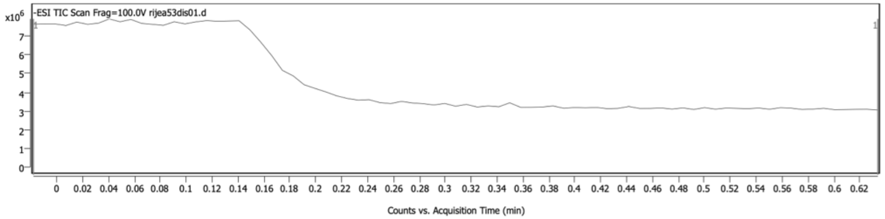

Sample Spectra

- Scan (rt: 0.074-0.116 min) Sub (2)

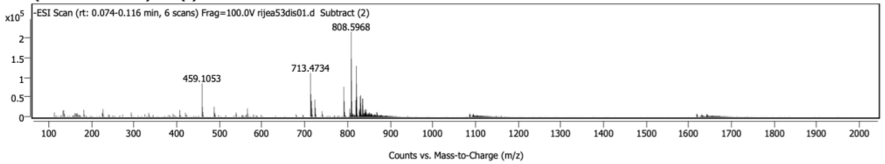

Spectrum Peaks

m/z	Z	Abund	Abund %	m/z (Calc)	Diff (ppm)	Ion Species	Formula	Ion Type
808.5968	2	215185	100.00	808.5968	-0.02	(M-2H)-2	C45 H80 N9 O35 P7 S3	
809.0984	2	146723	68.18	809.0983	0.17	(M-2H)-2	C45 H80 N9 O35 P7 S3	
809.5978	2	90836	42.21	809.5977	0.04	(M-2H)-2	C45 H80 N9 O35 P7 S3	
810.0980	2	38882	18.07	810.0984	-0.40	(M-2H)-2	C45 H80 N9 O35 P7 S3	
459.1053		84204	39.13					
713.4734	1	110827	51.50					
714.4770	1	56658	26.33					
715.4723	1	39892	18.54					
723.5018		43490	20.21					
791.4893	1	75951	35.30					
792.4934	1	39380	18.30					
819.5858		42182	19.60					
820.5761	2	128810	59.86					
821.0775	2	83185	38.66					
821.5769	2	50201	23.33					
829.5806		51131	23.76					
830.5796	2	54574	25.36					
831.0805	2	34453	16.01					
835.0527	2	44708	20.78					
835.5550	2	46508	21.61					

Spectrum Identification Table

Best	ID Source	Name	Formula	Species	m/z	Diff (ppm)	CAS	Score	Score (Lib)	Score (DB)	Score (MFG)	Lib/DB
No	MFG		C45 H80 N9 O35 P7 S3	(M-2H)-2	808.5968	0.03		96.63			96.63	

Sample Information

MassHunter Qualitative Analysis | Page 1 of 3 | Generated at 3:16 PM on 9/20/2023

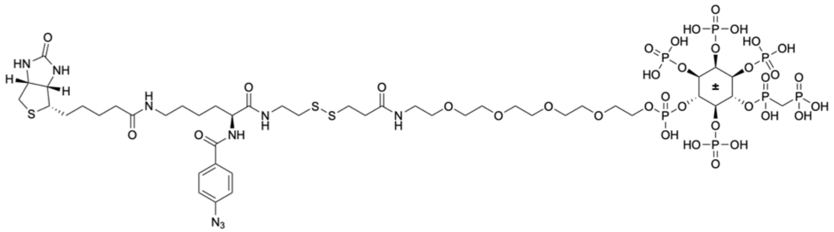

21

$C_{45}H_{80}N_9O_{35}P_7S_3$
MW: 1620,1593

Compound 21: HPLC Analysis

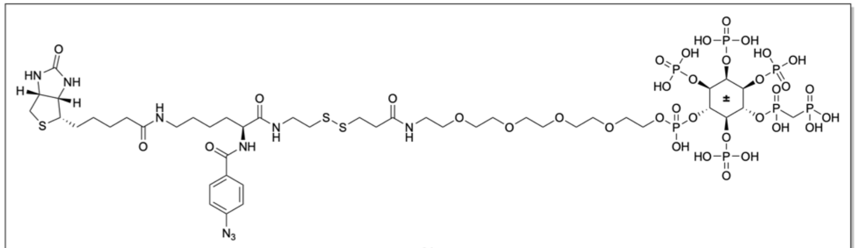

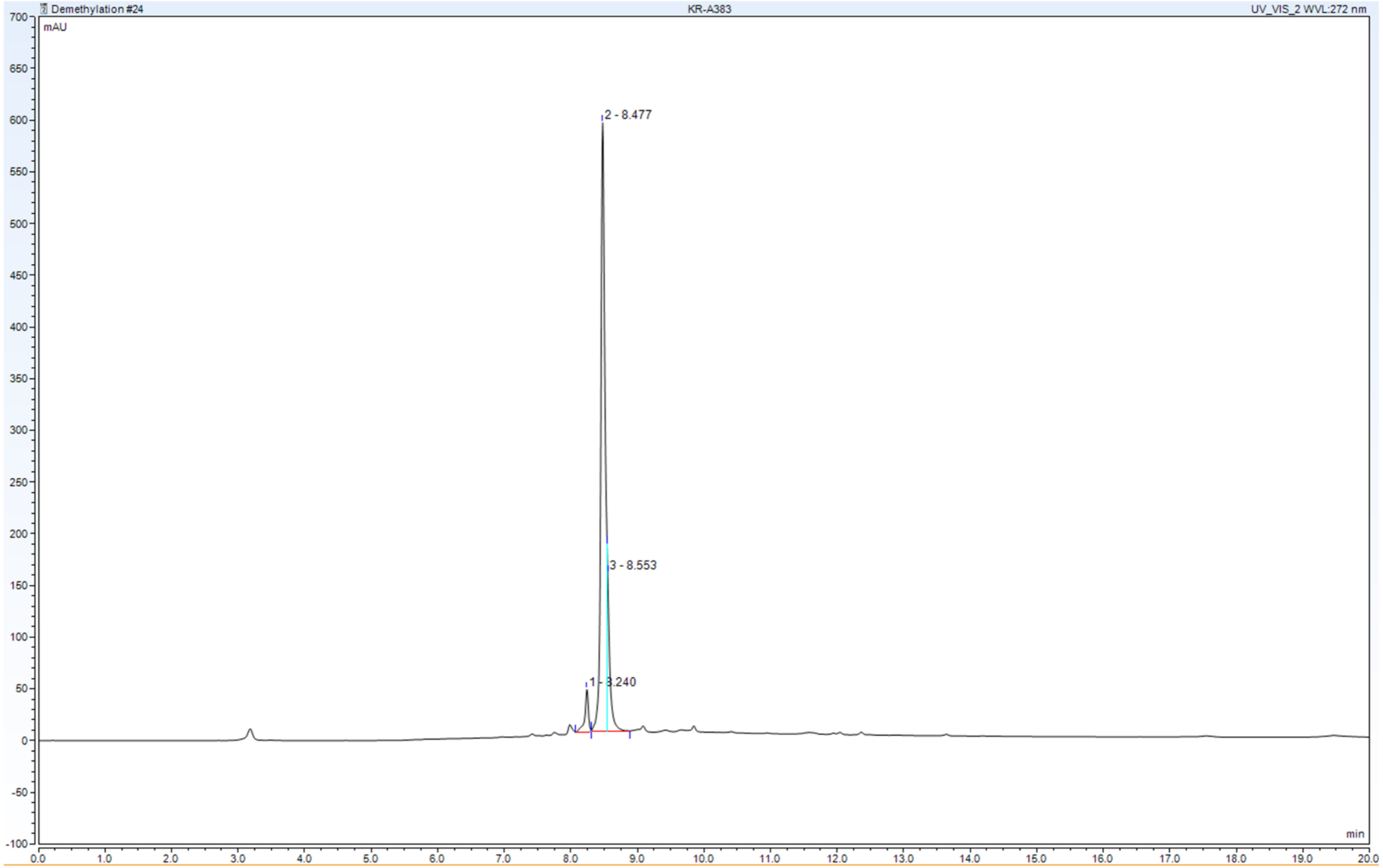

Supplement: Supplementary file 1 — Supporting File 1: advs74650‐sup‐0001‐SuppMat.pdf. [file ADVS-13-e24290-s002.pdf]
